# A revision of the shore-fly genus *Lamproclasiopa* Hendel (Diptera, Ephydridae)

**DOI:** 10.3897/zookeys.631.10718

**Published:** 2016-11-14

**Authors:** Daniel N. R. Costa, Wayne N. Mathis, Luciane Marinoni

**Affiliations:** 1 Departamento de Zoologia, Universidade Federal do Paraná, Jardim das Américas, 81531-980 – Curitiba, Paraná, Brazil; 2 Department of Entomology, PO Box 37012, MRC 169; Smithsonian Institution, Washington, D.C. 20013-7012, USA

**Keywords:** Diptera, Ephydridae, New World tropics, Indian Subcontinent, taxonomic changes, Lamproclasiopa

## Abstract

The species of the genus *Lamproclasiopa* Hendel are revised, including 13 new species (type locality in parenthesis): *Lamproclasiopa aliceae* (United States. New Mexico. Grant: Silver City (Big Ditch; 32°46.4'N, 108°16.5'W; 1790 m)), *Lamproclasiopa argentipicta* (Costa Rica. San José. Zurquí de Moravia (10°2.8'N, 84°0.6'W)), *Lamproclasiopa auritunica* (Bolívia. Oruro: Paznã (S. of the town; 18°36.2'S, 66°54.7'W, 3750 m).), *Lamproclasiopa brunnea* (Costa Rica. San José. Zurquí de Moravia (10°2.8'N, 84°0.6'W)), *Lamproclasiopa caligosa* (Chile. Osorno: Anticura (1 km W; 40°39'S, 72°10'W; 430 m)), *Lamproclasiopa curva* (Chile. Los Lagos: Chiloé Island, Chepu (on seashore; 42°5'S, 73°59.65'W)), *Lamproclasiopa ecuadoriensis* (Ecuador. Orellana: Río Tiputini Biodiversity Station (0°38.2'S, 76°8.9'W)), *Lamproclasiopa furvitibia* (Costa Rica. San José. Zurquí de Moravia (10°2.8'N, 84°0.6'W)), *Lamproclasiopa lapaz* (Bolívia. La Paz: La Paz (6 km NE; 16°25.7'S, 68°04.3'W; 4130m)), *Lamproclasiopa mancha* (Brazil. Paraná: Curitiba, Universidade Federal do Paraná, Reserva Biológica (25°26.9'S, 49°14'W; 915 m)), *Lamproclasiopa triangularis* (Peru. Madre de Dios: Río Manu, Pakitza (11°56.6'S, 71°16.9'W; 250 m)), *Lamproclasiopa xanthocera* (Brazil. Paraná. Curitiba, Universidade Federal do Paraná, Reserva Biológica (25°26.9'S, 49°14'W; 915 m)), *Lamproclasiopa zerafael* (Brazil. Amazonas: Reserva Ducke (02°55.8'S, 59°58.5'W; 40 m)). All known species are described with an emphasis on structures of the male terminalia, which are fully illustrated. Detailed locality data and distribution maps for all species are provided. For perspective and to facilitate genus-group and species-group recognition, the tribe Discocerinini is diagnosed and a key to genera in the New World is provided.

## Introduction

The need for revision of *Lamproclasiopa* Hendel 1933 is abundantly apparent. Over half of the included species (13 of 24 species) were undescribed previous to this paper, and the genus has never been treated comprehensively. We also document in this revision how the nomenclatural history of the genus is reflective of changing concepts in classification that resulted from an improved understanding of the tribal phylogeny (Zatwarnicki and Mathis 2001, Zatwarnicki et al. 2016).

While change is seemingly inevitable in science, including the classification of shore flies, an objective of taxonomy/systematics is a stable classification and nomenclature. These objectives are best achieved with discovery of accurate and well-documented phylogenetic relationships of the included taxa, as well as their accurate and detailed descriptions. Although we focus primarily on descriptive details in this revision, including keys, illustrations and photographs, we also provide a brief phylogenetic framework for the genus and to a lesser degree for species groups within the genus.

Hendel (1933) first described *Lamproclasiopa* as a subgenus within the genus *Discocerina* Macquart and included *Discocerina facialis* Hendel, which he described in the same paper, as the type species. Hendel (p. 80) also included *Discocerina chalybea* Hendel in *Lamproclasiopa*. Hendel’s name for *Discocerina facialis*, however, was preoccupied (Williston 1896) and was corrected when Wirth (1968: 7) proposed *Lamproclasiopa hendeli* as a replacement new name. Cresson (1942, 1945, 1946) continued usage of *Lamproclasiopa* as a subgenus but changed its concept to include those species of *Discocerina* with a bare parafacial and three facial setae. The type species of *Lamproclasiopa* (*Lamproclasiopa hendeli*), however, only has two facial setae. Toward the end of Cresson’s illustrious career (1942: 116), he described yet another subgenus within *Discocerina*, *Basila* (type species: *Ditrichophora nadineae* Cresson) for those species with two facial setae and a bare parafacial. Cresson’s precedent was adopted by Wirth in his catalogs for the shore-fly fauna of the New World (1965, 1968) and by Mathis and Zatwarnicki (1995) in their world catalog. Zatwarnicki and Mathis (2001) recharacterized *Lamproclasiopa* as part of their phylogenetic revision of the tribe Discocerinini, and the former subgenus was accorded generic status. Their recharacterization essentially reverted back to Hendel’s original diagnosis of two facial setae, but they also included a bare parafacial. Their revised concept of *Lamproclasiopa* included 10 New World species and one species, *Lamproclasiopa laevior* (Cresson), from the Indian Subcontinent.

Cresson (1946) published the first synopsis of Neotropical Discocerinini, which was the last in a series of synopses for the region that he produced. Cresson included 28 species in four genera, and some of these species were based on tentative identifications. Over twenty years later, Wirth (1968) produced the first catalog of shore flies from the Neotropical Region and listed 30 species in the same four genera. In recent revisions (Mathis and Zatwarnicki 2012, 2013; Mathis et al. 2016), herein, and in a forthcoming revision of the Neotropical species of *Discocerina* (Costa et al. in prep), we treat 38 species so far in seven genera from the fauna of Brazil alone. The purpose of this paper is to revise species of the genus *Lamproclasiopa*, including description of thirteen undescribed species.

This revision of *Lamproclasiopa* Hendel directly results from recent field work in southern Brazil that is part of an overall survey of the shore flies of this biologically diverse country. Although it is recognized that field and laboratory work are complimentary, even synergistic, the balance too often favors laboratory work. Herein we emphasize how extensive field work is a necessary and desirable compliment to comprehensive research at the desk level and eventually to publication. An objective of the field work in 2009-2010 and 2015 was the shore-fly fauna from the state of Paraná and to a lesser degree from Amazonas, Santa Catarina and São Paulo and resulted in numerous specimens of Discocerinini. Our sampling from Brazil (specimens recently collected and those from museums), however, is mostly from the southern states, and given this incomplete sampling, we anticipate additional species will yet be collected and eventually added to this diverse fauna. The same patchiness or in some cases the total lack of sampling applies to the Neotropical fauna in general. Responsible collecting of the Neotropical shore-fly fauna needs to be encouraged and to receive sustained support.

## Methods and materials

The descriptive terminology, with the exceptions noted in Mathis (1986) and Mathis and Zatwarnicki (1990a), follows McAlpine (1981). Because specimens are small, usually less than 2.60 mm in length, study and illustration of the male terminalia required use of a compound microscope. We have followed the terminology for most structures of the male terminalia that other workers in Ephydridae have used (references in Mathis 1986; Mathis and Zatwarnicki 1990a, 1990b), such as surstylus. Zatwarnicki (1996) suggested that the pre- and postsurstylus correspond with the pre- and postgonostylus and that the subepandrial sclerite is the same as the medandrium. The terminology for structures of the male terminalia is provided directly on Figs 3–6. We use the term basal flagellomere for the large antennomere beyond the pedicel. We prefer this term over “first flagellomere” as there may be more than one flagellomere involved, and basal does not imply a number or numbers. We likewise do not use “postpedicel” (Stuckenberg 1999) for this antennomere because at least the multisegmented arista is beyond the pedicel in addition to the large antennomere, and postpedicel is thus ambiguous and lacking precision.

Dissections of male terminalia were performed following Clausen and Cook (1971) and Grimaldi (1987). Abdomens were removed with microforceps and macerated in a sodium hydroxide solution. Cleared genitalia were then transferred to glycerin for observation, description, and illustration. The dissected abdomen was placed in a plastic microvial filled with glycerin and attached to the pin supporting the remainder of the insect from which it was removed. These structures for species of *Lamproclasiopa* are minute, and for accurate determinations using them, we often had to use a compound microscope to see them clearly.

The species descriptions are composite and not based solely on holotypes. One head and two venational ratios used in the descriptions are based on three specimens (largest, smallest, and one other): gena-to-eye ratio – genal height (immediately below maximum eye height)/eye height; costal vein ratio – the straight line distance between the apices of R_2+3_ and R_4+5_/distance between the apices of R_1_ and R_2+3_; M vein ratio – the straight line distance along vein M between crossveins dm-cu and r-m/distance apicad of dm-cu.

Distribution maps were made using ESRI ArcView GIS 3.2. Longitude and latitude coordinates were obtained for the locality where each specimen was collected and entered into a Microsoft Excel spreadsheet. If unavailable directly from specimen labels, longitude and latitude were estimated using gazetteers and maps to determine the geographical coordinates. Localities of specimens were plotted on a world land projection, presented within ESRI ArcView layouts and exported as encapsulated postscript (EPS) files.

The habitus illustrations are digital photographs taken with a Visionary Digital System. The images series obtained were combined by Zerene Stacker and Photoshop CS5 was used to adjust the color and make minor corrections (e.g., remove debris). Illustrations of male terminalia were made in Adobe Illustrator CS5.

Many specimens examined for this study are in the National Museum of Natural History, Smithsonian Institution, Washington, D.C. (USNM) and in the Universidade Federal do Paraná, Coleção Entomológica Padre Jesus Santiago Moure, Departamento de Zoologia, Curitiba, Paraná, Brazil (DZUP). We also borrowed and studied numerous specimens, especially primary types from the following museums:

AMNH
American Museum of Natural History, New York, New York (David A. Grimaldi) 


ANSP
Academy of Natural Sciences of Philadelphia, Pennsylvania (Jon K. Gelhaus and Jason D. Weintraub) 


BMNH
 The Natural History Museum (formerly the British Museum (Natural History)), London, England, United Kingdom (Kim Goodger) 


DEBU
 Department of Environmental Biology, University of Guelph, Guelph, Ontario, Canada (Stephen A. Marshall) 


INPA
Instituto Nacional de Pesquisas da Amazônia, Manaus, Amazonas, Brazil (Márcio Oliveira, José Albertino Rafael and Rosaly Ale-Rocha) 


IOC
 Instituto Oswaldo Cruz, Rio de Janeiro, Brazil (Jane Costa.) 


MNCR-A
 Colección de Entomología del Museo Nacional de Costa Rica, San José, Costa Rica (including collections of former INBio) (Silvia Lobo C.) 


MNRJ
 Museu Nacional do Rio de Janeiro, Rio de Janeiro, Brazil (Márcia Souto Couri) 


MZLU
Museum of Zoology, Lund University (Roy Danielsson) 


MZUSP
 Museu de Zoologia da Universidade de São Paulo, São Paulo, Brazil (Carlos José Einicker Lamas) 


NMW
Naturhistorisches Museum, Wien, Austria (Peter Sehnal) 


UMCE
 Instituto de Entomología, Universidad Metropolitana de Ciencias de la Educación, Santiago, Chile (Patricia Estrada M.) 


## Taxonomy

### Tribe Discocerinini

Taxon classificationAnimaliaDipteraEphydridae

Cresson

Discocerinini Cresson 1925a: 228 [as Discocerini]. Type genus: DiscocerinaMacquart 1835. Cresson 1942: 104 [correct spelling, as a “new tribe” in key]. Mathis and Zuyin 1989: 435 [diagnosis, monophyly]. Mathis and Zatwarnicki 1995: 163-186 [world catalog]. Zatwarnicki and Mathis 2001: 5-51 [tribal revision]. Zatwarnicki et al. 2016: 1-34 [phylogenetic review of tribe].

#### Diagnosis.

A tribe of Gymnomyzinae that is distinguished from other tribes of the subfamily by the following combination of characters:

*Head*: Frontal vitta (or ocellar triangle) mostly bare of setulae, not conspicuously setulose; ocellar setae well developed, inserted anterolaterad of anterior ocellus; reclinate fronto-orbital seta inserted anteromediad of proclinate fronto-orbital (if 2 proclinate fronto-orbital setae, reclinate seta inserted anteromediad of larger, posterior, proclinate seta); pseudopostocellar setae well developed, proclinate, slightly divergent, usually at least half length of ocellar setae. Pedicel bearing a large seta anterodorsally; arista bearing 4-6 dorsal rays, inserted along length of arista; conical process of basal flagellomere in lateral view finger-like. Face generally shallowly arched, frequently more prominent at level of dorsal facial setae, not conspicuously pitted, rugose, tuberculate, or carinate. Gena generally short (secondarily high in some species), bearing setulae (including midportion) and 1 large seta, its posterior (postgenal) margin rounded, not sharp. Oral opening and clypeus narrow; mouthparts generally dark colored; proboscis with number of pseudotracheae quite variable; lacinia Y-shaped with narrow posteromedial arm, dorsal arm spatulate; 2 different kinds of cibarium: (1) primitive type with dispersed medial sensillae arranged sparsely in a horizontal line; (2) advanced type with medial sensillae arranged densely in a sinuous line.

*Thorax*: Mesonotum generally microtomentose, frequently densely so, although variable; mesonotal setae weakly developed, only posteriormost pair of dorsocentral and acrostichal conspicuous; postsutural supra-alar seta usually evident although sometimes reduced or absent; prescutellar acrostichal setae inserted approximate and posterior of alignment of posteriormost dorsocentral setae; scutellar disc usually densely setulose; scutellum bearing 2 large, marginal setae: notopleural setae 2, inserted at same level near ventral margin, in some genera notopleuron bears setulae in addition to the two large notopleural setae (Figs 2, 9); anepisternum with 2 subequal setae inserted along posterior margin. Wing with vein R_2+3_ moderately long. Foreleg normally developed, not raptorial with greatly enlarged femur.

**Figures 1–2.**
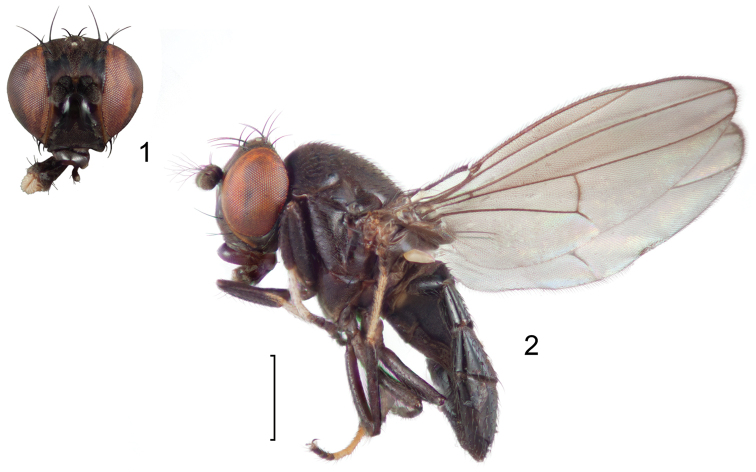
*Lamproclasiopa laevior* (Cresson). (Sri Lanka. N. E. District: Horton Plains) **1** head, anterior view **2** habitus, lateral view. Scale bar = 0.5 mm.

**Figures 3–6.**
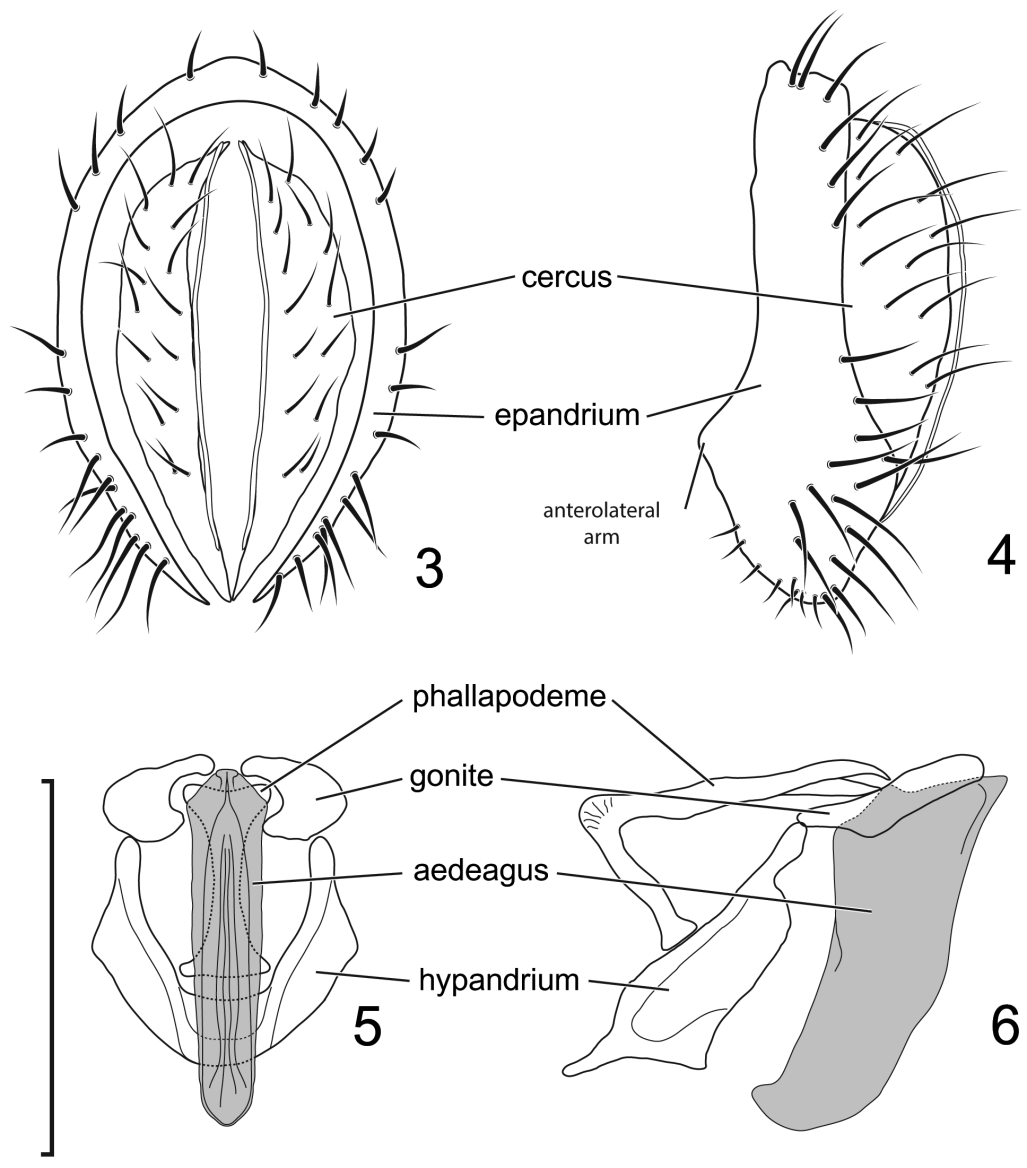
*Lamproclasiopa laevior* (Cresson). (Sri Lanka. N. E. District: Horton Plains) **3** epandrium and cerci, posterior view **4** same, lateral view **5** internal structures of male terminalia (aedeagus [shaded], phallapodeme, gonite, hypandrium), ventral view **6** same, lateral view. Scale bar = 0.1 mm.

*Abdomen*: Five tergites visible, usually not densely covered with microtomentum. Male terminalia: Epandrium as inverted U, encircling cerci, anterior margin rounded, in lateral view with setae mainly on dorsum and along anteroventral margin; cerci paired, hemispherical, setose; presurstylus lacking or fused indistinguishably with ventral margin of epandrium; anterolateral arms of epandrium attached with ventral apex of gonites, middle of posterior margin a base for phallapodeme; phallapodeme situated under aedeagus, associated with hypandrium and with ventral part of base of aedeagus, ventral margin with lobate appendix providing attachment for genital muscles that move aedeagus; gonite paired, connecting sides of base of aedeagus and laterodorsal margin of epandrium, bearing 1 or some setulae; aedeagus tubular, tapered anteriorly; ejaculatory apodeme usually lacking, if present as a spatula (Zatwarnicki et al. 2016, Figs 99–100).

#### Discussion.

Several of the characters noted in the diagnosis are synapomorphies and establish the tribe’s monophyly (Zatwarnicki et al. 2016). These are as follows: (1) ocellar setae inserted slightly in front of alignment of anterior ocellus; (2) reclinate fronto-orbital seta inserted in front of proclinate fronto-orbital seta; (3) conical process of basal flagellomere in lateral view finger-like; (4) prescutellar acrostichal setae small and inserted close together and behind the transverse alignment of the posteriormost dorsocentral setae (secondarily lacking in some species); and (5) presurstylus of the male terminalia either lacking or fused indistinguishably with the ventral margin of the epandrium. Larvae are microphagous and in other aspects are similar to those of Hyadinini (Ilytheinae).

As currently characterized, the tribe Discocerinini is one of the richest tribes within the family Ephydridae (225 species), and numerous additional species, especially from tropical zones, remain to be described. Many of the undescribed species are already in collections, and undoubtedly numerous others await collection. With the recent phylogenetic review of the tribe (Zatwarnicki et al. 2016) and description of additional genera and subgenera, there are now 13 genera and two subgenera. Two genera are monotypic and have relatively localized distributions: *Galaterina* in the Solomon and Andaman Islands and *Pectinifer* limited to the Neotropics (Mathis et al. 2016). Other genera are more speciose and widespread. *Aquachasma* (24 species), *Facitrichophora* (4 species), *Hydrochasma* (10 species), and *Polytrichophora* (nominate subgenus) (22 species) are found in the New World. The distributions of *Lamproclasiopa* (24 species) and *Orasiopa* (15 species) extend from the New World into the Australasian and Oriental Regions. *Diclasiopa* (4 species), *Gymnoclasiopa* (25 species), *Hecamedoides* (26 species) and *Ditrichophora* (39 species) have been recorded from all Regions except the Neotropics. Two genera, *Discocerina* (20 species) and *Polytrichophora* (subgenus *Sklodowskopa*) (10 species), are essentially cosmopolitan.

#### Phylogenetic considerations.

Zatwarnicki et al. (2016) proposed division of Discocerinini into four groups of genera (their proposed synapomorphies are provided in parentheses):

1. The *Gymnoclasiopa* group with *Gymnoclasiopa* (aedeagus with lateromedial appendices and facial setae arranged close to eye margin);
2. The *Diclasiopa* group with *Diclasiopa*, *Ditrichophora*, *Hecamedoides* and *Pectinifer* (gonite elongated, that is tapered apically);
3. The *Lamproclasiopa* group with *Galaterina*, *Lamproclasiopa*, and *Orasiopa* (subgenera *Orasiopa* and *Reymontopa*) (palpal setae with papilla-like bases); and
4. The *Discocerina* group with *Aquachasma*, *Discocerina*, *Facitrichophora*, *Hydrochasma* and *Polytrichophora* (nominate subgenus and subgenus *Sklodowskopa*) (reduced number of pseudotracheae, modified cibarium and the ventral receptacle bearing anterodorsal projection).

Zatwarnicki et al. (2016) acknowledged that groups three and four together (*Aquachasma*, *Discocerina*, *Galaterina*, *Hydrochasma*, *Lamproclasiopa*, *Orasiopa*, and *Polytrichophora*) form a clade that is the best supported lineage within the tribe, being based on (1) notopleuron setulose and (2) gonites elongated and bar-like without an anterior projection or the gonite is fused with the hypandrium (character 32.1-2). As such, we prefer the continued recognition of these seven genera as a single group, the *Discocerina* group, and use subgroups for further division of this group (the *Lamproclasiopa* and the *Discocerina* groups of Zatwarnicki et al. 2016).

In the classification that Zatwarnicki et al. (2016) proposed (their character numbers are in parentheses), the *Lamproclasiopa*-subgroup has palpal setae with papilla-like bases (character 13). Within the *Lamproclasiopa* subgroup, the monophyly of the genus *Lamproclasiopa* is established by two characters (autapomorphies): (1) postsutural supra-alar (character 11 in Zatwarnicki et al. 2016) and (2) prescutellar acrostichal setae greatly reduced or lacking (character 22 in Zatwarnicki et al. 2016). The monophyly of its sister group, the combined *Galaterina* + *Orasiopa*, is confirmed by an increased number of pseudotracheae (convergent with *Pectinifer*). Thus, in the most recent classification, *Lamproclasiopa* is the sister-group of the combined lineage of *Galaterina* + *Orasiopa*, and these three genera together form an assemblage that is now the *Lamproclasiopa* subgroup.

#### Key to genera of Discocerinini

**Table:** 

1	Notopleuron bare of setulae	**2**
–	Notopleuron setulose in addition to 2 large setae	**6**
2	Forefemur bearing distinct row of stout, short setae along apical half of posteroventral surface	**3**
–	Forefemur lacking row of short, stout setae along posteroventral surface	**4**
3	Face metallic shiny bearing white microtomentose spots laterally; forefemur slightly enlarged	***Pectinifer* Cresson**
–	Whole face shiny or completely covered with microtomentum; forefemur normally developed	***Ditrichophora* Cresson**
4	Postsutural supra-alar seta strong, distinct, longer than posterior notopleural seta. Face with dorsoclinate seta at lower lateral extremity	***Diclasiopa* Hendel**
–	Postsutural supra-alar seta very short or absent, if distinguishable distinctly shorter than posterior notopleural seta. Face without dorsoclinate seta at lower lateral extremity	**5**
5	Hindtibia with a preapical, ventral, spur-like seta; face rather prominent at level of dorsal facial setae, sometimes transversely carinate; facial series of setae inserted in some distance to parafacial, comprising 2-3 large setae; dorsal seta inserted slightly medially from other setae and arising from distinct, shiny papilla, with a small, slightly dorsoclinate seta laterad of dorsal seta	***Hecamedoides* Hendel**
–	Hindtibia lacking a preapical, ventral spur-like seta; face rather flattened, antennal grooves not always sharply defined ventrally; facial series of setae inserted very close to parafacial, comprised of 2 large setae; dorsal seta not arising from a shiny papilla and lacking a smaller seta laterad of dorsal seta	***Gymnoclasiopa* Hendel**
6	Mesonotum bearing numerous, long setulae	***Galaterina* Zatwarnicki & Mathis**
–	Mesonotum lacking numerous, long setulae	7
7	Face with 2 or more conspicuous rows of setae/setulae on each side, parallel to facial suture setal row medial, with a row(s) of setulae between setal row and parafacial	**8**
–	Face with a single row of setae laterally	**10**
8	Face with setae and setulae of rows inclinate or ventroinclinate	***Facitrichophora* Mathis & Zatwarnicki**
–	Face with secondary series of dorsolaterally inclined setae laterad to primary series (*Polytrichophora* Cresson)	**9**
9	Parafacials becoming 3-4 times wider ventrally; gena high, at least 1/4 eye height	subgenus ***Polytrichophora* Cresson**
–	Parafacials 2-3 times wider ventrally; gena narrow, less than 1/4 eye height	subgenus ***Sklodowskopa* Zatwarnicki**
10	Gena and lower part of parafacial broad; lateral margin of abdomen usually with gray to whitish microtomentose areas, these usually wedge shaped	**11**
–	Gena and parafacial rather narrow; abdomen lacking wedge-shaped, light-colored areas laterally	**12**
11	Head subglobose, oral opening comparatively large; dorsum of tergites darker dorsomedially than on lateral margins, but without contrasting areas	***Hydrochasma* Hendel**
–	Head not subglobose, oral opening comparatively small; dorsum of tergites 2-4 extensively dark gray to black with sharply contrasted gray lateral margin or with wedge-shaped silvery-gray areas	***Aquachasma* Zatwarnicki**
12	Parafacial bearing setulae	***Discocerina* Macquart**
–	Parafacial lacking setulae	**13**
13	Facial series of setae 2, these well separated, distance between subequal to length of basal flagellomere; parafacial very narrow at anteroventral margin of eye; postsutural supra-alar and prescutellar acrostichal setae greatly reduced or lacking	***Lamproclasiopa* Hendel**
–	Facial series of setae 3-4, distance between setae conspicuously less than length of basal flagellomere; parafacial evenly wide throughout length; postsutural supra-alar and prescutellar acrostichal setae present (*Orasiopa* Zatwarnicki & Mathis)	**14**
14	Species slender; antenna largely yellow; arista bearing 5 dorsal rays; palpus yellow; knob of halter dark; thorax and abdomen gray microtomentose; legs mostly yellow (sometimes midfemur dark)	subgenus ***Orasiopa* Zatwarnicki & Mathis**
–	Species compact; antenna dark brown or black; arista bearing usually 7–11 dorsal rays; palpus brownish or black; knob of halter white; coloration of thorax and abdomen dark brown or black; legs mostly dark brown to black	subgenus ***Reymontopa* Zatwarnicki**

### Genus Lamproclasiopa

Taxon classificationAnimaliaDipteraEphydridae

Hendel

Lamproclasiopa Hendel 1933: 79 [as a subgenus of Discocerina]. Type species: Lamproclasiopa facialisHendel 1933, original designation. Zatwarnicki and Mathis 2001: 36 [status as a genus; generic diagnosis]. Wirth 1968: 7 [Neotropical catalog]. Mathis and Zatwarnicki 1995: 168-169 [world catalog]. Zatwarnicki et al. 2016: 16-19 [recharacterization in phylogenetic review of tribe].
Basila Cresson 1942: 116. Type species: Ditrichophora nadinaeCresson 1925a, original designation. Zatwarnicki and Mathis 2001: 36 [synonymy].

#### Diagnosis.

*Lamproclasiopa* is distinguished from other genera of Discocerinini by the following combination of characters: Small to medium-sized shore flies, body length 1.7–3.0 mm; generally sparsely to densely microtomentose, subshiny to dull species (Figs 2, 9, 25, 57, 69). *Head*: One proclinate and one reclinate pair of fronto-orbital setae. Arista usually bearing 5 dorsal rays, rarely 6. Face moderately prominent at level of dorsal facial seta; antennal grooves generally distinctly defined ventrally; face lacking secondary series of setae; facial setae 2, dorsal setae not arising from shiny papilla, lacking a dorsoclinate seta at lower lateral extremity; parafacial narrow to moderately wide throughout length, lacking ventroclinate setulae; gena generally short but very high in the *polita* group. Eye generally oval, moderately microsetulose, bearing interfacetal setulae (sometimes not discernible by light stereomicroscope). Proboscis with 7 pseudotracheae; cibarium of primitive type with 4 medial sensillae arranged in a horizontal row and 4 moderate posterior sensillae. *Thorax*: Anterior notopleural seta inserted near middle toward ventral margin, distance between anterior and posterior setae slightly less than half distance between postpronotal seta and anterior notopleural seta; notopleuron bearing several setulae in addition to 2 larger setae; presutural supra-alar seta usually present, well developed; postsutural supra-alar seta lacking; acrostichal setae, including prescutellar pair, lacking, only tiny setulae present. Wing variable, mostly to completely hyaline in most species but some with maculation pattern; costa bearing 5–6 long, dorsal setae between humeral and subcostal breaks; costal vein ratio varying between 0.40–0.90. Forefemur normally developed, lacking row of short, stout setae along posteroventral surface; hindtibia lacking a preapical, ventral, spur-like seta. Stem of halter blackish brown, knob white to whitish yellow. *Abdomen*: Tergites usually unicolorous, lacking pale-colored areas laterally; male tergite 4 longer than tergite 3. Male terminalia: Epandrium as inverted U in posterior view, dorsal arch complete; arms separate ventrally beyond cerci, surface covered with setae; cercus not fused with epandrium, in posterior view semicircular or crescent-shaped; gonites variously shaped, usually symmetrical, separate from hypandrium, in lateral view generally lunate without setulae; aedeagus longer than wide, mostly tubular, in ventral view navicular, without projections, in lateral view cigar-shaped or tapered toward apex; phallapodeme separate from aedeagus, in ventral view variously shaped; in lateral view irregularly triangular with distinct ventral projection; hypandrium in ventral view U- or Y-shaped with long posterolateral arms (incision reachs to 1/3–1/2 hypandrial length, in lateral view flat, sometimes slightly arched; ejaculatory apodeme absent. Female terminalia: Ventral receptacle without operculum, C-shaped stalk with broader head.

#### Distribution

(Figs 7, 14, 36, 59, 81, 104, 111, 139). Oriental, Nearctic and Neotropical Regions.

#### Discussion.

With the exception of *Lamproclasiopa laevior* (Cresson), which is a very disjunct species, occurring only on the Indian Subcontinent, the other congeners are found thus far only in the New World and there primarily in the Neotropics. In the older literature, including catalogs, this genus was frequently treated as a subgenus of *Discocerina* (see generic and species’ synonymies).

We have arranged all recognized species into species groups based primarily on similarity, both external features and structures of the male terminalia. These groups are not necessarily monophyletic, although some are. Within a species group, the species are treated in alphabetical order.

#### Key to species of *Lamproclasiopa*

**Table:** 

1	Gena high to very high (gena-to-eye ratio between 0.30–0.64) and with an acutely sharp genal/postgenal margin (Figs 26, 38)	**2**
–	Gena relatively short (gena-to-eye ratio less than 0.20) and with genal/postgenal margin rounded	**4**
2	Frons nearly bare, shiny (Figs 37, 38). Distal 3 tarsomeres black; male tergite 5 narrowly rounded posteriorly	***Lamproclasiopa polita* (Edwards)**
–	Frons with anterior half densely microtomentose (Fig. 24). Distal 2–3 tarsomeres yellow; male tergite 5 truncate posteriorly	**3**
3	Female frons with broad, transverse stripe on anterior half; male mesonotum with microtomentum on anterior third	***Lamproclasiopa lapaz* sp. n.**
–	Female frons mostly bare, shiny, at most with an anteromedial spot and at base of ocellar setae and on some parts of ocellar triangle (Fig. 25); male mesonotum with broad stripe of microtomentum, stronger anteriorly, becoming weaker posterior (Fig. 24)	***Lamproclasiopa auritunica* sp. n.**
4	Wing maculate, at least over crossveins or generally conspicuously infuscation (Figs 58, 62, 70)	**5**
–	Wing generally hyaline, lacking a maculation pattern or general infuscate	**8**
5	Wing generally infuscate with blackish veins and crossveins (Fig. 132)	***Lamproclasiopa fumipennis* (Wirth)**
–	Wing with pattern of spots or with a spot over crossveins but not generally infuscate	**6**
6	Only crossveins r-m and dm-cu with darkened cloud; vein R_2+3_ curved gently apically, not angulate subapically nor bearing a subapical stump vein (Fig. 62)	***Lamproclasiopa mancha* sp. n.**
–	Wing with numerous dark spots; vein R_2+3_ angulate subapically and bearing a stump vein with a posteroapical orientation, a second stump vein near middle (Figs 58, 70)	**7**
7	Mesonotum with 4 brown interrupted vittae, each with elongate, mostly separate spots, none in acrostichal row (Fig. 69)	***Lamproclasiopa painteri* (Cresson)**
–	Mesonotum with 7 brown, mostly entire vittae, including a medial vitta in acrostichal area (Fig. 57)	***Lamproclasiopa balsamae* (Cresson)**
8	Head, thorax, and abdomen generally shiny black, only tarsi and antennal grooves yellow or cinereous (Figs 1–2, 8–9, 98–99)	**9**
–	Body with extensive surfaces sparsely to densely microtomentose	**11**
9	Face completely shiny black (Fig. 8)	***Lamproclasiopa brunnea* sp. n.**
–	Face microtomentose, silver white to golden	**10**
10	Frons generally shiny black; coxae blackish brown	***Lamproclasiopa hendeli* (Wirth)**
–	Frons microtomentose, silvery white or golden; forecoxae light gray (Fig. 98–99)	***Lamproclasiopa argentipicta* sp. n.**
11	Forebasitarsomere white, contrasted with black apical tarsomeres (Fig. 2)	***Lamproclasiopa laevior* (Cresson)**
–	Forebasitarsomere yellow to slightly blackish yellow, not distinctly contrasted with coloration of apical tarsomeres	**12**
12	Gena relatively high, height subequal to height of basal flagellomere	**13**
–	Gena relatively short, height about ½ height of basal flagellomere	**18**
13	Presutural supra-alar seta lacking; katepisternum, especially anterior half, and anteroventral portion of anepisternum shiny black; forefemur with 4–5 stout, peg-like setae on apical third along posteroventral margin	**14**
–	Presutural supra-alar seta well developed; katepisternum and anepisternum thinly microtomentose, generally appearing dull, not shiny; forefemur with posteroventral setae slender, not stout and peg-like	**15**
14	Pocket between epandrial arms of male uniformly U-shaped	***Lamproclasiopa nadineae* (Cresson)**
–	Pocket between epandrial arms of male bottle-shaped, with basal half as a narrower neck and apical half wider (Fig. 94)	***Lamproclasiopa aliceae* sp. n.**
15	Eyes covered with tiny, dense setulae; facial microtomentum gray; frons concolorous with mesonotum	**16**
–	Eyes covered with very sparse setulae or bare; facial microtomentum gray or dark gray; anterior portion of frons usually yellowish orange to some degree	**17**
16	Epandrium higher than wide; aedeagus thin, narrowly funnel-like, straight (Figs 135–138)	***Lamproclasiopa puella* (Cresson)**
–	Epandrium as high as wide; aedeagus wide, with apex acutely pointed and curved (Figs 128–131)	***Lamproclasiopa caligosa* sp. n.**
17	Aedeagus thin, narrowly funnel-like, straight; gonites without laterodorsal extensions (Fig. 116–117)	***Lamproclasiopa aracataca* (Cresson)**
–	Aedeagus wider, curved laterally; gonites with laterodorsal extensions (Fig. 130–131)	***Lamproclasiopa curva* sp. n.**
18	Antenna yellow (Figs 75, 86)	**19**
–	Antenna extensively darkened dorsally, only basoventral portion of basal flagellomere and pedicel partially orange to yellowish	**21**
19	Tibiae entirely black; presutural supra-alar seta well developed; frons and face distinctly two-toned	***Lamproclasiopa nana* (Williston)**
–	Tibiae partially or entirely yellow; frons and face generally unicolorous; presutural supra-alar lacking	**20**
20	Tibiae black brown with the distal third yellow; frons with 2 small shiny black areas lateroanteriorly	***Lamproclasiopa furvitibia* sp. n.**
–	Tibiae entirely yellow; frons without shiny black areas (Figs 86–87)	***Lamproclasiopa xanthocera* sp. n.**
21	Face with a mediovertical, narrowly triangular, sparsely microtomentose spot (Fig. 18)	***Lamproclasiopa triangularis* sp. n.**
–	Face mostly shiny black, especially medial portion, this area lacking a microtomentose, triangular pattern	**22**
22.	Foretarsus blackish yellow, apical tarsomeres becoming darker	***Lamproclasiopa zerafael* sp. n.**
–	Foretarsus yellow, apical tarsomeres yellow or becoming darker	**23**
23	Foretarsus yellowish, apical 1-2 tarsomeres darkened	***Lamproclasiopa bisetulosa* (Cresson)**
–	Foretarsus completely yellow	***Lamproclasiopa ecuadoriensis* sp. n.**

### The *laevior* group (*Lamproclasiopa laevior*)

**Diagnosis.** Body generally subshiny to shiny black. *Head*: Frons and face generally unicolorous; gena moderately high (gena-to-eye ratio 0.16-0.22); genal/postgenal margin rounded. *Thorax*: Presutural supra-alar seta well developed; pleural areas generally shiny black. Wing hyaline to very faintly infuscate, lacking pattern of spots; vein R_4+5_ extended gradually toward costa, curved gently subapically, not angulate or bearing a stump vein. Femora and tibiae black; foretarsus with basal 2 tarsomeres white, tarsomere 3 darkened, apical 2 tarsomeres black; mid- and hindtarsus with basal 2 tarsomeres yellow, apical 3 tarsomeres blackish; forefemur lacking 4-5 stout, peg-like setae on apical third along posteroventral margin. *Abdomen*: Male terminalia: Epandrial sides in posterior view uniformly thin, thinner than width of cerci; cerci almost same height as epandrium; hypandrium generally U-shaped, narrow, without a wide projection ventrally.

**Remarks.** For the present, *Lamproclasiopa laevior* is the only included species in this species group, and among all known congeners, this is the only species known to occur only in the Old World. Its anomalous and disjunct distribution (see “Remarks” for this species) is perhaps partially explained by the proposed sister group of *Lamproclasiopa*, which is the combined lineage of *Galaterina + Orasiopa* (Zatwarnicki et al. 2016). The latter two genera are found primarily in the Old World (*Orasiopa mera* (Cresson) occurs also in the New World, probably as an introduction), and there are many species of *Orasiopa* that occur in the Oriental Region (Mathis and Zatwarnicki 1995). Thus far, however, no species of either *Galaterina* or *Orasiopa* are known from the Indian Subcontinent. We suggest that this may also well represent sampling error rather than actual distributions of all included species whether described or not. The shore-fly fauna of the Oriental Region has not been well sampled.

#### Lamproclasiopa laevior

Taxon classificationAnimaliaDipteraEphydridae

(Cresson)

Figs 1–2 , 3–6 , 7

Ditrichophora laevior Cresson 1934: 200 [India. Darjeeling, Behar; HT ♂, ANSP (6509)].
Discocerina (Lamproclasiopa) laevior . Cresson 1945: 59 [generic combination]. Mathis and Zatwarnicki 1995: 168 [world catalog].
Lamproclasiopa laevior . Zatwarnicki and Mathis 2001: 39 [generic combination].

##### Diagnosis.

This species is distinguished from other congeners by the following combination of characters: Small to moderately small shore-flies, body length 1.85–2.65 mm, generally black, subshiny to shiny species. *Head*: Frons black, very sparsely microtomentose, anterior half shiny, posterior subshiny, transition from shiny to subshiny gradual. Antenna black, densely microtomentose, appearing velvety black. Face black, sparsely microtomentose, antennal grooves and lateral areas shiny, otherwise subshiny, lacking prominent, vertical stripes; face bearing 2–3 larger facial setae, dorsal seta at about midfacial height, dorsomesoclinate; ventral seta just dorsad of epistomal margin, slightly dorsoclinate; parafacial blackish yellow; gena moderately high, gena-to-eye ratio 0.16–0.22. *Thorax*: Mesonotum uniformly sparsely microtomentose, black, subshiny; presutural supra-alar seta well developed; pleural area concolorous with mesonotum, subshiny black. Wing hyaline to very faintly infuscate, lacking pattern of spots; vein R_4+5_ extended at gradual to costa, not angulate subapically or bearing a stump vein; costal vein ratio 0.47–0.55; M vein ratio 0.60–0.62. Femora and tibiae black; Forefemur with posteroventral setae slender, not stout and peg-like; foretarsus with basal 2 tarsomeres white, tarsomere 3 darkened, apical 2 black; mid- and hindtarsus with basal 2 tarsomeres yellow, apical 3 tarsomeres blackish. *Abdomen*: Generally black, mostly subshiny to shiny, dorsum of tergites very sparsely and finely microtomentose. Male terminalia (Figs 3–6): Epandrium in posterior view (Fig. 3) generally vertically oval, each lateral arm narrow, almost parallel sided, acutely pointed ventrally, in lateral view (Fig. 4) with dorsal 2/3 rectangular, basal 1/3 almost twice width as dorsal portion, widest subventrally, apex broadly rounded, posteroventral portion bearing several larger setulae; cerci in posterior view (Fig. 3) elongate, narrowly semicircular, medial margin nearly straight, gradually tapered toward ventral apex, this apex acutely pointed, dorsal apex with medial short, digitiform extension, in lateral view (Fig. 4) semicircular; gonite in lateral view (Fig. 6) narrowly elongate, somewhat rod-like, ventral 1/3 narrower than dorsal 2/3, in ventral view (Fig. 5) very robustly C-shaped with medial concavity, wider than high; aedeagus in lateral view (Fig. 6) tubular, gradually tapered to apex, apex generally broadly rounded with very apex narrowed, curved anteriorly at nearly right angle; phallapodeme in lateral view (Fig. 6) very narrow, L-shaped, apex toward base of aedeagus acutely pointed, apex toward hypandrium slightly flared and truncate, in ventral view (Fig. 5) as an elongate hourglass, expanded at each apex and truncate; hypandrium in lateral view (Fig. 6) narrowed posteriorly, then abruptly expanded to widest point subanteriorly, anterior margin abruptly narrowed, digitiform, in ventral view (Fig. 5) generally U-shaped, lateral arms widest at midlength, anterior margin broadly rounded, deep, posterior emargination narrowed on anterior 1/3, thereafter posterior more than twice anterior width.

##### Type material.

The holotype male of *Ditrichophora laevior* Cresson is labeled “Darjeeling Fruhstorfer/9233/TYPE Ditrichophora LAEVIOR E. T. Cresson, Jr. [maroon-red; “Ditrichophora LAEVIOR” handwritten].” The holotype is double mounted (minuten pin in a rectangular block of foam), is in good condition (abdomen removed, dissected, and in an attached microvial), and is deposited in the ANSP (6509). A female paratype (ANSP) bears the same locality label as the holotype.

##### Type locality.

India. West Bengal: Darjeeling, Cooch Behar (26°24.7'N, 89°23.1'E). The holotype was apparently collected in the foothills of the Himalayas in the state of West Bengal between Darjeeling and Cooch Behar.

##### Other specimens examined.

INDIA. Meghalaya: Shillong (Botanical Gardens; 25°34.6'N, 91°53.2'E), 20 Apr 1980, A Freidberg (9♂, 2♀; USNM).

SRI LANKA. Central Province: Horton Plains (6°48.7'N, 80°47.3'E), 23 Apr 1980, W. Mathis, T. Wijesinhe & L. Jayawickrema (9♂, 4♀; USNM).

##### Distribution

**Figure 7.**
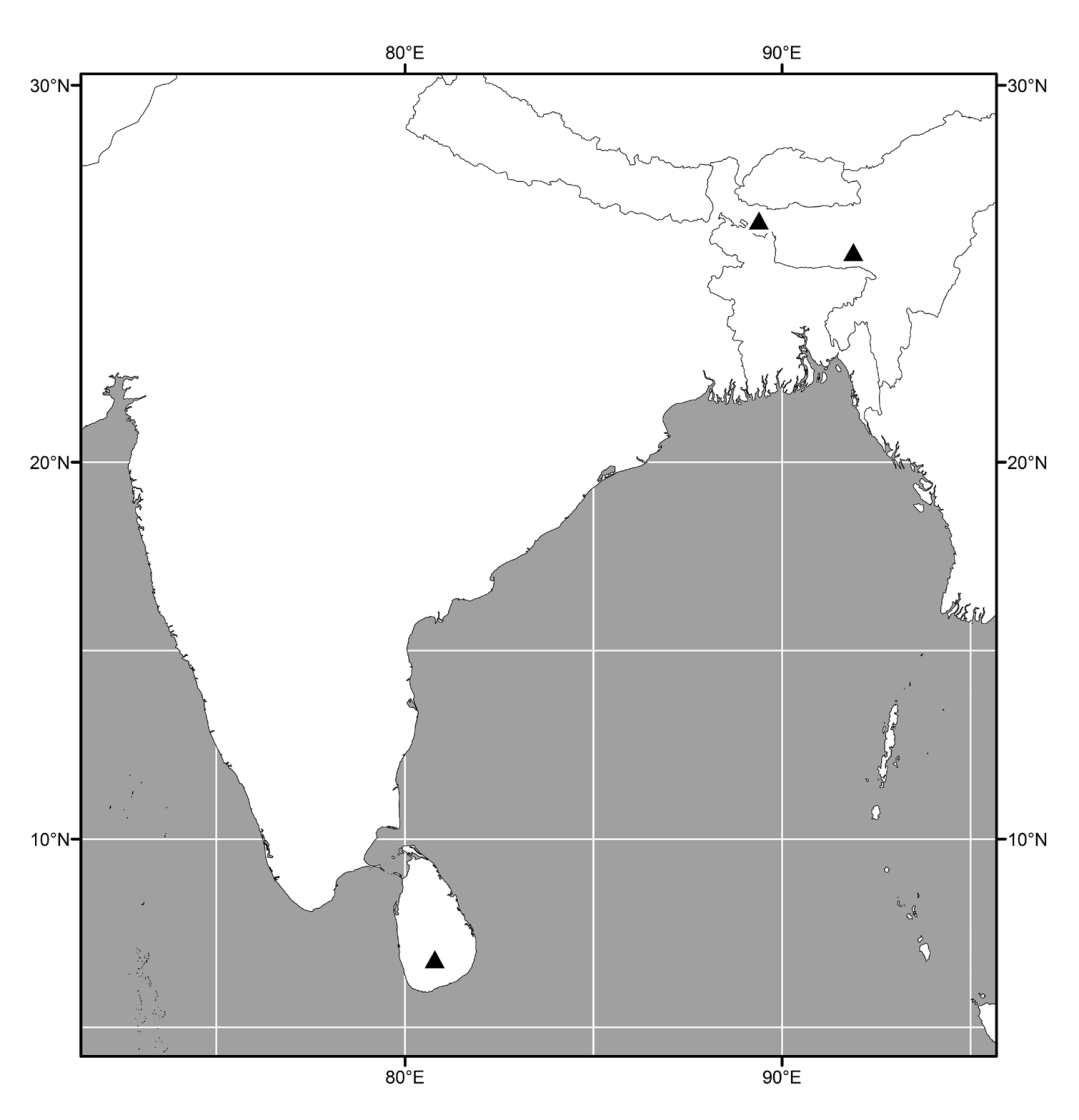
Distribution map of *Lamproclasiopa laevior* (▲).

(Fig. 7). Oriental: India (Meghalaya, West Bengal), Sri Lanka.

##### Remarks.

Although similar and perhaps related to *Lamproclasiopa ecuadoriensis*, this species is distinguished from congeners by the white forebasitarsomere, which is contrasted with the black, apical tarsomeres; the short gena (gena-to-eye ratio 0.10–0.14); and the shape of structures of the male terminalia (Figs 3–6).

The distribution of this species is anomalously disjunct. Except for this species, which is found on the Indian Subcontinent, all other congeners occur in the New World, especially in tropical zones, which are many thousands of kilometers from India and Sri Lanka. This distributional anomaly prompts a number of questions. For example, is this species indeed a valid congener? If so, what is it related to? How did it come to be found on the Indian Subcontinent when other congeners occur in the New World? Although our responses to these and other questions are not wholly satisfactory, often being based on tenuous evidence, we offer some thoughts and observations.

Based on morphological evidence, both external and from structures of the male terminalia, we affirm that this is a congener within the genus *Lamproclasiopa*. Within *Lamproclasiopa*, we suggest that this species, being generally characterized by homoplasious characters, would probably be near the base of the evolutionary stem or node giving rise to all other included congeners. See also our remarks under the species group.

As we noted in the introduction, however, characterization of *Lamproclasiopa* has only become reasonably well resolved in recent decades, resulting in earlier recognized species, including this species, being first described in another genus. Cresson (1934) first described this species in *Ditrichophora* then transferred it to the subgenus *Lamproclasiopa* within *Discocerina* (Cresson 1945). Over 50 years later, Zatwarnicki and Mathis (2001) accorded generic status to *Lamproclasiopa* and included this species along with other congeners in this genus.

### The *hendeli* group (*Lamproclasiopa brunnea*, *Lamproclasiopa hendeli*)

**Diagnosis.** Body generally subshiny to shiny black, only tarsi and antennal grooves yellow or cinereous. *Head*: Frons and face generally unicolorous; frons sparsely microtomentose; genal height variable, moderately high to high (gena-to-eye ratio 0.12–0.25); genal/postgenal margin rounded. *Thorax*: Presutural supra-alar seta lacking or indistinguishable from surrounding setae; katepisternum and anepisternum thinly microtomentose, generally appearing dull, not shiny. Wing generally hyaline to very faintly infumate; vein R_2+3_ curved gently apically, not angulate subapically nor bearing a subapical stump vein. Forefemur with posteroventral setae slender, not stout and peg-like; tarsi yellowish. *Abdomen*: Male terminalia: Cerci around 2/5 height of epandrium; hypandrium generally wide, with arms long or short dorsally, not U-shaped.

**Remarks.** This species group is mostly based on homoplasious characters, and we cannot confirm its monophyly. The two included species are similar to each other and the species group can be diagnosed. These are the bases for recognition of this species group.

#### Lamproclasiopa brunnea sp. n.

Taxon classificationAnimaliaDipteraEphydridae

http://www.zoobank.org/2875BDA6-0054-4F85-AC3A-8B75173BBA37

**Figures 8–9.**
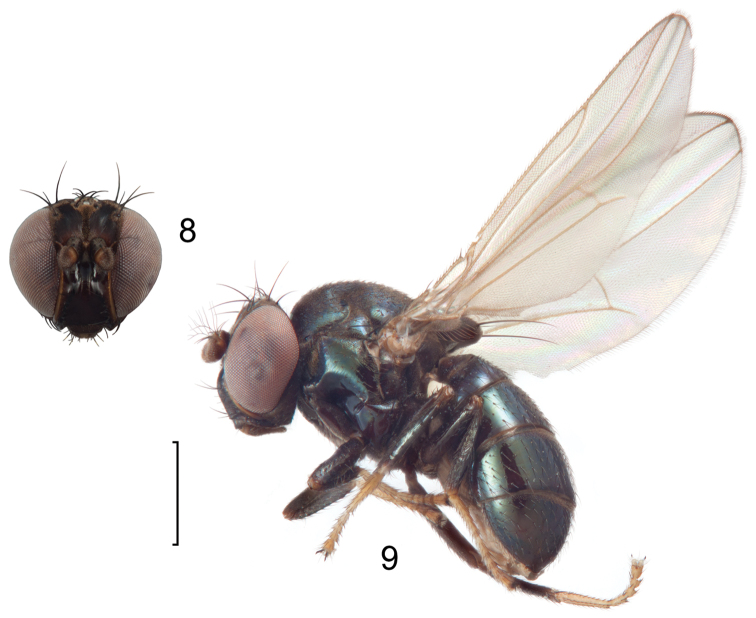
*Lamproclasiopa brunnea* sp. n., male paratype (Costa Rica. San José. Moravia) **8** head, anterior view **9** habitus, lateral view. Scale bar = 0.5 mm.

**Figures 10–13.**
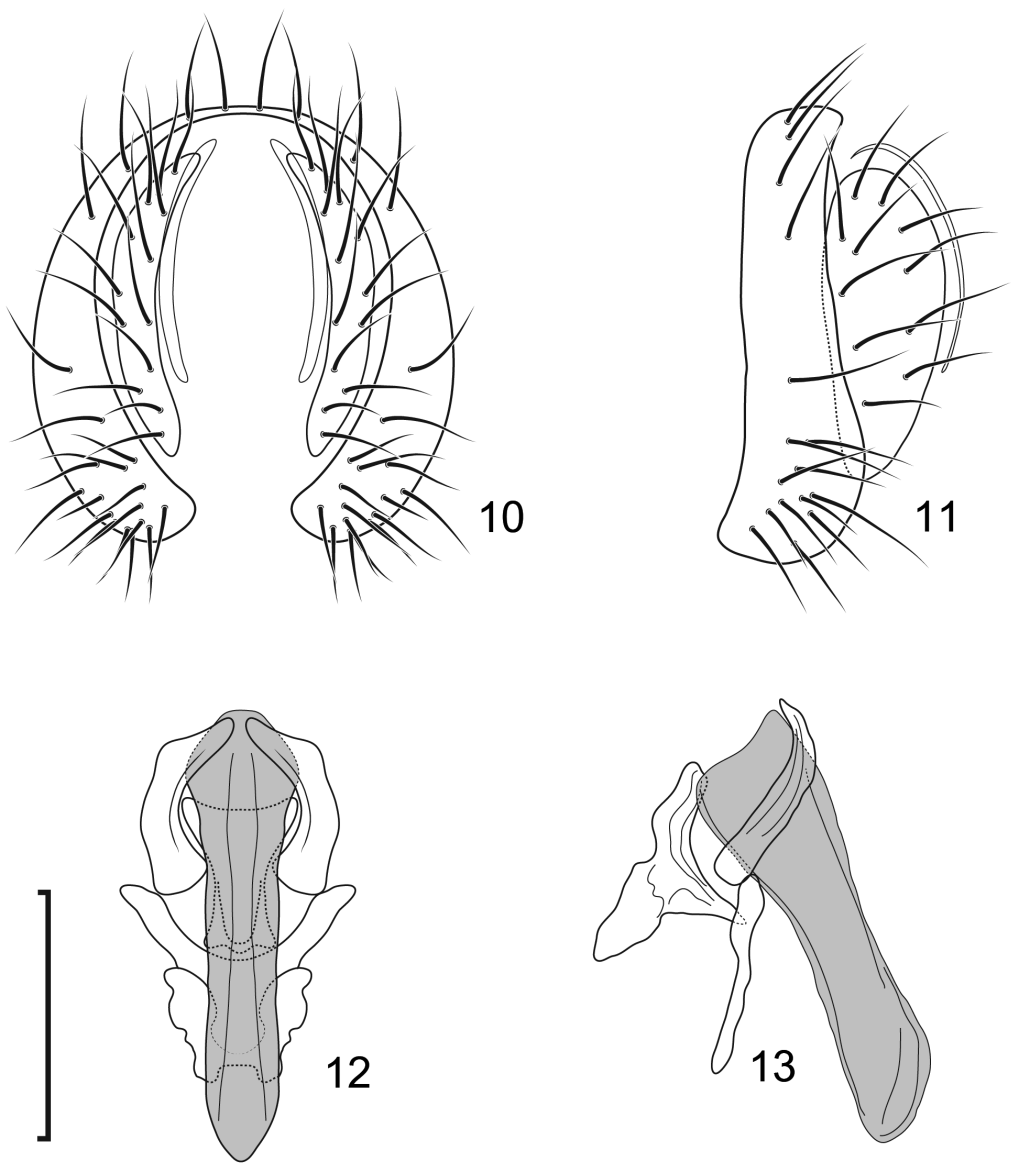
*Lamproclasiopa brunnea* sp. n., male paratype (Costa Rica. San José. Moravia) **10** epandrium and cerci, posterior view **11** same, lateral view **12** internal structures of male terminalia (aedeagus [shaded], phallapodeme, gonite, hypandrium), ventral view **13** same, lateral view. Scale bar = 0.1 mm.

**Figure 14.**
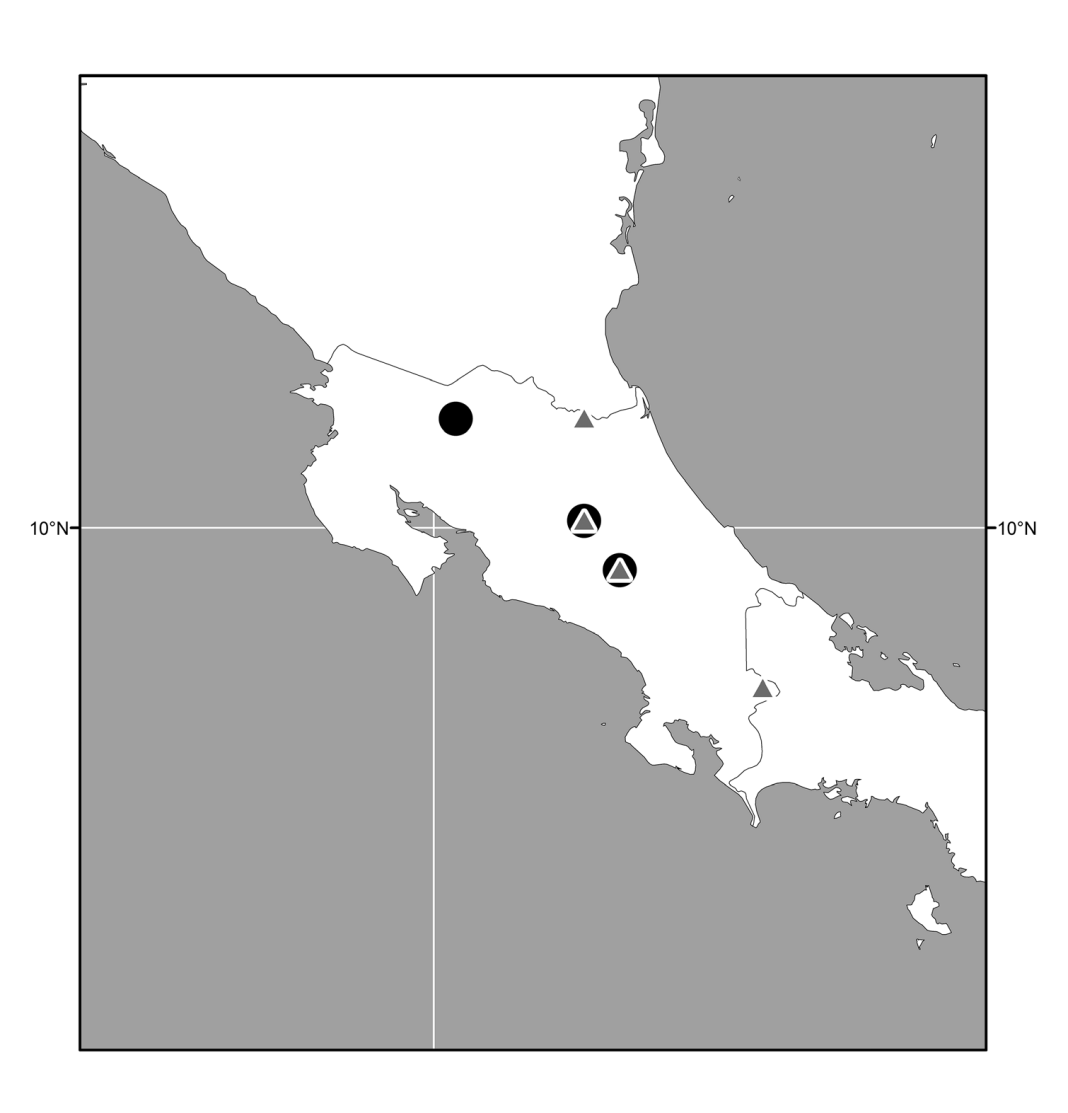
Distribution map of *Lamproclasiopa brunnea* sp. n. (●); *Lamproclasiopa furvitibia* sp. n. (▲).

Figs 8–9 , 10–13 , 14

##### Diagnosis.

This species is distinguished from congeners by the following combination of characters: Moderately small shore flies, body length 2.60 mm. *Head*: Frons shiny black brown, with ocellar triangle and fronto-orbital plate densely setulose. Antenna blackish brown, slightly lighter than head, posterior margin of flagellomere light brown; face blackish brown, shiny; parafacial yellowish brown. Gena moderately high, gena-to-eye ratio 0.12–0.15. *Thorax*: Mesonotum shiny black, covered with microtomentum; presutural supra-alar seta lacking or indistinguishable from surrounding setae; pleural region less microtomentose, anepisternum and katepisternum almost bare, shiny black. Wing hyaline, lacking any pattern or markings; costal vein ratio 0.52–0.64; M vein ratio 0.59–0.65; Legs blackish brown except yellowish tarsi; forefemur with posteroventral setae slender, not stout and peg-like. *Abdomen*: Generally shiny black, bare of microtomentum; tergites 3–5 equal in length and larger than tergites 1–2. Male terminalia (Figs 10–13): Epandrium in posterior view (Fig. 10) generally oval, higher than wide, dorsal portion very thin, each lateral arm gradually becoming wider ventrally, widest on apical third, apex rounded, oriented medially, ventral half with slightly increased number of setulae, in lateral view (Fig. 11) as roughly rectangular, slightly wider ventrally with rounded posteroventral portion, pointed anteroventrally; cerci in posterior view (Fig. 10) elongate, thin, generally shallowly arched, ventral and dorsal apices acutely pointed, setulose evenly along length, in lateral view (Fig. 11) as an inverted drop; gonite in lateral view (Fig. 13) rod-like, dorsal fourth curved basally and pointed, in ventral view (Fig. 12) irregularly bar-like, curved, apex toward aedeagal base tapered, pointed apically, apex toward hypandrium bluntly rounded; aedeagus in lateral view (Fig. 13) more or less rectangular, basal third wider than apical portion, narrowest medially, apex angulate, in ventral view (Fig. 12) elongate, thin, after slightly bulbous base parallel sided, apex tapered, pointed; phallapodeme in lateral view (Fig. 13) as a dissected triangle, with an obvious keel, vertex toward hypandrium sharply tapered, acutely pointed, vertex toward aedeagal base thumb-like, keel narrow, moderately elongate, pointed, in ventral view (Fig. 12) as an asymmetrical spool, wider toward aedeagal base, apex toward hypandrium shallowly trilobed; hypandrium in lateral view (Fig. 13) elongate, thin, irregularly rod-like, shallowly sinuous, both apices narrowly rounded; in ventral view (Fig. 12) robustly Y-shaped, with base wide, lateral margins almost serrate, arms of Y posterior, flared posterolateral, each arm thin, digitiform, forming posterior, evenly rounded, moderately deep emargination.

##### Type material.

The holotype male of *Lamproclasiopa brunnea* is labeled “COSTA RICA. Prov. San José. Moravia. Zurquí de Moravia, Tower path. 1600m. 2–9 AGO 2013. Proyeto ZADBI. Mix methods, ZADBI-1076/HOLOTYPE ♂ *Lamproclasiopa brunnea* Costa, Mathis & Marinoni USNM [red].” The holotype is double mounted (glued to a paper triangle) and is in very good condition, and is deposited in MNCR-A. Thirty-eight paratypes (20♂, 18♀; MNCR-A, USNM) bear the same label data as the holotype. Other paratypes are as follows: COSTA RICA. **Cartago.** Paraíso, Parque Nacional Tapantí (09°43.3'N, 83°46.5'W; 1600 m), 4–11 Ago 2013, Proyeto ZADBI (1♀; MNCR-A). **Guanacaste.** Macizo Miravalles, Cabro Muco Station (10°43.1'N, 84° 51.3'W; 1100 m), 15 Mar–2 Abr 2003, J. Azoifeifa. (1♀; MNCR-A).

##### Type locality.

Costa Rica. San José. Zurquí de Moravia (10°02.8'N, 84°0.6'W; 1588 m).

##### Distribution

(Fig. 14). Neotropical: Costa Rica (Cartago, Guanacaste, Puntarenas, San José).

##### Etymology.

The species epithet, *brunnea*, is of Latin derivation and means brown, referring to the dark brown color of this species, especially its head.

##### Remarks.

This species is very similar and superficially appear to be closely related to *Lamproclasiopa ecuadoriensis*, as evidenced by the generally dark brown body color and yellowish parafacies of both species. The thoracic pleural area of *Lamproclasiopa brunnea*, however, is more sparsely microtomentose. Moreover, the frontal microtomentum of *Lamproclasiopa brunnea* covers only the fronto-orbital plates and the ocellar triangle, which easily distinguishes this species from *Lamproclasiopa ecuadoriensis*.

#### Lamproclasiopa hendeli

Taxon classificationAnimaliaDipteraEphydridae

(Wirth)

Figs 15–17 , 36

Discocerina (Lamproclasiopa) facialis Hendel 1933: 79.
Discocerina (Lamproclasiopa) hendeli Wirth 1968: 7 [replacement name for Discocerina facialisHendel 1933, not Williston 1896]. Mathis and Zatwarnicki 1995: 168 [world catalog].
Lamproclasiopa hendeli . Zatwarnicki and Mathis 2001: 39 [generic combination].

##### Diagnosis.

**Figures 15–17.**
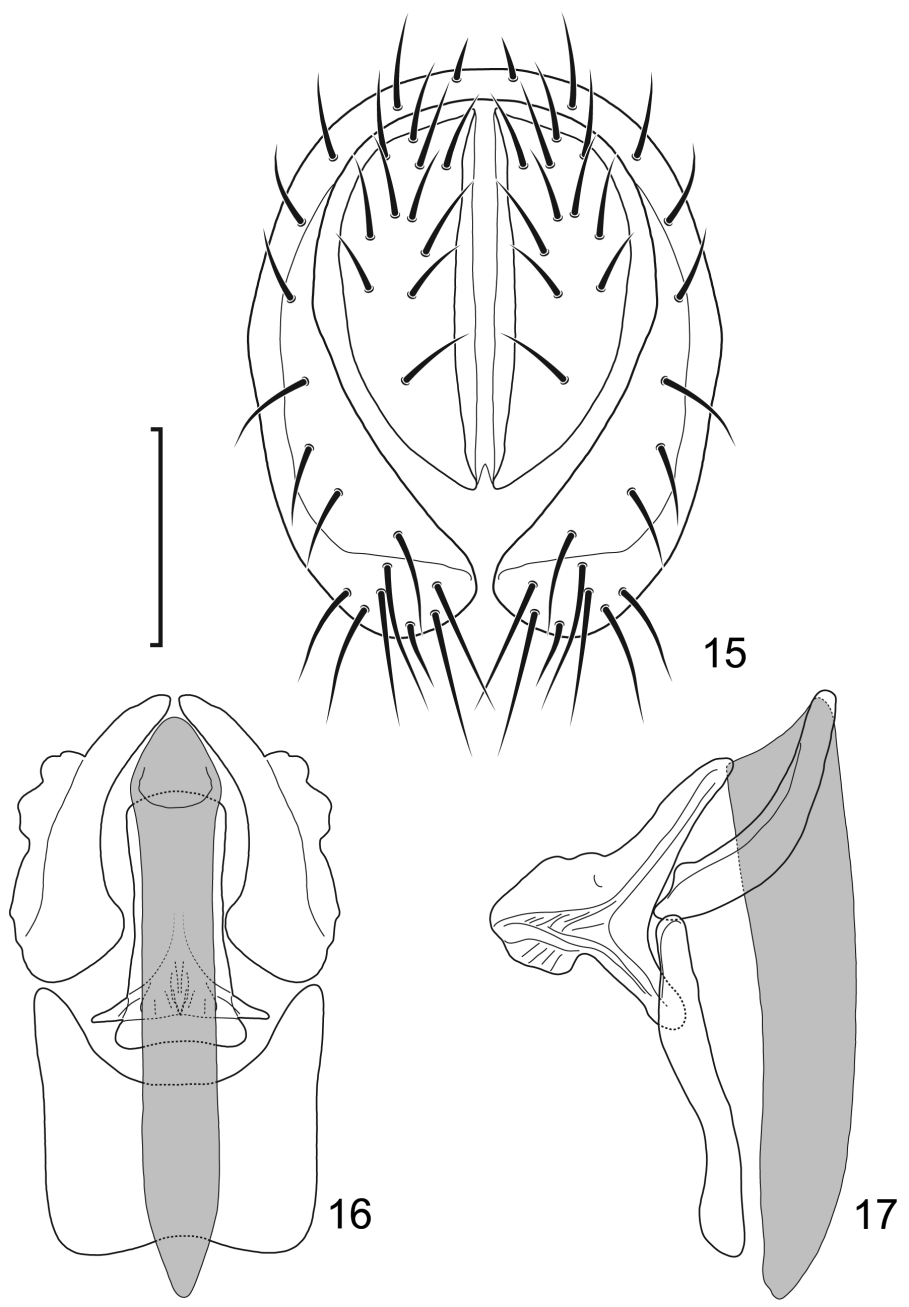
*Lamproclasiopa hendeli* (Wirth) (redraw from Zatwarnicki and Mathis 2001) **15** epandrium and cerci, posterior view **16** internal structures of male terminalia (aedeagus [shaded], phallapodeme, gonite, hypandrium), ventral view **17** same, lateral view. Scale bar = 0.1 mm.

This species is distinguished from congeners by the following combination of characters: Moderately small shore flies, body length 2.50 mm. *Head*: Frons broader than long, 1.5 times as wide as an eye; glossy black with anterior margin reddish yellow, between ocelli and fronto-orbits somewhat dull by very fine reddish brown microtomentose. Pedicel black, slightly whitish dusted dorsally; basal flagellomere red yellow, darkened along outer margin; arista with 5 long rays dorsally. Face protruded in lateral view; dorsal half of face with distinct antennal grooves, these separated by a vertical ridge, just ventrad of ventral margin of antennal grooves a transverse ridge. Ventral portion of face flat, receded towards oral margin; face silvery white, microtomentose with 2 vertical stripes toward middle. Gena moderately high, ¼ height of eye; silvery white at edge of eye; with 1 strong seta. *Thorax*: Shiny black, smooth; central portion and scutellar disc covered with very short and fine reddish brown microtomentum that reduces the shine; within this microtomentum on mesonotum, shiny black dots at bases of short setae. No prescutellar pair of setae or supra-alar seta. Legs shiny black; tarsi reddish yellow. Wing grayish hyaline, with yellow veins; costal section II about 1.5 times as long as costal section I; veins R_4+5_ and M_1_ parallel, last section of M_1_ twice as long as penultimate section. Halters with black stem and light yellow knob. Calypteres with dark brown margins and hairy. *Abdomen*: Concolorous with mesonotum, with dense, short, black setulae; tergites 3–5 almost equally long. Male terminalia (Figs 15–17): Epandrium in posterior view (Fig. 15) roundly U-shaped, except for ventral gap, oval, only slightly narrower dorsally and ventrally, widest at midheight, dorsal arch relatively narrow, each lateral arm widest ventrally, ventral margin evenly rounded, lacking medial or ventral extensions, ventral portion bearing numerous, loosely clustered, long setulae; cercus hemispherical, tapered ventrally to pointed apex, more setulose dorsally, medial margin straight; gonite in lateral view rod-like, shallowly curved, banana-like, very slightly wider toward hypandrium than toward aedeagal base, in ventral view shallowly curved, distinctly expanded on portion toward hypandrium with extension toward aedeagal base tapered to a narrow apex, lateral margin with a wide, short irregularly shaped keel; aedeagus in lateral view (Fig. 17) very elongate, narrowly triangular, almost parallel sided, tapered evenly to moderately narrow, rounded apex, in ventral view as an elongate, very narrow, parallel-sided, rod-like structure, basal end shallowly and bluntly rounded, apical 1/5 tapered toward narrow point; phallapodeme in lateral view (Fig. 17) more or less irregularly triangular, with moderately long, narrow extensions toward aedeagal base and hypandrium, keel distinct, relatively narrow, irregular, somewhat pointed apically; hypandrium in lateral view (Fig. 17) generally narrow, rod-like, very shallowly sinuous, in ventral view almost rectangular, wider than long, anterior margin shallowly emarginate, posterior margin more deeply emarginate, moderately deeply and broadly U-shaped, depth of emargination about half length of anterior portion.

##### Type material.

The holotype male of Discocerina (Lamproclasiopa) facialis Hendel was published as “1 ♂, Cuesta von Cillutiucara, Bolivien, 3200 m (Fassl) .” The holotype male is housed in the NMW.

##### Type locality.

Bolivia. La Paz: Sillutincara (= Cilluntincara) (16°17'S, 67°54'W; 3200 m).

##### Distribution

(Fig. 36). Neotropical: Bolivia (La Paz).

##### Remarks.

Although similar and apparently closely related to *Lamproclasiopa argentipicta*, this species is distinguished from this congener by the shiny black frons and blackish brown coxae.

Cresson (1946: 148) suggested that this species could be *Discocerina nitida* Cresson. We confirm that this is an included species in *Lamproclasiopa*, and further, that it is not related to *Discocerina nitida*. The illustrations of this species in Zatwarnicki and Mathis (2001) are of the holotype and are further evidence that it is a congener within *Lamproclasiopa*.

Our description of external features was extracted and interpreted from M. Kotrba’s English translation of Hendel’s original description, as we did not have access to the holotype. Although we have repeatedly inquired about and asked to examine the holotype male of this species, our requests were ignored. Fortunately, however, we have the illustrations of structures of the male terminalia that Zatwarnicki (Zatwarnicki and Mathis 2001) produced of the holotype male, and these are the basis for much of our diagnosis of this species.

### The *triangularis* group (*Lamproclasiopa triangularis*)

**Diagnosis.** Body generally subshiny to shiny black. *Head*: Frons mostly brownish black to black; frons sparsely microtomentose; ventral half of face with a microtomentose triangle, sometimes dorsal angle of triangle extended dorsally to level of base of antennae, base of triangle sometimes partially bare, otherwise face largely bare, black except for yellow to yellowish orange lateral margins adjacent to parafacial and antennal grooves; gena relatively short (gena-to-eye ratio 0.06–0.10); genal/postgenal margin rounded. *Thorax*: Presutural supra-alar seta lacking or indistinguishable from surrounding setae; katepisternum and anepisternum thinly microtomentose, generally appearing dull, not shiny. Wing hyaline, lacking any pattern or markings; vein R_2+3_ curved gently apically, not angulate subapically nor bearing a subapical stump vein. Forefemur with posteroventral setae slender, not stout and peg-like; tarsi yellowish. *Abdomen*: Male terminalia: Cerci slightly wider dorsally than ventrally; aedeagus in lateral view robust, almost rectangular, only slightly tapered on apical half.

**Remarks.** This species group currently includes only *Lamproclasiopa triangularis*, which exhibits unique character states in the triangular microtomentose area on the face and the robust, rectangular aedeagus in lateral view.

#### Lamproclasiopa triangularis sp. n.

Taxon classificationAnimaliaDipteraEphydridae

http://www.zoobank.org/1A4E189A-B737-4B14-8591-12D1102AE698

**Figures 18–19.**
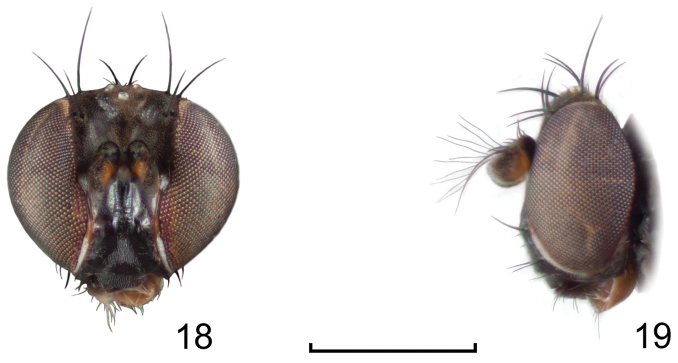
*Lamproclasiopa triangularis* sp. n., male paratype (Peru. Madre de Dios: Manu) **18** head, anterior view **19** same, lateral view. Scale bar = 0.5 mm.

**Figures 20–23.**
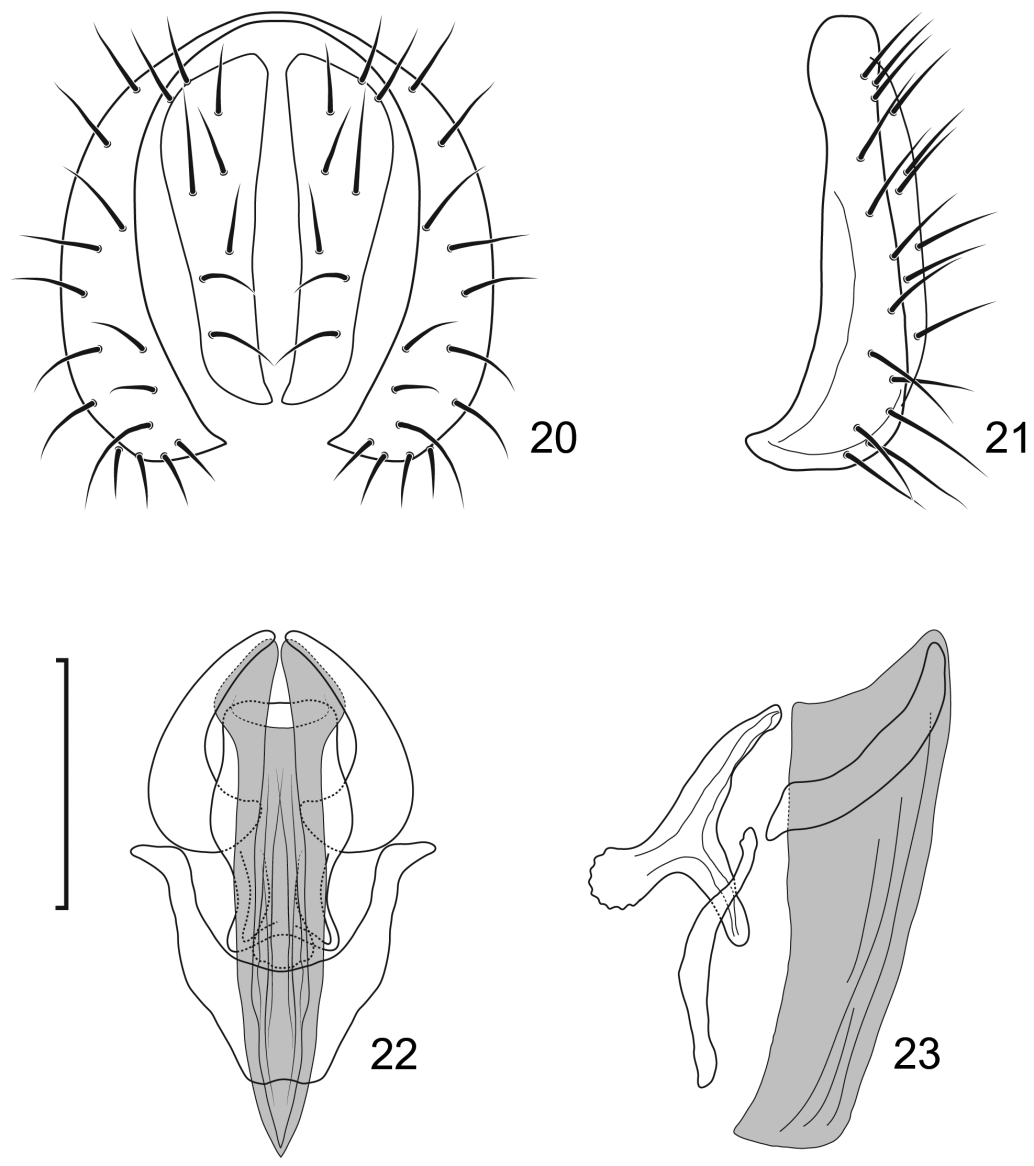
*Lamproclasiopa triangularis* sp. n., male paratype (Peru. Madre de Dios: Manu) **20** epandrium and cerci, posterior view **21** same, lateral view **22** internal structures of male terminalia (aedeagus [shaded], phallapodeme, gonite, hypandrium), ventral view **23** same, lateral view. Scale bar = 0.1 mm.

Figs 18–19 , 20–23 , 81

##### Diagnosis.

This species is distinguished from other congeners by the following combination of characters: Small to moderately small shore-fly species, body length 1.65–2.10 mm; generally black, subshiny to shiny. *Head*: Frons generally mostly brownish black to black, moderately microtomentose, subshiny; mesofrons more microtomentose, tan to brown; some specimens with 2 gray spots along ventral margin just dorsad of antennal bases. Antenna mostly black, especially scape and pedicel, only basal flagellomere with ventrobasal area with some yellow to yellowish orange coloration. Ventral half of face with a microtomentose triangle (Fig. 18), sometimes dorsal angle of triangle extended dorsally to level of base of antennae, base of triangle sometimes partially bare, otherwise face largely bare, black except for yellow to yellowish orange lateral margins adjacent to parafacial and antennal grooves; bearing 2 larger facial setae, dorsal seta at about midfacial height, dorsomesoclinate; ventral seta just dorsad of epistomal margin, slightly dorsoclinate; parafacial silvery white. Gena relatively short, gena-to-eye ratio 0.06–0.10. *Thorax*: Mesonotum uniformly whitish gray microtomentose; pleural area very sparsely microtomentose, mostly dark brown, partially subshiny; presutural supra-alar seta lacking or indistinguishable from surrounding setae. Wing hyaline, lacking any pattern or markings; costal vein ratio 0.74–0.79; M vein ratio 0.55–0.59. Legs, except tarsi, black; forefemur with posteroventral setae slender, not stout and peg-like; tarsi yellow; apical tarsomere slightly darker than other tarsomeres. *Abdomen*: Generally black, subshiny to mostly shiny, dorsum of tergites very sparsely and finely microtomentose, faintly whitish gray; sternite 3 of male rectangular, parallel sided, length twice width; sternite 4 of male rectangular, length almost twice width; sternite 5 of male a single, deeply U-shaped plate, length about twice width, opening of U posterior. Male terminalia (Figs 20–23): Epandrium in posterior view (Fig. 20) almost as wide as high, as an inverted U, dorsal arch very thin, verticolateral arms gradually becoming wider, width wider than width of cercus, in lateral view (Fig. 21) narrow, elongate, overall as a robust, irregular tear drop with an anteroventral, short, shallowly pointed projection; cercus in posterior view (Fig. 20) bar-like, elongate, narrow, with dorsal half wider than ventral half, slightly tapered from dorsum to ventral margin, not fused with ventral margin of cercal cavity, in lateral view (Fig. 21) elongate, dorsal half slightly wider than ventral half; gonite in ventral view (Fig. 22) as an inverted, robust comma, in lateral view (Fig. 23) bar-like, shallowly arched; aedeagus in lateral view (Fig. 23) robust, narrowly and irregularly rectangular, widest basally, thereafter slightly tapered to truncate apex, in ventral view (Fig. 22) elongate, narrow, narrowly ovate, acutely pointed apically; phallapodeme in lateral view (Fig. 23) as a deeply dissected triangle, extended keel rounded apically, each extended arm narrow, in ventral view (Fig. 22) narrow spindle shaped with a medial bulge, basal and apical widths subequal; hypandrium in ventral view (Fig. 22) as a robust V-shaped structure, vertex especially robust, in lateral view (Fig. 23) narrow, elongate, shallowly arched.

##### Type material.

The holotype male of *Lamproclasiopa triangularis* is labeled “PERU. Madre de Dios: Manu, Rio Manu, 250 m[,] Pakitza,12°7'S, 70°58'W [11°56.6'S, 71°16.9'W], 9–23 Sep 1988[,] Amnon Freidberg/USNM ENT 00118309 [plastic bar code label]/HOLOTYPE ♂ *Lamproclasiopa triangularis* Costa, Mathis & Marinoni, USNM [red].” The holotype is double mounted (minuten pin in a block of plastic), is in excellent condition, and is deposited in the USNM. Forty three paratypes (21♂, 22♀; DZUP, INPA, USNM) bear the same label data as the holotype but with W. N. Mathis as the collector.

##### Type locality.

Peru. Madre de Dios: Río Manu, Pakitza (11°56.6'S, 71°16.9'W; 250 m).

##### Other specimens examined.

BRAZIL. **Amazonas**: Manaus, Universidade Federal do Amazonas (03°05.9'S, 59°58.2'W; 50 m), 7 May 2010, D. and W. N. Mathis (3♂, 6♀; INPA, USNM). **Paraná**: Antonina, Reserva Natural Rio Cachoeira (25°19'S, 48°41,6'W), 8 Feb 2010, D. Negoseki (1♂; DZUP). **São Paulo**: Ubatuba, Cachoeira da Lage (23°17.6'S, 44°52.1'W; 100 m), 30 Mar 2010, D. and W. N. Mathis (1♂, 3♀; DZUP, USNM).

ECUADOR. **Orellana**: Rio Tiputini (0°38.2'S, 76°8.9'W), 12–26 Aug 1999, W. N. Mathis, A. Batista, M. Kotrba (7♂, 2♀; USNM).

GUYANA. Kaieteur Falls (5°10.7'N, 59°29.2'W; 570 m), 7 Apr 1994, W. N. Mathis (1♂, 2♀; USNM). Kanuku Mountains, Kumu River and Falls (3°15.9'N, 59°43.5'W), 28–30 Apr 1995, W. N. Mathis (1♂; USNM). Kanuku Mountains, Moco Moco River (3°18.2'N, 59°38.9'W), 29 Apr 1995, W. N. Mathis (2♂, 1♀; USNM). Lethem (25 km SE; 3°18.2'N, 59°38.9'W), 4–5 Apr 1994, W. N. Mathis (2♂; USNM).

PERU. **Madre de Dios**: Río Manu, Pakitza (11°56.6'S, 71°16.9'W; 250 m), 9–23 Sep 1988, A. Freidberg, W. N. Mathis (21♂, 23♀; USNM).

##### Distribution

(Fig. 81). Neotropical: Brazil (Amazonas, Paraná, São Paulo), Ecuador (Orellana), Guyana, Peru (Madre de Dios).

##### Etymology.

The species epithet, *triangularis*, is of Latin derivation, meaning triangular, and refers to the small triangular microtomentose area on the face of this species.

##### Remarks.

This species is distinguished from congeners by the triangle-shaped facial spot that is sparsely microtomentose. The triangle is situated medially on the ventral half of the face and is sometimes slightly elongated. Also distinguishing this species are the robust (thick) aedeagus in lateral view with its truncate apex and the narrow and elongated keel of the phallapodeme.

### The *polita* group (*Lamproclasiopa auritunica*, *Lamproclasiopa lapaz*, *Lamproclasiopa polita*)

**Diagnosis.** Body generally shiny black. *Head*: Gena high to very high, gena-to-eye ratio 0.30–0.64; merger of posterior genal margin and lateral postgenal margins forming a sharply angulate, joint margin (convergently similar to *Athyroglossa*). *Thorax*: Presutural supra-alar seta well developed; katepisternum and anepisternum mostly to entirely bare, shiny black. Wing generally hyaline to faintly infumate; vein R_2+3_ curved gently apically, not angulate subapically nor bearing a subapical stump vein. Forefemur with 4–5 stout, peg-like setae on apical third along posteroventral margin. *Abdomen*: Male terminalia: Keel of phallapodeme short and sometimes difficult to discern.

**Remarks.** This species group appears to be monophyletic based on the following two synapomorphies: (1) gena high to very high, gena-to-eye ratio 0.30–0.64; (2) merger of posterior genal margin and lateral postgenal margins forming a sharply angulate, joint margin (convergently similar to *Athyroglossa*).

Both of the new species included in the species group, *Lamproclasiopa auritunica* and *Lamproclasiopa lapaz*, are very similar to each other, and together, form a separate lineage that is distinguished by synapomorphies, such as the broad, truncate dorsal, epandrial margin in posterior view, the anterior extension of the epandrium, and the division of the aedeagus into a basiphallus and distiphallus. Certainly these characters are unique within *Lamproclasiopa*, and are the basis for the monophyly of these two species as a separate and distinct lineage.

#### Lamproclasiopa auritunica sp. n.

Taxon classificationAnimaliaDipteraEphydridae

http://www.zoobank.org/1854E006-D51C-4FA1-B50D-DA92F39754E7

Figs 24–26 , 27–30 , 36

##### Diagnosis.

**Figures 24–26.**
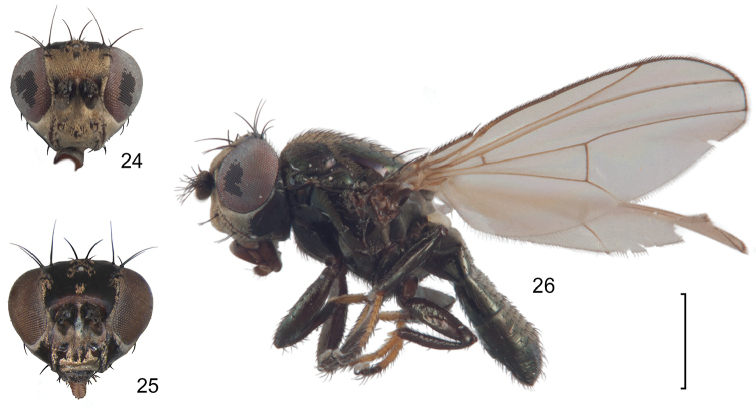
*Lamproclasiopa auritunica* sp. n. (Bolivia. Oruro: Paznã) **24** male paratype head, anterior view **25** female paratype head, anterior view **26** male paratype habitus, lateral view. Scale bar = 0.5 mm.

**Figures 27–30.**
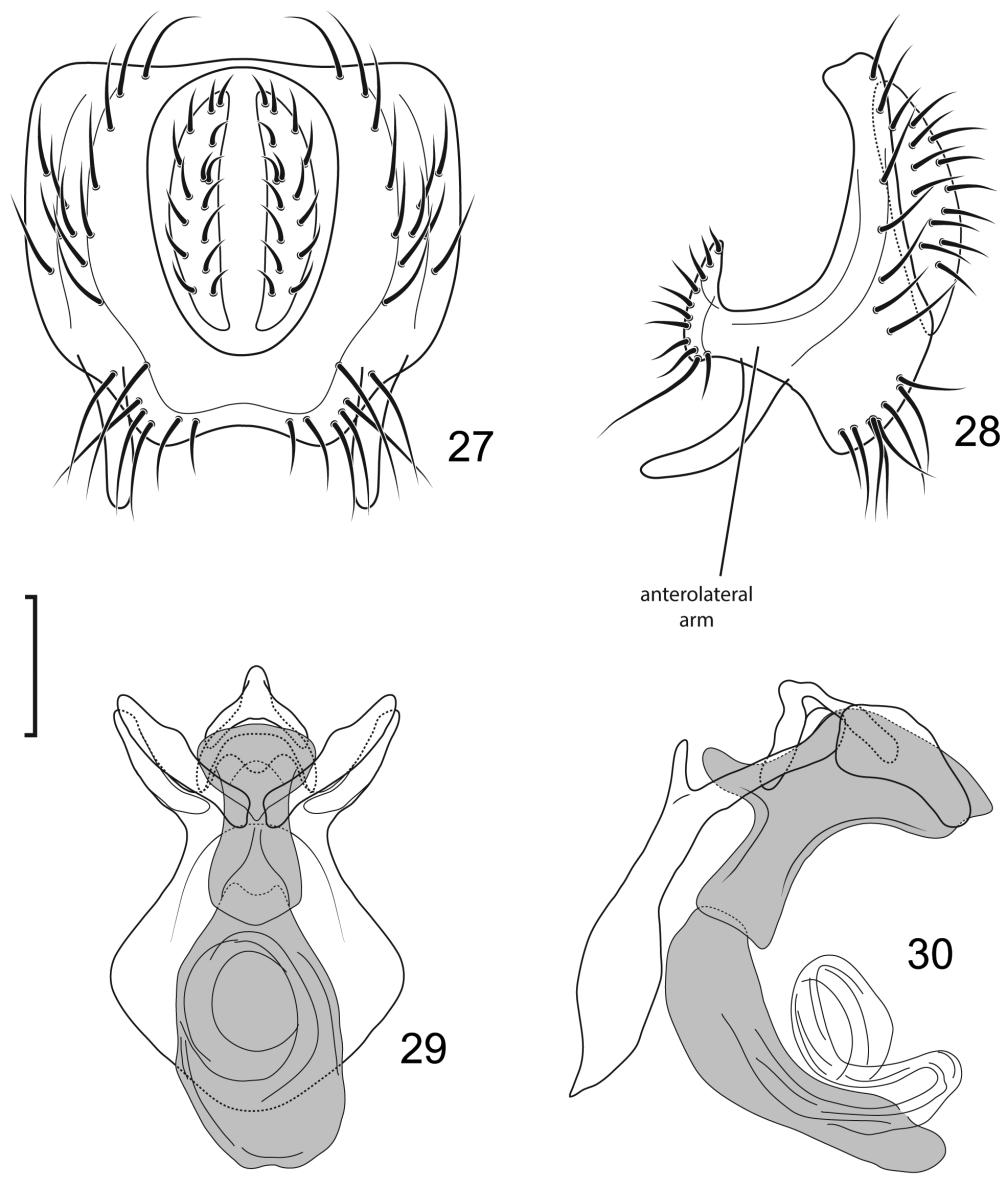
*Lamproclasiopa auritunica* sp. n. (Bolivia. Oruro: Paznã) **27** epandrium and cerci, posterior view **28** same, lateral view **29** internal structures of male terminalia (aedeagus [shaded], phallapodeme, gonite, hypandrium), ventral view **30** same, lateral view. Scale bar = 0.1 mm.

This species is distinguished from congeners by the following combination of characters: Moderately small shore flies, body length 2.30–2.80 mm; generally a shiny black species. *Head*: Frontal microtomentum sexually dimorphic; male with dense and extensive microtomentum over slightly more than anterior half of frons, also within ocellar triangle (Fig. 24), female with microtomentum only around bases of fronto-orbital setae and ocellar setae, thereafter as a thin stripe within ocellar triangle extended posteromedially, convergent within ocellar triangle, and a small medial spot just before anterior margin (Fig. 25). Antenna black, with dense microtomentum especially evident on basal flagellomere laterally; arista bearing 3–4 dorsal rays (usually 4). Face with moderately deep antennal grooves on dorsal half, shallowly angulate in lateral view, vortex of angle at midheight near dorsal facial seta, ventral half of face receded, facial microtomentum in both sexes generally dense, golden brown dorsally, becoming more silvery ventrally, female with some bare areas, especially at base of facial setae and adjacent to parafacial; parafacial and anterior half of gena densely microtomentose in male, in female with thin area microtomentose at anterior and ventral margins of eye, otherwise bare, shiny; gena very high, gena-to-eye ratio 0.42–0.64; posterior margin of gena at merger with lateral margin of postgenal sharply angulate. *Thorax*: Mesonotum shiny black, pattern of microtomentum evident as a broad band, much denser anteriorly, becoming sparse posteriorly, microtomentum extended onto scutellar disc; lateral to microtomentose band mostly bare, shiny except for microtomentose anterior surface of postpronotum and ventral margin of notopleuron; presutural supra-alar seta well developed; pleural region generally bare, shiny black. Wing hyaline to faintly infumate, faintly tannish, lacking any pattern or markings. Costal vein ratio 0.50–0.58; M vein ratio 0.59–0.78. Coxae black, shiny; forecoxa with vertical microstriae; femora and tibiae black; forefemur with 4–5 stout, peg-like setae on apical third along posteroventral margin; basal 2–3 tarsomeres yellow, apical 2–3 brownish black to dark brown. Halter with base black, knob whitish yellow. *Abdomen*: Generally shiny black; male tergite 5 truncate apically. Male Terminalia (Figs 27–30): Epandrium in posterior view (Fig. 27) irregularly hexagonal with dorsal 2/3 quadrate, as wide as high, corners rounded, ventral third with lateral margin slanted medially ventrally and ventral margin shallowly concave, dorsal portion thinly developed, lateral portions wide, each subequal to width of cercal cavity, setulae more or less evenly distributed laterally, thereafter with a gap, then clumped ventrolaterally, in lateral view (Fig. 28) more or less and irregularly L-shaped, thin dorsally, with an obtusely angulate ventral portion and a moderately narrow anterior extension with a flared, somewhat truncate anterior margin; cerci in posterior view (Fig. 27) elongate, moderately thin, generally shallowly arched, lunate, ventral and dorsal apices tapered, in lateral view elongate, narrow, elliptical; aedeagus in lateral view (Fig. 30) as 2 structures, basiphallus L-shaped with a digitiform process from one arm, distiphallus shallowly arched, wider basally, with ribbon-like extension, in ventral view (Fig. 29) with basiphallus spindle-like, elongate, distiphallus rectangularly ovate; phallapodeme in lateral view (Fig. 30) L-shaped, each arm narrow and of equal length, in ventral view Y-shaped with base shorter than either arm; gonite in lateral view irregularly pear-like, in ventral view (Fig. 29) rod-like; hypandrium in lateral view (Fig. 30) thin, elongate, width irregular and with a short, thin process near middle, in ventral view (Fig. 29) robust, with anterior 2/3 diamond-shaped, posterior third widely and shallowly U-shaped.

##### Type material.

The holotype male of *Lamproclasiopa auritunica* is labeled “**BOLIVIA. Oruro**: Paznã (S. of the town; 18°36.2'S, 66°54.7'W, 3750 m), 22 Mar 2001[,] Wayne N. Mathis/USNM ENT 00119995 [plastic bar code label]/HOLOTYPE ♂ *Lamproclasiopa auritunica* Costa, Mathis & Marinoni, USNM [red].” The holotype is double mounted (minuten pin in a plastic block) and is in very good condition, and is deposited in USNM. Three paratypes (1♂, 2♀; USNM) bear the following label data: Bolivia. Oruro: Challapata (45 km S; 19°12.9'S, 66°47.7'W, 3690 m), 22 Mar 2001, A. Freidberg, W. N. Mathis (1♂, 2♀; USNM). Bolivia. La. Paz: Tiahuanaco Ruins (16°33.7'S, 68°40.7'W; 3870m), 28 Mar 2001, W. N. Mathis (1♀;USNM); Patacayama (7 km NE; 17°9.5'S, 67°56.7'W; 3800m), 21 Mar 2001, W. N. Mathis (1♀; USNM).

##### Type locality.

Bolívia. Oruro: Paznã (S. of the town; 18°36.2'S, 66°54.7'W, 3750 m).

##### Distribution

(Fig. 36). *Neotropical*: Bolivia (La Paz, Oruro).

##### Etymology.

The species epithet, *auritunica*, is of Latin derivation, meaning coat of gold, and refers to the golden microtomentum that covers much of the head of this species.

##### Remarks.

This species is very similar and closely related to *Lamproclasiopa lapaz* and to a lesser degree *Lamproclasiopa polita* but is distinguished from these two species as follows: Female frons mostly bare, shiny black, lacking a broad, transverse stripe as in *Lamproclasiopa lapaz*; male mesonotum with a broad longitudinal band over entire length, although it is weaker posteriorly, not on anterior third only. Structures of the male terminalia are also diagnostic.

#### Lamproclasiopa lapaz sp. n.

Taxon classificationAnimaliaDipteraEphydridae

http://www.zoobank.org/6F5AC7A7-4A10-4FC3-BC73-7C97C2C5621F

Figs 31–35 , 36

##### Diagnosis.

**Figures 31–35.**
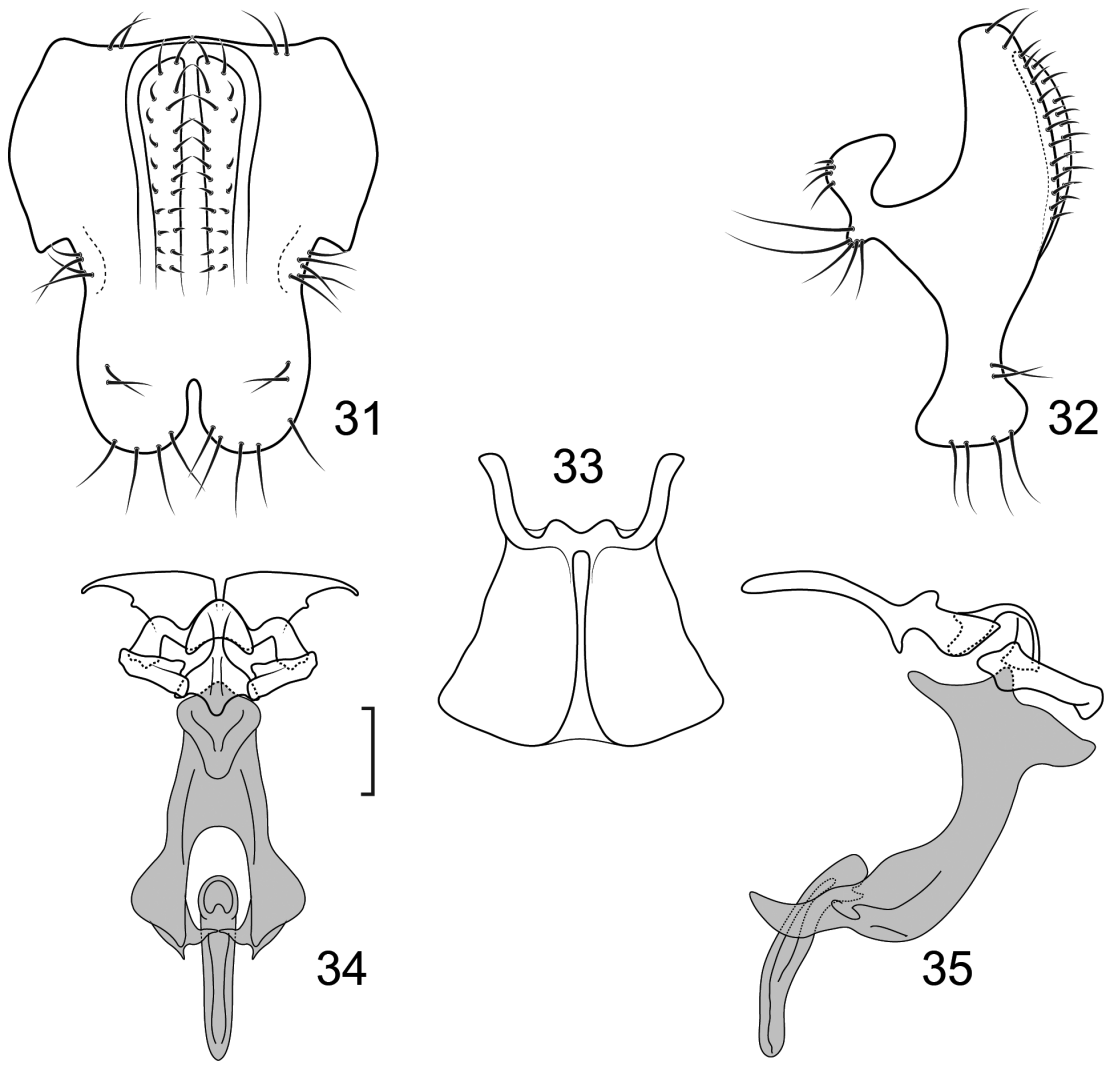
*Lamproclasiopa lapaz* sp. n. (Bolivia. La Paz: La Paz) **31** epandrium and cerci, posterior view **32** same, lateral view **33** hypandrium **34** internal structures of male terminalia (aedeagus [shaded], phallapodeme, gonite, hypandrium), ventral view **35** same, lateral view. Scale bar = 0.1 mm.

This species is distinguished from congeners by the following combination of characters: Moderately small shore flies, body length 2.40–2.97 mm; generally a shiny black species. *Head*: Frontal and facial microtomentum sexually dimorphic; male with dense and extensive microtomentum on the frons, also within ocellar triangle, anterior laterals of frons bare, shiny black, Female frons with broad, transverse stripe of microtomentum on the center; male mesonotum with microtomentum on anterior third. Antenna black, with dense microtomentum especially evident on basal flagellomere laterally; arista bearing 3–4 dorsal rays (usually 4). Face with moderately deep antennal grooves on dorsal half, shallowly angulate in lateral view, vortex of angle at midheight near dorsal facial seta, ventral half of face receded, male facial microtomentum generally dense, golden brown dorsally, becoming more silvery ventrally, female face most bare, with silvery microtometum at the ventral portion of face and at base of facial setae and adjacent to parafacial; parafacial and anterior half of gena densely microtomentose in male, in female with thin area microtomentose at anterior and ventral margins of eye, otherwise bare, shiny; gena very high, gena-to-eye ratio 0.42–0.54; posterior margin of gena at merger with lateral margin of postgenal sharply angulate. *Thorax*: Mesonotum shiny black, male mesonotum with microtomentum on anterior third, with a thin lateral extension at level of suture and extended along posterior margin of notopleuron; lateral to microtomentose band mostly bare, shiny except for microtomentose anterior surface of postpronotum and ventral margin of notopleuron; presutural supra-alar seta well developed; pleural region generally bare, shiny black. Wing hyaline to faintly infumate, faintly tannish, lacking any pattern or markings. Costal vein ratio 0.40-0.51; M vein ratio 0.69–0.83. Coxae black, shiny; forecoxa with some lateral areas microtomentose but lacking vertical microstriae; femora and tibiae black; forefemur with 4–5 stout, peg-like setae on apical third along posteroventral margin; basal 2–3 tarsomeres yellow, apical 2–3 brownish black to dark brown. Halter with base black, knob whitish yellow. *Abdomen*: Generally shiny black; male tergite 5 truncate apically. Male Terminalia (Figs 31–35): Epandrium in posterior view (Fig. 31) with dorsal half transversely rectangular, lateral margin shallowly convex, dorsal margin broadly truncate, very thin above cercal cavity, ventral half thinner than dorsal half, demarcation sharply angulate, thereafter ventral extensions almost parallel sided, ventral margin broadly bilobed with moderately deep, thin, incision, setulae clumped, at ventral margin, at beginning of ventral half and at 2 sites along dorsal margin, in lateral view (Fig. 32) with posterior portion linear, thinnest dorsally and subventrally, thereafter ventrally enlarged, clavate, with well-developed anterior, hook-like extension, hook angulate rather than rounded, bearing setulae at vortices of angles; cerci in posterior view (Fig. 32) narrow, elongate, rod-like, slightly wider dorsally, apparently fused ventrally with ventral margin of cercal cavity, in lateral view (Fig. 33) narrowly lunate; aedeagus in lateral view (Fig. 35) as 2 structures of differing lengths, elongate basiphallus curved, irregularly tapered, base of basiphallus T-shaped, bar formed by pointed, lateral projections, apex of basiphallus acutely pointed and more curved, distiphallus less than half length of basiphallus, shallowly curved, otherwise rod-like, in ventral view (Fig. 34) with basiphallus as a thick, inverted Y, incised gap narrowly and deeply U-shaped with a heart-shaped extension at base, distiphallus with base within apical gap of basiphallus, narrow, straight, rod-like; phallapodeme in lateral view (Fig. 35) C-shaped, each arm expanded apically, in ventral view as 2 stacked, moderately broad, short arrowheads; gonite in lateral view irregularly clavate, narrow, elongate, straight, rod-like, in ventral view (Fig. 34) shorter than gonite in lateral view, rod-like; hypandrium in lateral view (Fig. 35) thin, elongate, irregularly clavate basally with midlength, short projections, in ventral view (Fig. 34) as 2 irregular, almost parallel, rectangular sclerites, wider anteriorly than posteriorly, with a W-shaped base with narrow arms extended posteriorly and slightly laterally, and 2 short medial bumps along base.

##### Type material.

The holotype male of *Lamproclasiopa lapaz* is labeled “**BOLIVIA. La Paz**: La Paz (6 km NE; 16°25.7'S, 68°04.3'W; 4130m), 19 Mar 2001[,] Wayne N. Mathis/USNM ENT 00119994 [plastic bar code label]/HOLOTYPE ♂ *Lamproclasiopa lapaz* Costa, Mathis & Marinoni USNM [red].” The holotype is double mounted (minuten pin in a block of plastic), is in good condition (abdomen removed, dissected, parts in an attached microvial), and is deposited in the USNM. Two female paratypes bear the following label data: Bolivia. La Paz (NE; 16°27.4'S, 68°06'W; 3940m), 19 Mar 2001, W. N. Mathis (2♀; USNM).

##### Type locality.

BOLIVIA. La Paz: La Paz (6 km NE; 16°25.7'S, 68°04.3'W; 4130m).

##### Distribution

**Figure 36.**
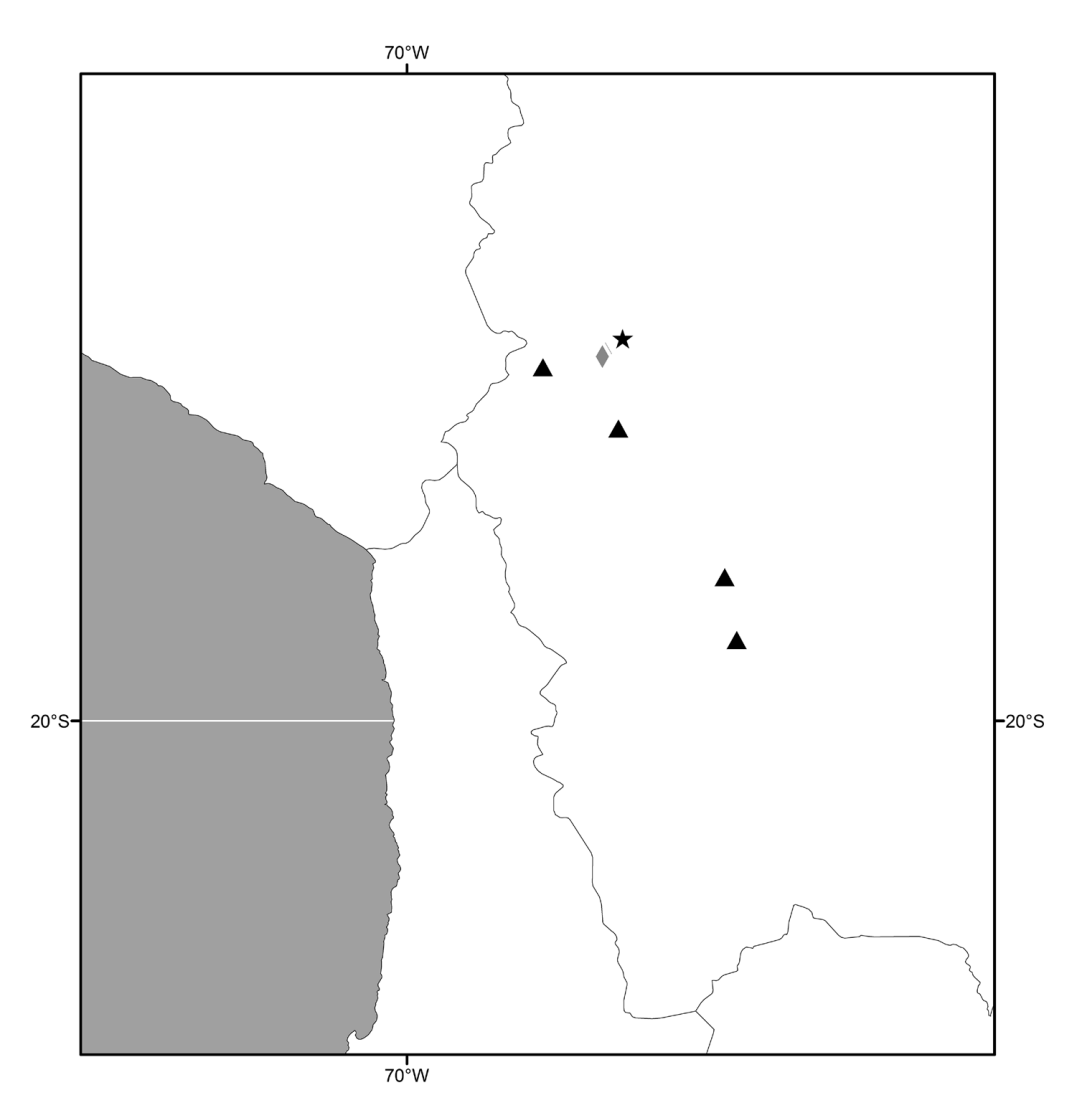
Distribution map of *Lamproclasiopa auritunica* sp. n. (▲); *Lamproclasiopa lapaz* sp. n. (♦). *Lamproclasiopa hendeli* (★).

(Fig. 36). *Neotropical*: Bolivia (La Paz).

##### Etymology.

The species epithet, *lapaz*, refers to the capital of Bolivia, La Paz, where the type series was collected. La Paz is Spanish for peace, which we embrace and recommend to all.

##### Remarks.

This species is very similar both of the other species of the *polita* group, especially *Lamproclasiopa auritunica*, but is distinguished from these two species as follows: Female frons with broad, transverse stripe on anterior half (female frons in *Lamproclasiopa auritunica* is mostly bare, shiny black); male mesonotum with microtomentum on anterior third only (male mesonotum in *Lamproclasiopa auritunica* has a broad longitudinal band over entire mesonotal length, although it is weaker posteriorly). Shapes of structures of the male terminalia are also diagnostic.

#### Lamproclasiopa polita

Taxon classificationAnimaliaDipteraEphydridae

(Edwards)

**Figures 37–38.**
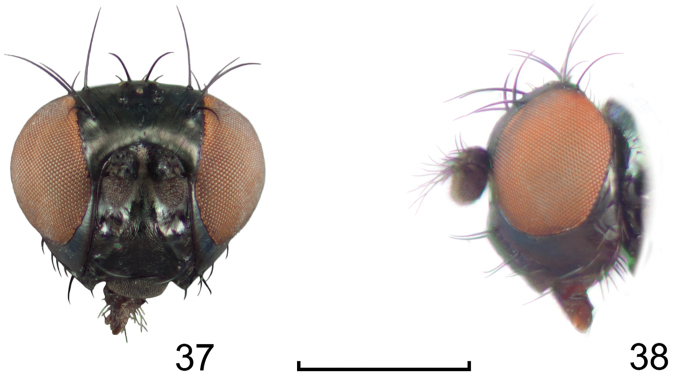
*Lamproclasiopa polita* (Edwards). (Chile. Osorno: Anticura) **37** head, anterior view **38** same, lateral view. Scale bar = 0.5 mm.

**Figures 39–42.**
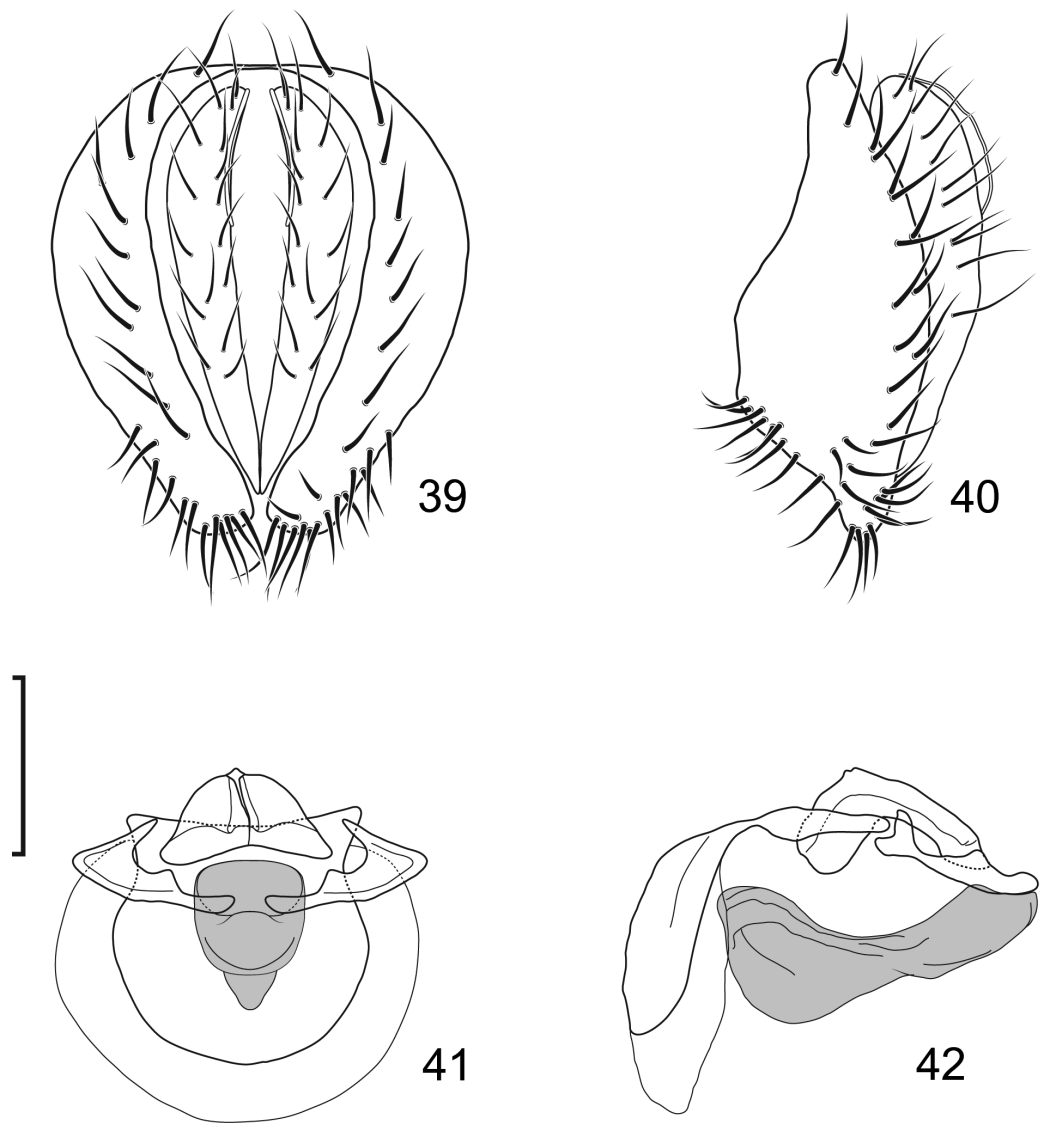
*Lamproclasiopa polita* (Edwards). (Chile. Osorno: Anticura) **39** epandrium and cerci, posterior view **40** same, lateral view **41** internal structures of male terminalia (aedeagus [shaded], phallapodeme, gonite, hypandrium), ventral view **42** same, lateral view. Scale bar = 0.1 mm.

Figs 37–38 , 39–42 , 104

Ditrichophora polita Edwards 1933: 117.
Discocerina (Basila) polita . Cresson 1946: 149 [generic combination]. Wirth 1968: 7 [Neotropical catalog]. Lizarralde de Grosso 1989: 24 [list, Argentina]. Lizarralde de Grosso et al. 2011: 13 [Argentina catalog]. Mathis and Zatwarnicki 1995: 165 [world catalog].
Lamproclasiopa polita . Zatwarnicki and Mathis 2001: 39 [generic combination].

##### Diagnosis.

This species is distinguished from other congeners by the following combination of characters: Small to moderately small shore flies, body length 1.60–2.70 mm; generally a shiny black species. *Head*: Frons shiny black. Antenna black except for basoventral yellowish orange to orange of basal flagellomere. Face black except for silvery gray, microtomentose antennal grooves, microtomentum sometimes extended ventrally onto ventral portion of face, in lateral view rounded, obtusely angulate, greatest extension at midheight. Antenna black. Gena high; gena-to-eye ratio 0.30–0.34. *Thorax*: Mesonotum and pleural areas shiny black; presutural supra-alar seta well developed. Wing hyaline, immaculate; costal vein ration 0.43–0.45; M vein ratio 0.52–0.57. Legs black, mostly shiny except for yellow basal 2 tarsomeres; forefemur with 4–5 stout, peg-like setae on apical third along posteroventral margin; *Abdomen*: Tergites shiny black, almost completely bare of microtomentum; male tergite 5 more or less triangular, posterior margin narrowly rounded. Male terminalia (Figs 39–42): Epandrium in posterior view (Fig. 39) more or less oval, flattened dorsally, narrowed ventrally, setulae more evident ventrally, in lateral view (Fig. 40) longer than wide, ventral half robust, widest just ventrad of midheight, narrowly rounded at apex; cerci in posterior view (Fig. 39) narrow, elongate, slightly curved, ventral apex narrowly pointed, in lateral view (Fig. 40) as an elongated teardrop, shallowly curved, wider dorsally, ventral portion becoming narrower ventrally; gonite in lateral view (Fig. 42) elongate, posterior margin more or less evenly developed, anterior margin with angular protuberance, ventral apex shallowly bifurcate, in ventral view (Fig. 41) robustly developed medially, apices thin, angulate laterally; aedeagus in lateral view (Fig. 42) clavate, base narrower than globular apex, rounded apically, in ventral view (Fig. 41) with base quadrate with short triangular extension; phallapodeme in lateral view (Fig. 42) robustly L-shaped, in ventral view (Fig. 41) dome-like; hypandrium in lateral view L-shaped, anterior portion longer and more robustly developed than narrow, posterior portion, in ventral view (Fig. 41), slightly more than semicircular, broadly and evenly rounded.

##### Type material.

The holotype female of *Ditrichophora polita* Edwards is labeled “Holotype/Type/Argentina: Terr. Río Negro. F.&M. Edwards. B.M. 1927–63./Lake Gutiérrez 3–14.xi.1926./*Ditrichophora polita* Edw. F. W. Edwards det. 1932/HOLOTYPE *Ditrichophora polita* Edwards det. J.E. Chainey, 1995/NHMUK010240990. The holotype is double mounted (glued to a plastic triangle), is in good condition, and is deposited in the BMNH.

##### Type locality.

Argentina. Río Negro: Lake Gutiérrez (41°11.5'S, 71°23.7'W).

##### Other specimens examined.

CHILE. **Atacama**: Huasco (28°28'S, 71°13.1'W), 21 Oct 1957, L. E. Peña (1♂, 1♀; USNM). **Cautin**: Temuco (20 km E; 38°44'S, 72°35'W), 7 Jan 1951, A. E. Michelbacher, E. S. Ross (1♀; USNM). **Coquimbo**: Incahuasi (27°02'S, 68°18'W), 30 Sep 1952, P. G. Kuschel (13♂, 7♀; USNM); Ovalle (32 km SE; 30°36'S, 71°11'W), 12 Dec 1950, A. E. Michelbacher, E. S. Ross (3♂, 2♀; USNM). **Lanquihue**: Peulla (41°28'S, 72°57.7'W) (1?; BMNH); Puerto Varas (41°18.6'S, 72°59.6'W) (1♂; BMNH). **O’Higgins**: Río Claro (5 km N Rengo; 34°24'S, 70°52'W; 300 m), 23 Jan 1978, W. N. Mathis (1♀; USNM). **Osorno**: Anticura (4 km W; 37°40'S, 72°01'W; 400 m), 3 Feb 1978, W. N. Mathis (3♂, 4♀; USNM); Anticura (1 km W; 40°39'S, 72°10'W; 430 m), 5–12 Feb 1978, W. N. Mathis (1♂; USNM); Lago Puyehue (SE shore; 40°45'S, 72°25.2'W), 6–10 Feb 1978, W. N. Mathis (4♂, 1♀; USNM); Lago Rupanco, El Encanto (40°49'S, 72°28'W), 6 Feb 1978, W. N. Mathis (1♂, 1♀; USNM); Laguna El Pato (41°10'S, 73°40'W; 1100 m), 13 Feb 1978, W. N. Mathis (2♂, 2♀; USNM); Termas de Aguas Calientes (1 km SE; 40°41'S, 72°21'W; 530 m), 7–8 Feb 1978, W. N. Mathis (2♂, 2♀; USNM). **Santiago**: El Alfalfal (33°30'S, 70°11'W; 1320 m), 22 Jan 1978, W. N. Mathis (1♂, 2♀; USNM); Quebrada de la Plata (near Maipú; 33°30'S, 70°55'W; 550 m; Malaise trap), 12 Mar 1986, M. E. Irwin (1♂; USNM). **Talca**: Río Lircay (11 km N Talca; 35°23'S, 71°39'W; 85 m), 23 Jan 1978, W. N. Mathis (2♂, 7♀; USNM).

##### Distribution

(Fig. 104). Neotropical: Argentina (Río Negro), Chile (Atacama, Cautin, Coquimbo, Lanquihue, Malleco, O’Higgins, Osorno, Santiago, Talca).

##### Remarks.

Although similar to *Lamproclasiopa auritunica* and *Lamproclasiopa lapaz* in having a high gena (gena-to-eye ratio 0.30–0.34), this species is unlike these two species by having a nearly bare and shiny male frons; a triangular-shaped male tergite five, which is narrowly rounded posteriorly; a rounded epandrium, and an aedeagus without any kind of division.

### The *ecuadoriensis* group (*Lamproclasiopa ecuadoriensis*, *Lamproclasiopa zerafael*)

**Diagnosis.** Body with extensive surfaces sparsely to densely microtomentose. *Head*: Frons and face generally unicolorous; gena relatively short (gena-to-eye ratio 0.05–0.12); genal/postgenal margin rounded. *Thorax*: Presutural supra-alar seta variable, well developed in *Lamproclasiopa ecuadoriensis*, lacking in *Lamproclasiopa zerafael*; katepisternum and anepisternum thinly microtomentose, generally appearing dull, not shiny. Wing generally hyaline to very faintly infumate (*Lamproclasiopa mancha* with crossveins r-m and dm-cu with darkened cloud); vein R_2+3_ curved gently apically, not angulate subapically nor bearing a subapical stump vein. Forefemur with 4–5 stout, peg-like setae on apical third along posteroventral margin. *Abdomen*: Male terminalia: Keel of phallapodeme short and sometimes difficult to discern.

**Remarks.** This species group, like the *hendeli* group, is mostly based on homoplasious characters, and we cannot confirm its monophyly. The two included species are similar to each other and the species group can be diagnosed. These are the bases for recognition of this species group. Structures of the male terminalia of *Lamproclasiopa zerafael* are quite different from all congeners, especially the very robust aedeagus that is slightly asymmetrical, and the very wide and dissected hypandrium in ventral view.

#### Lamproclasiopa ecuadoriensis sp. n.

Taxon classificationAnimaliaDipteraEphydridae

http://www.zoobank.org/148D944E-D393-46B5-A9F5-1E9031EA655E

**Figures 43–44.**
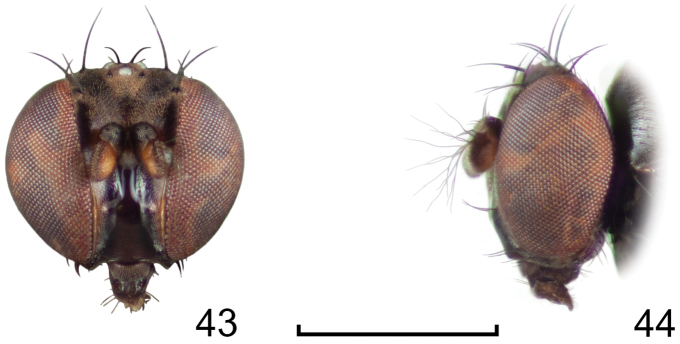
*Lamproclasiopa ecuadoriensis* sp. n. (Ecuador. Orellana: Rio Tiputini) **43** head, anterior view **44** same, lateral view. Scale bar = 0.5 mm.

**Figures 45–48.**
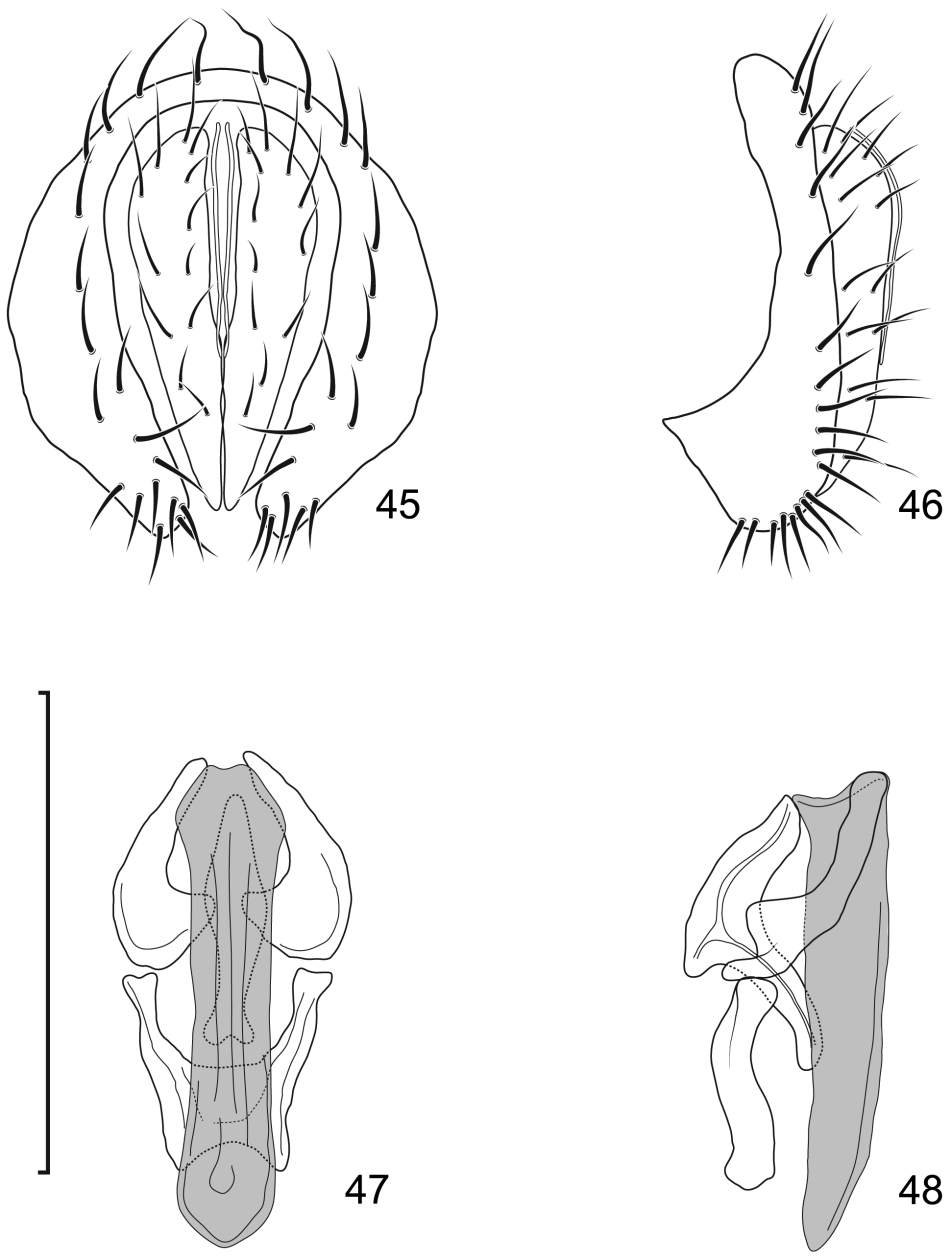
*Lamproclasiopa ecuadoriensis* sp. n. (Ecuador. Orellana: Rio Tiputini) **45** epandrium and cerci, posterior view **46** same, lateral view **47** internal structures of male terminalia (aedeagus [shaded], phallapodeme, gonite, hypandrium), ventral view **48** same, lateral view. Scale bar = 0.1 mm.

Figs 43–44 , 45–48 , 104

##### Diagnosis.

This species is distinguished from other congeners by the following combination of characters: Small shore-fly species, body length 1.55–1.80 mm; generally black, subshiny to shiny. *Head*: Frons mostly brownish black to black, sparsely brownish microtomentose, more so on anterior portion, subshiny, ocellar triangle extended to anterior margin of frons, some specimens with grayish red areas along anterior margin just dorsad of antennal bases, parafrons with narrowly oval, densely microtomentose areas at anterolateral corner. Antenna mostly black, only basal flagellomere with ventrobasal area with some yellowish to yellowish orange coloration. Face narrow, mostly shiny black, especially over greater medial portion, extreme lateral margin adjacent to parafacial yellowish, narrow whitish gray, transverse band just ventrad of antennal base and through dorsal portion of antennal grooves, ventral half of face slightly receded; bearing 2 larger facial setae, dorsal seta at about midfacial height, dorsomesoclinate; ventral seta just dorsad of epistomal margin, slightly dorsoclinate; parafacial thin, yellow dorsally, adjacent to eye, black ventrally and extended to gena. Gena short, gena-to-eye ratio 0.04–0.06. *Thorax*: Mesonotum uniformly sparsely microtomentose, brownish black to black, subshiny; presutural supra-alar seta well developed; pleural region black; dorsal 2/3 of anepisternum finely granulose, subshiny, anteroventral portion smooth, shiny. Wing hyaline, lacking any pattern or markings; costal vein ratio 0.80–0.81; M vein ratio 0.61–0.64. Legs, except tarsi, black; tarsi yellow; apical 1–2 tarsomeres darker, tan to brown; forefemur with sparse row of 4–5, stouter, spine-like setae along apical half of posteroventral surface. *Abdomen*: Generally black, subshiny to mostly shiny, dorsum of tergites very sparsely and finely microtomentose, faintly whitish gray; sternite 3 of male rectangular, parallel sided, length twice width; sternite 4 of male rectangular, length almost twice width; sternite 5 of male as 2 sternites, length nearly twice greatest width, anterior margin narrow, becoming slightly wider on anterior 1/3, thereafter tapered to a posterolateral point, lateral margin straight, medial margin angulate. Male terminalia (Figs 45–48): Epandrium in posterior view (Fig. 45) robustly oval, arched and thin dorsally, gradually becoming wider ventrally than narrowed on apical 1/3, in lateral view (Fig. 46) with dorsal 2/3 thirds narrow, strap-like, thereafter ventrally abruptly widened with anterior, pointed extension, ventral margin broadly rounded; cerci in posterior view (Fig. 45) elongate, narrowly semicircular, ventral apex more acutely pointed than more widely produced dorsal margin, in lateral view (Fig. 46) irregularly, narrowly semihemispherical, wider subdorsally than ventrally; gonite in lateral view narrowly rod-like, arched, only slightly wider toward aedeagal base than toward hypandrium, in ventral view (Fig. 47) robustly hook-like with shank of hook narrow and rounded portion very robustly developed; phallapodeme in lateral view (Fig. 48) L-shaped, arm extended to aedeagal base slightly more robust, length of both arms about equal, in ventral view (Fig. 47) as a dog bone, expanded at each apex; hypandrium in lateral view (Fig. 48) elongate, robust, sinuous, more or less parallel sided, in ventral view (Fig. 47) as a very robust H with long posterior arms, lateral margins conspicuously sinuous, anterior emargination shallowly concave, posterior emargination deep, broadly U-shaped.

##### Type material.

The holotype male of *Lamproclasiopa ecuadoriensis* is labeled “**ECUADOR.** Prt. Or[e][l]lana: RioTiputini (0°38.2'S, 76°8.9'W), 12–26 Aug 1999,W.N.Mathis, A. Baptista, M. Kotrba/USNM ENT 00118307 [plastic bar code label]/HOLOTYPE ♂ *Lamproclasiopa ecuadoriensis* Costa, Mathis & Marinoni USNM [red].” The holotype is double mounted (minuten pin in a plastic block), is in excellent condition, and is deposited in the USNM. Four paratypes (3♂, 1♀; DZUP, USNM) bear the same label data as the holotype.

##### Type locality.

Ecuador. Orellana: Río Tiputini Biodiversity Station (0°38.2'S, 76°8.9'W).

##### Distribution

(Fig. 104). *Neotropical*: Ecuador (Orellana).

##### Etymology.

The species epithet, *ecuadoriensis*, refers to the country of Ecuador, where this species was collected.

##### Remarks.

This species is similar to *Lamproclasiopa laevior* and *Lamproclasiopa polita*, although it can be distinguished from congeners by the densely microtomentose anterolateral, narrowly oval black velvet spots on the frons; the narrow, shiny black face; the comparatively elongate costal section III (section III slightly less than section II); and the shape of structures of the male terminalia (Figs 45–48).

#### Lamproclasiopa zerafael sp. n.

Taxon classificationAnimaliaDipteraEphydridae

http://www.zoobank.org/3BA512CE-7CF2-4236-868D-0833AC936736

**Figures 49–50.**
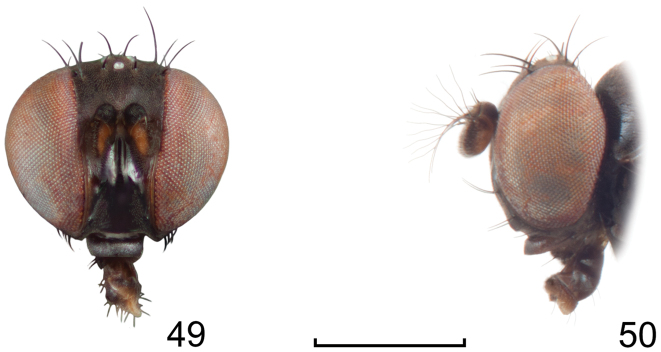
*Lamproclasiopa zerafael* sp. n. (Brazil. Amazonas: Manaus) **49** head, anterior view **50** same, lateral view. Scale bar = 0.5 mm.

**Figures 51–54.**
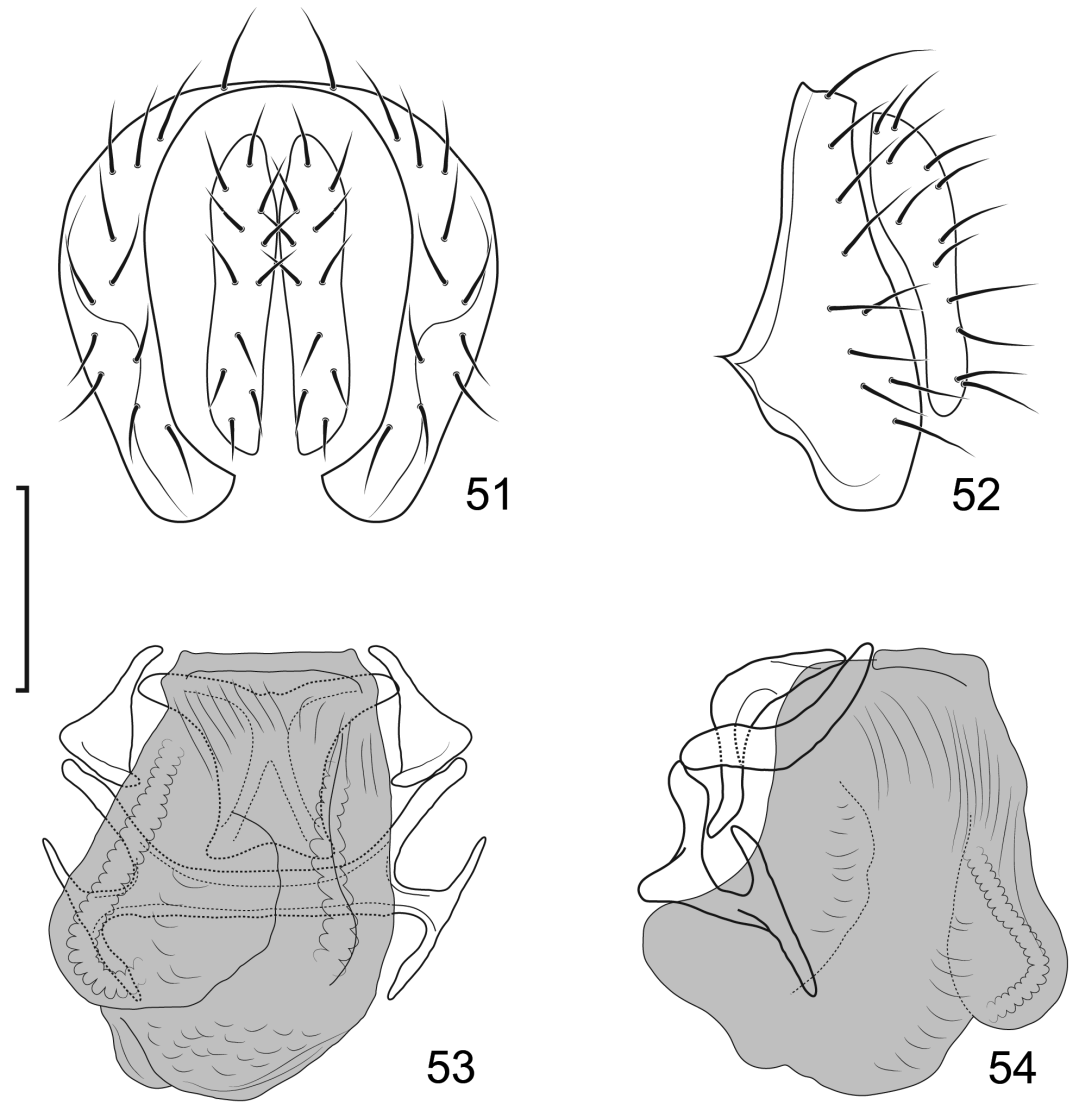
*Lamproclasiopa zerafael* sp. n. (Brazil. Amazonas: Manaus) **51** epandrium and cerci, posterior view **52** same, lateral view **53** internal structures of male terminalia (aedeagus [shaded], phallapodeme, gonite, hypandrium), ventral view **54** same, lateral view. Scale bar = 0.1 mm.

Figs 49–50 , 51–54 , 104

##### Diagnosis.

This species is distinguished from other congeners by the following combination of characters: Small to moderately small shore-fly species, body length 1.50–2.05 mm; generally black, subshiny to shiny. *Head*: Frons mostly brownish black to black, sparsely brownish microtomentose, more so on anterior portion, subshiny, some specimens with 2 gray spots along ventral margin just dorsad of antennal bases. Antenna mostly black, only basal flagellomere with ventrobasal area with some yellowish to yellowish orange coloration. Face mostly shiny black, especially medially and laterally, between with some areas sparsely microtomentose and in antennal grooves, dorsal half; antennal grooves evident , dorsad of dorsoclinate facial pair of setae; ventral half of face slightly receded; bearing 2 larger facial setae, dorsal seta at about midfacial height, dorsomesoclinate; ventral seta just dorsad of epistomal margin, slightly dorsoclinate; parafacial thin, black. Gena relatively short, gena-to-eye ratio 0.06–0.07. *Thorax*: Mesonotum uniformly sparsely microtomentose, brownish black to black; presutural supra-alar seta lacking or indistinguishable from surrounding setae; pleural region black; dorsal 2/3 of anepisternum finely granulose, subshiny, anteroventral portion smooth, shiny. Wing hyaline, lacking any pattern or markings; costal vein ratio 0.75–0.89; M vein ratio 0.58–0.61. Legs, except tarsi, black; tarsi yellow; apical 1–2 tarsomeres darker, tan to brown; forefemur with sparse row of 4–5, stouter, spine-like setae along apical half of posteroventral surface. *Abdomen*: Generally black, subshiny to mostly shiny, dorsum of tergites very sparsely and finely microtomentose, faintly whitish gray. Male terminalia (Figs 51–54): Epandrium in posterior view (Fig. 51) almost as wide as high, as an inverted U, dorsal arch very thin, vertical, lateral arms essentially parallel sided, wider than width of cercus, in lateral view (Fig. 52) widest at ventral 1/3, ventral margin step-wise rounded, overall as a robust tear drop with an anterior, short, shallowly pointed projection at widest width; cercus in posterior view (Fig. 51) bar-like, elongate, narrow, parallel sided, not fused with ventral margin of cercal cavity, in lateral view (Fig. 52) elongate, dorsal half slightly wider than ventral half; gonite in ventral view (Fig. 53) triangular, with basal angle projected into narrow process, in lateral view (Fig. 54) obtusely angulate, extension toward aedeagal base slightly thinner; aedeagus in lateral view (Fig. 54) irregular, wider apically, margin irregular, in ventral view (Fig. 53) slightly wider subapically, irregularly rounded apically; phallapodeme in lateral view (Fig. 54) angulate, L-shaped, extension toward hypandrium shallowly angulate subapically, in ventral view (Fig. 53) spindle shaped, with basal portion much wider than apical portion; hypandrium in ventral view (Fig. 53) as a very wide and short structure, anterior and posterior emarginations shallow, anterior arms with oblique crossbar, in lateral view (Fig. 54) as an irregular H, with lateral portions of H irregular.

##### Type material.

The holotype male of *Lamproclasiopa zerafael* is labeled “**BRAZIL.** Amazonas: Reserva Ducke (02°55.8'S, 59°58.5'W; 40 m), 5 May 2010, D. & W. N. Mathis/USNM ENT 00118311 [plastic bar code label]/HOLOTYPE ♂ *Lamproclasiopa zerafael* Costa, Mathis & Marinoni, INPA [red].” The holotype is double mounted (minuten pin in a plastic block), is in excellent condition, and is deposited in INPA. Fourteen paratypes (11♂, 2♀; DZUP, INPA, USNM) bear the same label data as the holotype. Other paratypes are as follows: BRAZIL. **Amazonas**: Manaus, Universidade Federal do Amazonas (03°05.9'S, 59°58.2'W; 50 m), 7 May 2010, D. and W. N. Mathis (5♂, 11♀; DZUP, INPA, USNM); Reserva Cuieiras (02°35.2'S, 60°07.2'W; 110 m), 8 May 2010, D. and W. N. Mathis (3♂; INPA, USNM).

##### Type locality.

Brazil. Amazonas: Reserva Ducke (02°55.8'S, 59°58.5'W; 40 m).

##### Distribution

(Fig. 104). *Neotropical*: Brazil (Amazonas).

##### Etymology.

The species epithet, *zerafael*, refers to José (Zé) Albertino Rafael, student of Diptera and Zoraptera (especially the Amazonian fauna) and who kindly hosted and guided us while in Manaus, Amazonas. The name is a noun in apposition.

##### Remarks.

This species is distinguished from congeners, especially *Lamproclasiopa triangularis*, by having a sparsely microtomentose body generally, a mostly shiny black face; a short gena (height about half height of basal flagellomere), a hyaline wing, a blackish yellow foretarsus. The shape of structures of the male terminalia also distinguishes this species, especially the relatively gross, thickened aedeagus that is slightly asymmetrical, the wide and thinly dissected hypandrium, and the funnel-shaped gonites in ventral view.

### The *painteri* group (*Lamproclasiopa balsamae*, *Lamproclasiopa mancha*, *Lamproclasiopa painteri*)

**Diagnosis.** Body with extensive surfaces sparsely to densely microtomentose. *Head*: Frons and face generally unicolorous; gena relatively short (gena-to-eye ratio between 0.05–0.10); genal/postgenal margin rounded. *Thorax*: Presutural supra-alar seta well developed; katepisternum and anepisternum thinly microtomentose, generally appearing dull, not shiny. Wing with numerous dark spots (*Lamproclasiopa balsamae*, *Lamproclasiopa painteri*) or with darkened clouds over crossveins r-m and especially over dm-cu (*Lamproclasiopa mancha*); vein R_2+3_ either angulate subapically and bearing a stump vein with a posteroapical orientation, a second stump vein near middle (*Lamproclasiopa balsamae*, *Lamproclasiopa painteri*) or vein R_2+3_ with apex more abruptly curved toward costa (*Lamproclasiopa mancha*). Forefemur with 4–5 stout, peg-like setae on apical third along posteroventral margin.

**Remarks.** This species group comprises species with some pattern in the wing and is thus distinctive from all others, which have mostly hyaline or very faintly infumate wings. The pattern, however, differs. For *Lamproclasiopa balsamae* and *Lamproclasiopa painteri* the pattern comprises numerous distinctive brown spots, and vein R_2+3_ is distinctly angulate subapically with the apices abruptly angled subapically toward costa. At the vertex of the abrupt, subapical angle there is also a stump vein and often another stump vein near middle of this vein. Certainly these two species form a monophyletic lineage. The inclusion of *Lamproclasiopa mancha* in this species group may be artificial, as the pattern in the wing is quite different (see species description of *Lamproclasiopa mancha*).

#### Lamproclasiopa balsamae

Taxon classificationAnimaliaDipteraEphydridae

(Cresson)

Figs 55–58 , 59

Ditrichophora balsamae Cresson 1930: 77.
Discocerina (Basila) balsamae . Cresson 1946: 149 [generic combination, review]. Wirth 1968: 7 [Neotropical catalog]. Mathis and Zatwarnicki 1995: 165 [world catalog].
Lamproclasiopa balsamae . Zatwarnicki and Mathis 2001: 39 [generic combination].

##### Diagnosis.

This species is easily distinguished from congeners by the following combination of characters: Small shore flies, body length 1.65–1.85 mm. *Head*: Frons bi- or tricolored, lacking iridescent microtomentum, ocellar triangle largely and fronto-orbits whitish tan to tan, ocellar triangle with anteromedial, narrow, slightly oval darkened area, triangle broadly extended to anterior margin, parafrons grayish charcoal. Antenna largely yellow, only dorsum of basal flagellomere slightly darkened. Face narrowed at midheight, mostly unicolorous, whitish gray to blackish gray except for mediovertical brown vitta; parafacial creamy white. Gena relatively short, gena-to-eye ratio 0.10. *Thorax*: Mesonotum with 7 brown vittae, including a medial vitta along acrostichal area (Fig. 57); presutural supra-alar seta well developed. Wing conspicuously patterned with distinct brown spots (Fig. 58); vein R_2+3_ distinctly angulate subapically, apices angled toward costa; at vertex of angle also bearing a stump vein, another stump vein near middle; costal vein ratio 0.67–0.68; M vein ratio 0.66–0.71. Femora brownish black; forefemur with 4–5 stout, peg-like setae on apical third along posteroventral margin; tibiae largely brownish black, apices yellow; tarsi yellow.

**Figures 55–58.**
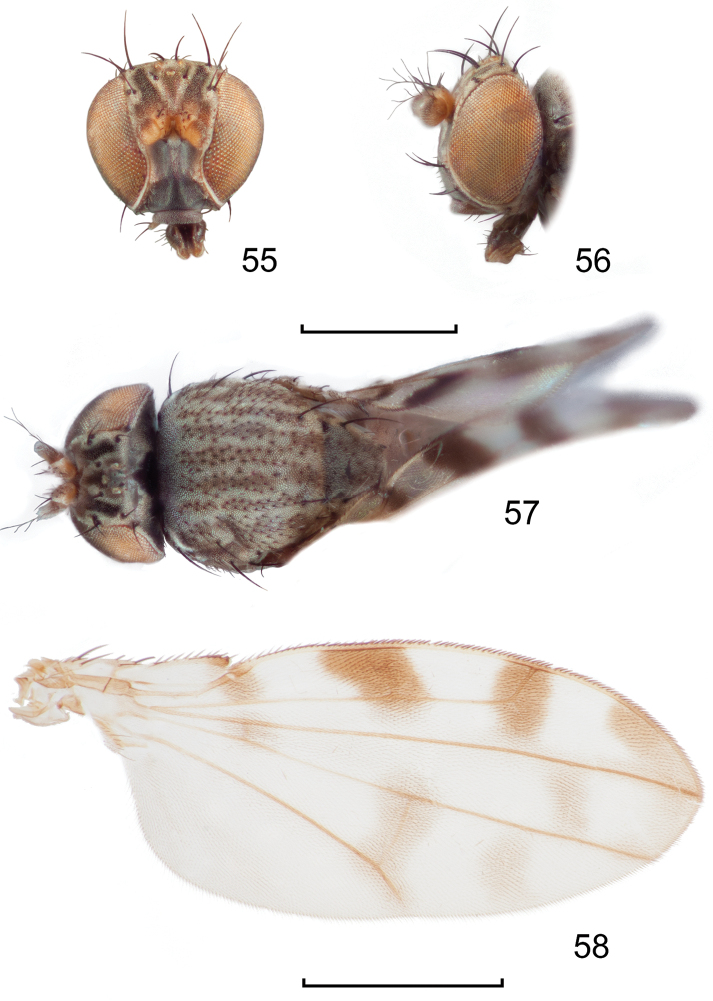
*Lamproclasiopa balsamae* (Cresson). (Costa Rica. San José: Pedregoso) **55** head, anterior view **56** same, lateral view **57** thorax, dorsal view **58** Wing. Scale bar = 0.5 mm.

##### Type material.

The holotype female of *Ditrichophora balsamae* Cresson is labeled “Puerto Castilla B. F. Hond. 6-V-26. R. H. Painter, Co [“B. F.” handwritten /TYPE No. 6365 Ditrichophora BALSAMAE E T Cresson, Jr. [red; “6365 Ditrichophora BALSAMAE” handwritten]/1182.” The holotype is double mounted (minuten pin in a block of fine foam), is in excellent condition, and is deposited in the ANSP (6365).

##### Type locality.

Honduras. Colón: Puerto Castilla (16°0.5'N, 85°57.7'W).

##### Other specimens examined.

BRAZIL. **Rio de Janeiro**: Gavea (22°58.6'S, 43°13.7'W), Mar 1929, H. Souza Lopes (6♀; IOC).

COSTA RICA. **San José**: Pedregoso (9° 22.45'N, 83° 43.2'W), D. L. Rounds (1♀; USNM).

EL SALVADOR. **La Liberdad**: Santa Tecla (12 km NW; 13°45.1'N, 89°22.1'W), Oct 1953, W. B. Heed (1♀; USNM).

##### Distribution

**Figure 59.**
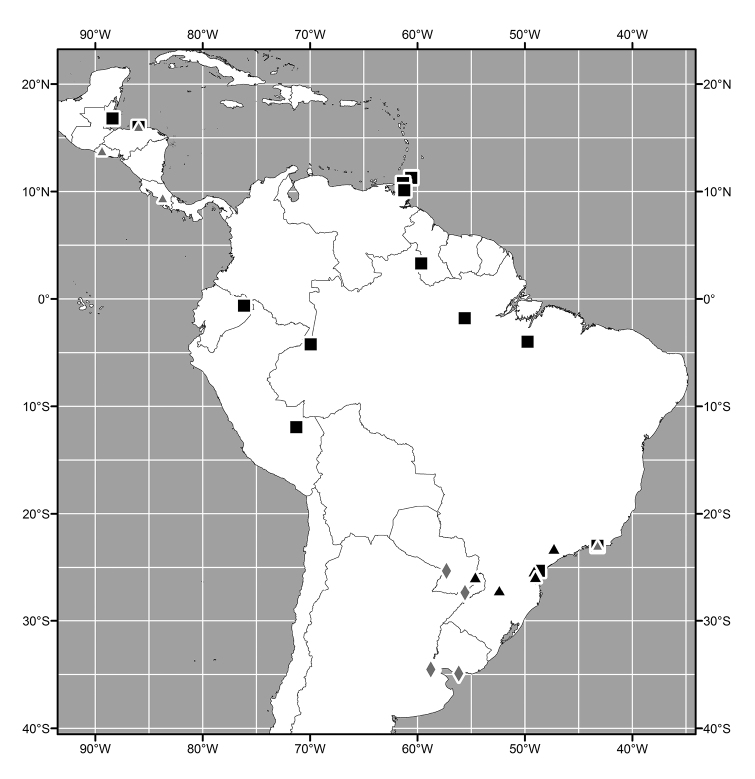
Distribution map of *Lamproclasiopa balsamae* (▲); *Lamproclasiopa bisetulosa* (♦); *Lamproclasiopa mancha* sp. n. (▲); *Lamproclasiopa painteri* (■).

(Fig. 59). *Neotropical*: Brazil (Rio de Janeiro), Costa Rica (San José), El Salvador (La Liberdad), Honduras (Colón).

##### Remarks.

Although very similar and apparently closely related to *Lamproclasiopa painteri*, this species is distinguished from congeners by the number and entirety of the mesonotal stripes. There are seven longitudinal vittae, including a medial vitta in the acrostichal area.

A male of this species is unavailable, and thus, our diagnosis is incomplete for structures of the male terminalia.

#### Lamproclasiopa mancha sp. n.

Taxon classificationAnimaliaDipteraEphydridae

http://www.zoobank.org/A9F90349-9066-4D6B-8D7B-7AF6C13B5DEF

Figs 59 , 60–62 , 63–66

##### Diagnosis.

This species is distinguished from other congeners by the following combination of characters: Small to moderately small shore-flies, body length 1.55–2.10 mm. *Head*: Frons mostly yellowish to golden tan, especially outline of mesofrons and fronto-orbits, anterior portion immediately dorsad of antennae yellowish orange, microtomentum denser and whiter at base of fronto-orbital setae. Antenna yellow to yellowish orange; basal flagellomere slightly brownish dorsally. Face black but completely to mostly silvery white microtomentose, often with medial, darker stripe with microtomentum thinner, otherwise lacking prominent, vertical stripes; bearing 2 larger facial setae, dorsal seta at about midfacial height, dorsomesoclinate; ventral seta just dorsad of epistomal margin, slightly dorsoclinate; parafacial silvery to creamy white; gena very short. Gena-to-eye ratio 0.05–0.07. *Thorax*: Mesonotum uniformly tannish to golden tan microtomentose; presutural supra-alar seta well developed; pleural area very sparsely microtomentose, mostly dark brown, partially subshiny, contrasted with densely microtomentose mesonotum. Wing hyaline except for conspicuous darkened clouds over crossveins r-m and especially over dm-cu (Fig. 62); vein R_2+3_ with apex more abruptly curved toward costa; costal vein ratio 0.51–0.60; M vein ratio 0.59–0.65. Femora mostly black; forefemur with 4–5 stout, peg-like setae on apical third along posteroventral margin; tibiae mostly brownish black, apices yellow; tarsi yellow. *Abdomen*: Generally black, mostly subshiny to shiny, dorsum of tergites very sparsely and finely microtomentose, faintly whitish gray. Male terminalia (Figs 63–66): Epandrium in posterior view (Fig. 63) elongate, inverted U-shaped, dorsal arch narrow, becoming wider ventrally, ventral margin bearing loose cluster of longer setulae, in lateral view widest subventrally, ventral margin more narrowly rounded; cercus in posterior hemispherical, not fused with ventral margin of cercal cavity, with somewhat evenly scattered setulae, those toward ventral margin longer; gonite in lateral view (Fig. 66) somewhat rod-like, shallowly curved, both ends tapered, in ventral view (Fig. 65) knife-like, medial end blade-like, lateral extension narrow with apical portion curved and pointed; aedeagus in lateral view (Fig. 66) as an angulate funnel, comparatively wide basally, thereafter apically angles posteroventrally, tapered to narrowly pointed apex, in ventral view (Fig. 65) an elongate, narrow funnel; phallapodeme in lateral view (Fig. 66) irregularly Y-shaped, keel long, narrow, linear; hypandrium in ventral view (Fig. 65) as 2 narrow, parallel sided, thin sclerites, with posterior connection, in lateral view (Fig. 66) an elongate, slender, rod-like structure, bulbous posteriorly, shallowly bifurcate anteriorly.

**Figures 60–62.**
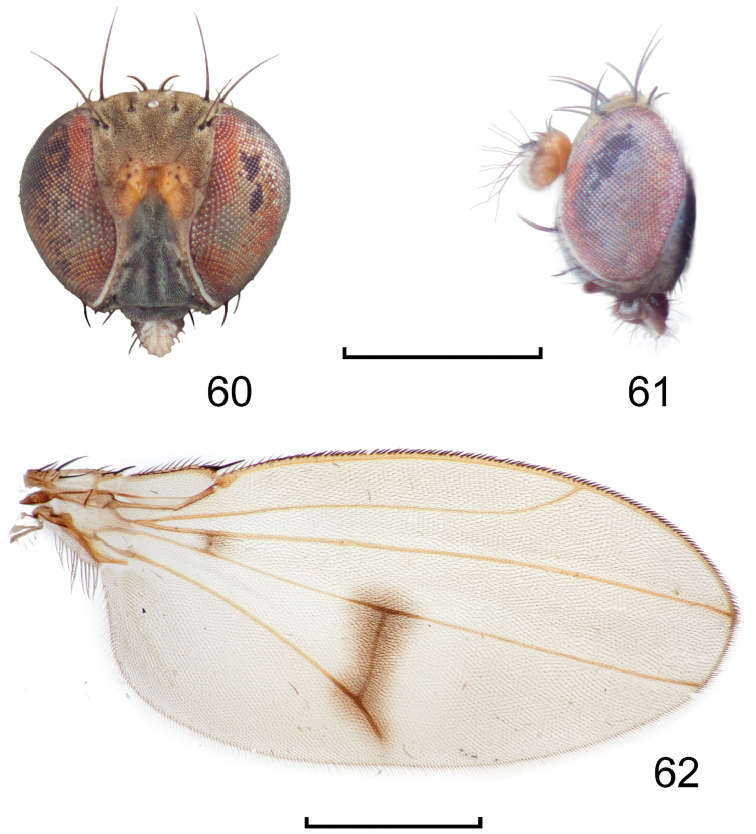
*Lamproclasiopa mancha* sp. n. (Brazil. Paraná: Curitiba) **60** head, anterior view **61** same, lateral view **62** Wing. Scale bar = 0.5 mm

**Figures 63–66.**
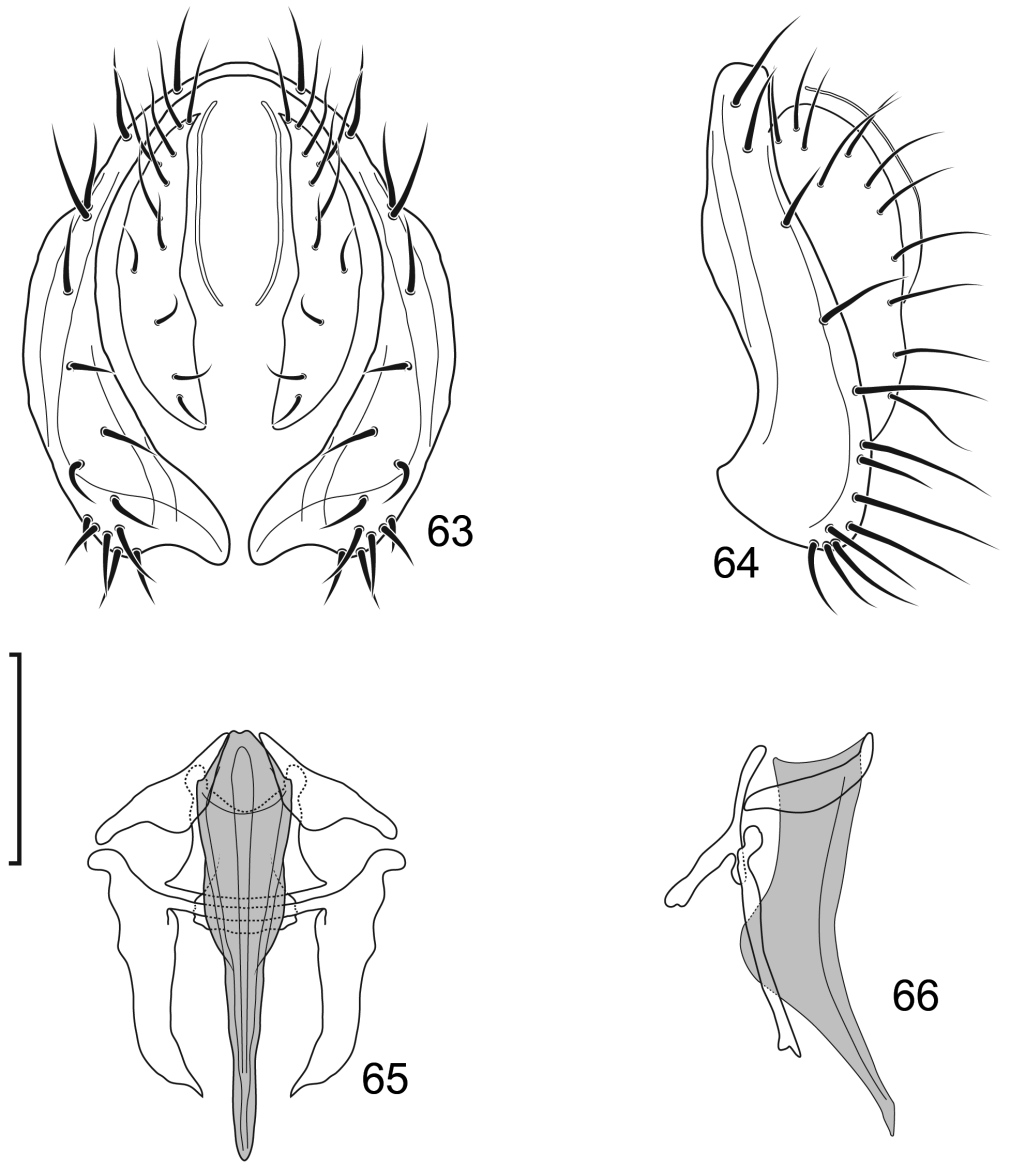
*Lamproclasiopa mancha* sp. n. (Brazil. Paraná: Curitiba) **63** epandrium and cerci, posterior view **64** same, lateral view **65** internal structures of male terminalia (aedeagus [shaded], phallapodeme, gonite, hypandrium), ventral view **66** same, lateral view. Scale bar = 0.1 mm.

##### Type material.

The holotype male of *Lamproclasiopa mancha* is labeled “**BRAZIL.** Paraná: Curitiba, UFPR [Universidade Federal do Paraná, Reserva Biológica] (25°26.9'S, 49°14'W; 915 m),6 Feb2010[,] D. & W. N. Mathis/USNM ENT 00118310 [plastic bar code label]/HOLOTYPE ♂ *Lamproclasiopa mancha* Costa, Mathis & Marinoni DZUP [red].” The holotype is double mounted (minuten pin in a block of plastic), is in excellent condition, and is deposited in DZUP. Twenty-nine paratypes (24♂, 5♀; DZUP, USNM) bear the same locality data as the holotype, with dates from 9 Dec 2009–13 Feb 2010.

##### Type locality.

Brazil. Paraná: Curitiba, Universidade Federal do Paraná, Reserva Biológica (25°26.9'S, 49°14'W; 915 m).

##### Other specimens examined.

ARGENTINA. **Misiones**: Puerto Bemberg (25°55'S, 54°37'W), 13 Oct 1950, L. C. Shum (1♀; USNM).

BRAZIL. **Paraná**: Colombo (Santa Monica tennis club; 25°23.1'S, 49°08.8'W; 860 m), 18 Mar 2015, Daniel N. R. Costa (1♂; DZUP); Curitiba, Universidade Federal do Paraná, Reserva Biológica (25°26.9'S, 49°14'W; 915 m), 1–4 Feb 2010, 13–17 Out 2014, Daniel N. R. Costa (13♂, 4♀; DZUP); Morro do Araçatuba (Município de Tijucas do Sul; 25°53.8'S, 49°01.2'W; 910 m), 27 Feb 2015, W. N. Mathis (1♀; DZUP). **Santa Catarina**: Nova Teutônia (27°11'S, 52°23'W; 3–500 m), Jul-Nov 1970, 1971, F. Plaumann (9♂, 4♀; MZUSP). **São Paulo**: Itú (23°15.9'S, 47°17.9'W), Sep 1960, M. A. V. D’Andretta (8♂, 2♀; MZUSP).

##### Distribution

(Fig. 59). Neotropical: Argentina (Misiones), Brazil (Paraná, Santa Catarina, São Paulo).

##### Etymology.

The species epithet, *mancha*, is the Portuguese word for a stain and refers to the darkened clouds over crossveins r-m and dm-cu, diagnostic of this species.

##### Remarks.

This species is easily distinguished from congeners by the wing pattern. The wing is mostly hyaline except for darkened clouds over crossveins r-m and especially over dm-cu; and vein R_2+3_ has the apex more abruptly curved toward the costa. Sometimes the darkened spots over the crossveins are slightly faded.

#### Lamproclasiopa painteri

Taxon classificationAnimaliaDipteraEphydridae

(Cresson)

Figs 59 , 67–70 , 71–74

Ditrichophora painteri Cresson 1930: 76.
Discocerina (Basila) painteri . Cresson 1946: 149 [generic combination]. Wirth 1968: 7 [Neotropical catalog]. Mathis and Zatwarnicki 1995: 165 [world catalog].
Lamproclasiopa balsamae , of authors, not Cresson [misidentification]. Zatwarnicki and Mathis 2001: 36 [generic combination].
Lamproclasiopa painteri . Zatwarnicki and Mathis 2001: 39 [generic combination].

##### Diagnosis.

This species is easily distinguished from congeners by the following combination of characters: Small shore flies, body length 1.15–1.80 mm. *Head*: Frons bi- or tricolored, lacking iridescent microtomentum, ocellar triangle largely and fronto-orbits whitish tan to tan, ocellar triangle with anteromedial, narrow, slightly oval darkened area, triangle broadly extended to anterior margin, parafrons grayish charcoal. Antenna largely yellow, only dorsum of basal flagellomere slightly darkened. Face narrowed at midheight, mostly unicolorous, whitish gray to blackish gray except for mediovertical brown vitta; parafacial creamy white. Gena relatively short, gena-to-eye ratio 0.08–0.09. *Thorax*: Mesonotum with 4 elongate, mostly separated spots (Fig. 69); presutural supra-alar seta well developed. Wing conspicuously patterned with distinct brown spots (Fig. 70); vein R_2+3_ distinctly angulate subapically, apices angled toward costa; at vertex of angle also bearing a stump vein, another stump vein near middle; costal vein ratio 0.76–0.87; M vein ratio 0.66–0.75. Femora brownish black; forefemur with 4–5 stout, peg-like setae on apical third along posteroventral margin; tibiae largely brownish black, apices yellow; tarsi yellow. *Abdomen*: Tergite 5 of male truncate posteriorly. Male terminalia (Figs 71–74): Epandrium in posterior (Fig. 71) view roundly U-shaped, bluntly oval, narrower dorsally and ventrally, slightly wider at midheight, lateral arm becoming wider ventrally, curved medially ventral margin ventromedial gap V-shaped, ventral angle bearing loosely clustered setulae; cercus hemispherical, pointed dorsomedially, more setulose dorsally, medial margin straight; gonite in lateral view (Fig. 74) robustly rod-like, shallowly curved toward aedeagal base, shaped like a banana, in ventral view shallowly curved with extension toward aedeagal base narrow, thumb-like, thereafter moderately wide, widest subapically; aedeagus in lateral view (Fig. 74) comparatively narrowly truncate basally, thereafter expanded, widest sub-basally, thereafter tapered to rounded apex, apex with short, recurved anterior point, in ventral view (Fig. 73) as an elongate, shallowly rounded, narrowed medially, basal margin somewhat truncate with shallow, medial emargination, apical margin tapered to angulate, rounded apex; phallapodeme in lateral view (Fig. 74) Y-shaped with one arm of Y a short, irregularly narrow keel, keel irregularly tapered, pointed apically; hypandrium in lateral view (Fig. 74) narrowed basally, apical ½-2/3 wider, narrowly rectangular, rounded anteriorly, in ventral view as a very broad, short H with short arms, anterior emargination broadly V-shaped, posterior emargination very broadly and shallowly U-shaped.

**Figures 67–70.**
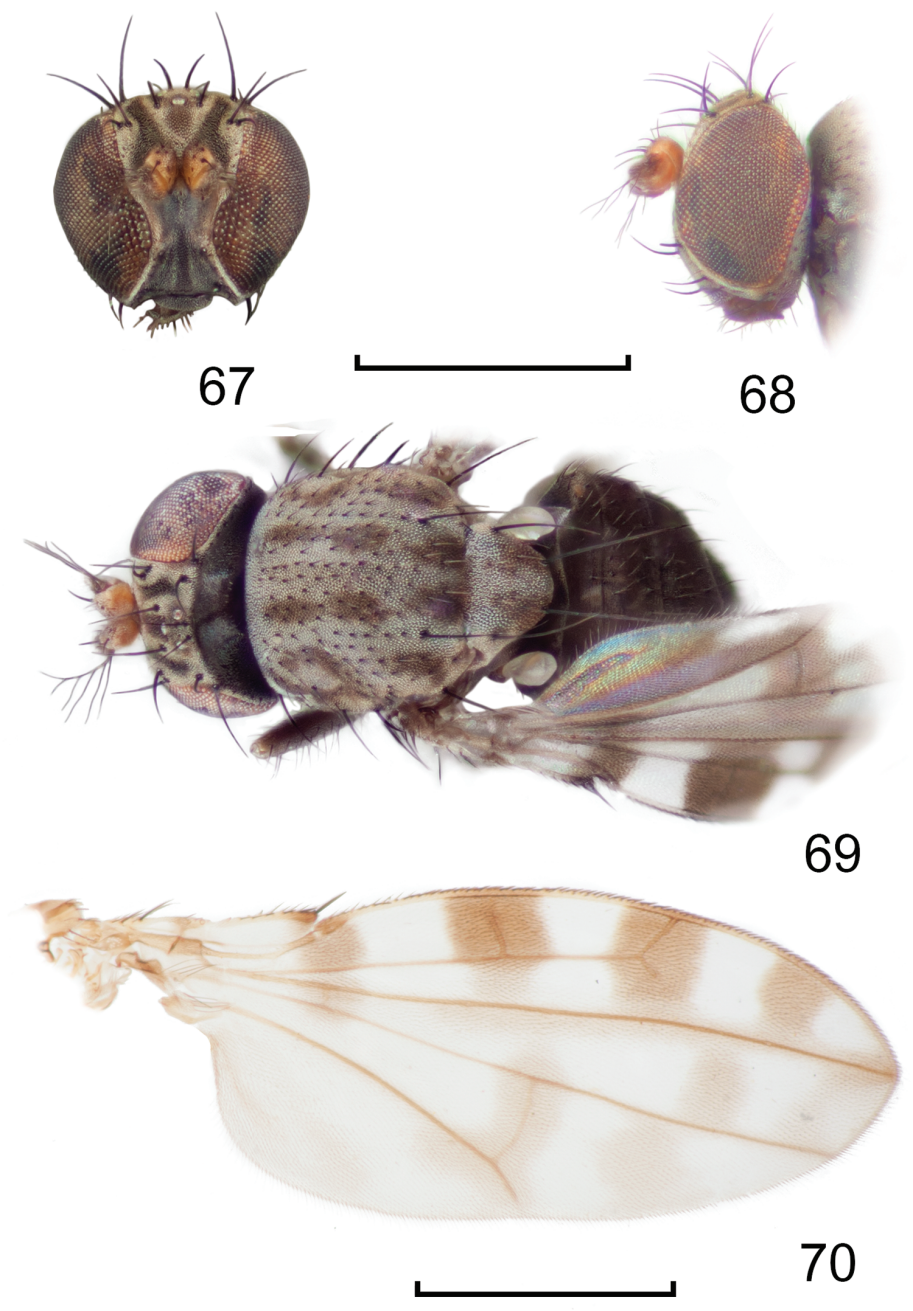
*Lamproclasiopa painteri* (Cresson). (Peru. Madre de Dios: Río Manu, Pakitza) **67** head, anterior view **68** same, lateral view **69** thorax, dorsal view **70** Wing. Scale bar = 0.5 mm.

**Figures 71–74.**
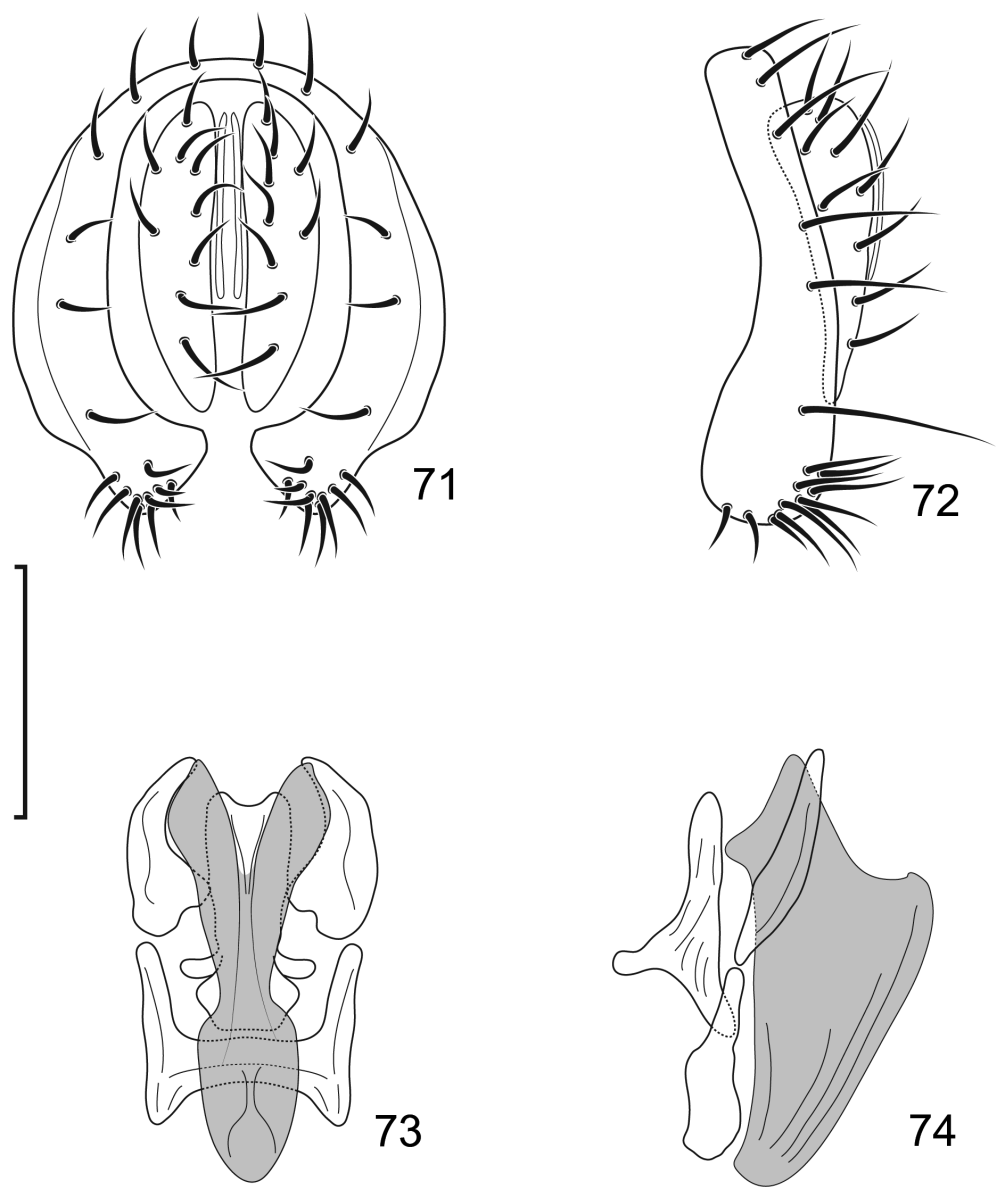
*Lamproclasiopa painteri* (Cresson). (Peru. Madre de Dios: Río Manu, Pakitza) **71** epandrium and cerci, posterior view **72** same, lateral view **73** internal structures of male terminalia (aedeagus [shaded], phallapodeme, gonite, hypandrium), ventral view **74** same, lateral view. Scale bar = 0.1 mm.

##### Type material.

The holotype female of *Ditrichophora painteri* Cresson is labeled “Puerto Castilla B. F. Hond. 6-V-26 [6 May 1926] R. H. Painter, Co [“B. F.” handwritten]/TYPE No. 6366 Ditrichophora PAINTERI E.T.Cresson, Jr, [red; “6366 Ditrichophora PAINTERI” handwritten]/1182.” The holotype is double mounted (minuten pin in a block of fine foam), is in excellent condition, and is deposited in the ANSP (6366). Three paratypes (2♂, 1♀; ANSP) bear the same locality label as the holotype.

##### Type locality.

Honduras. Colón: Puerto Castilla (16°0.5'N, 85°57.7'W).

##### Other specimens examined.

BELIZE. **Stann Creek**: Cockscomb Basin Wildlife Sanctuary (16°47'N, 88’30'W), 5–6 Apr 1993, W. N. Mathis (9♂, 8♀; USNM); Maya Center: Cabbage Haul Creek (16°48'N, 88°23'W), 3 Apr 1993, W. N. Mathis (1♂, 1♀; USNM).

BRAZIL. **Amazonas**: Marco (near Leticia=Tabatinga; 04°13.9'S, 69°56'W), Aug 1960, H. L. Carson, W. B. Heed (1♂; USNM). **Pará**: Oriximiná-Obidos, estrada (01°47.7'S, 55°36'W), Nov 1969 (1♂; MZUSP); Tucuruí, Morro do Senador (03°59.4'S, 49°44.8'W), Dec 2001, J. A. Rafael, J. Vidal (1♀; INPA). **Paraná**: Antonina, Reserva Natural Rio Cachoeira (25°19'S, 48°41,6'W), 8 Feb 2010, D. N. R. Costa (3♂; DZUP). **Rio de Janeiro**: Gávea (22°59.2'S, 43°14.7'W), 31 Mar 1937, H. de Souza Lopes (5♂, 1♀; ANSP, MNRJ + 2♂; NMNH).

ECUADOR. **Orellana**: Río Tiputini (0°38.2'S, 76°8.9'W), 12–26 Aug 1999, W. N. Mathis, A. Baptista, M. Kotrba (5♀; USNM).

GUYANA. Moco-Moco (30 km E Lethem in Kanuku Mountains; 3°18.2'N, 59°39.0'W), 29 Apr 1995, W. N. Mathis (9♂, 3♀; USNM).

PANAMA. **Colon**: Juan Gallegos (9°37'N, 79°34'W), 10 Jul 1982, R. B. Kimsey (1♀; USNM).

PERU. **Madre de Dios**: Río Manu, Pakitza (11°56.6'S, 71°16.9'W; 250 m), 9–23 Sep 1988, W. N. Mathis (27♂, 20♀; USNM).

TRINIDAD and TOBAGO. Tobago. **St. John**: Charlotteville (5 km S; 11°18.9'N, 60°34.5'W), Hermitage River and beach, 22 Apr-11 Jun 1993, 1994, D. and W. N. Mathis (5♂, 5♀; USNM); Parlatuvier (creek; 11°17.9'N, 60°35'W), 20 Apr-14 Jun 1993, 1994, W. N. Mathis (2♂, 1♀; USNM). **St. Paul**: Argyle Falls (11°15'N, 60°35'W), 21 Apr 1994, W. N. Mathis (1♂; USNM); Roxborough (6 km NNW; 11°16'N, 60°35.4'W), 20 Apr 1994, W. N. Mathis (13♂, 14♀; USNM). Trinidad. **Caroni**: Talparo (2 km N, 10°31'N, 61°17'W), 22 Jun 1993, W. N. Mathis (1♂; USNM). **St. George**: Filette (1 km SE; 10°47'N, 61°21'W), Yarra River, 25 Jun 1993; W. N. Mathis (2♀; USNM). **Victoria**: Basse Terre (7 km E; 10°07'N, 61°14'W), 27 Jun 1993, W. N. Mathis (3♂, 3♀; USNM).

VENEZUELA. Caife, Jan 1943, P. J. Anduze (2♀; USNM).

##### Distribution

(Fig. 59). Neotropical: Belize (Stann Creek), Brazil (Amazonas, Pará, Paraná, Rio de Janeiro), Ecuador (Orellana), Guyana, Honduras (Colón), Peru (Madre de Dios), Trinidad and Tobago, Venezuela.

##### Remarks.

Although similar to *Lamproclasiopa balsamae*, this species is distinguished from that species and all other congeners by having a conspicuously spotted wing, as in *Lamproclasiopa balsamae*, and by having the mesonotum with four stripes, each as a short series of two to four more or less elongated spots. In *Lamproclasiopa balsamae* there are seven distinct and complete or nearly complete stripes. Structures of the male terminalia also distinguish this species.

The illustration of *Lamproclasiopa balsamae* that Mathis and Zatwarnicki (2001) published is actually that of *Lamproclasiopa painteri*.

The locality of the specimen from Venezuela is a mystery to us. We have checked and rechecked the spelling on the label, “Caife,” but have been unable to locate this name on maps or gazetteers. Perhaps it is a misspelling.

### The *nana* group (*Lamproclasiopa nana*)

**Diagnosis.** Body with extensive surfaces sparsely to densely microtomentose. *Head*: Frons and face distinctly two-toned; antenna yellow; gena relatively short (gena-to-eye ratio 0.06–0.10); genal/postgenal margin rounded. *Thorax*: Presutural supra-alar seta well developed. Wing generally hyaline to faintly infumate; vein R_2+3_ curved gently apically, not angulate subapically nor bearing a subapical stump vein. Forefemur with 4–5 stout, peg-like setae on apical third along posteroventral margin; tibiae entirely black. *Abdomen*: Male terminalia: Dorsal epandrial margin interrupted, each lateral arm of the epandrium robustly developed; hypandrium in ventral view rectangular.

**Remarks.** This species group, comprising a single species, is very distinctive among all congeners and is easily recognized. Although distinctive, it is apparently related to the *furvitibia* group.

#### Lamproclasiopa nana

Taxon classificationAnimaliaDipteraEphydridae

(Williston)

**Figures 75–76.**
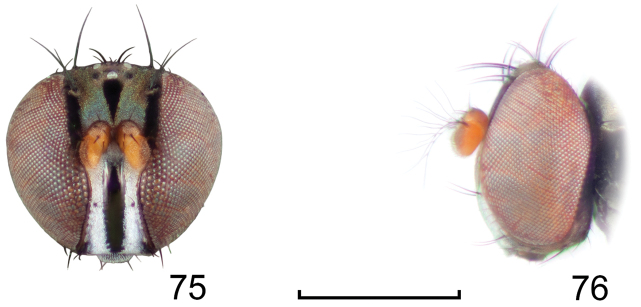
*Lamproclasiopa nana* (Williston). (Brazil. Paraná: Curitiba) **75** head, anterior view **76** same, lateral view. Scale bar = 0.5 mm.

**Figures 77–80.**
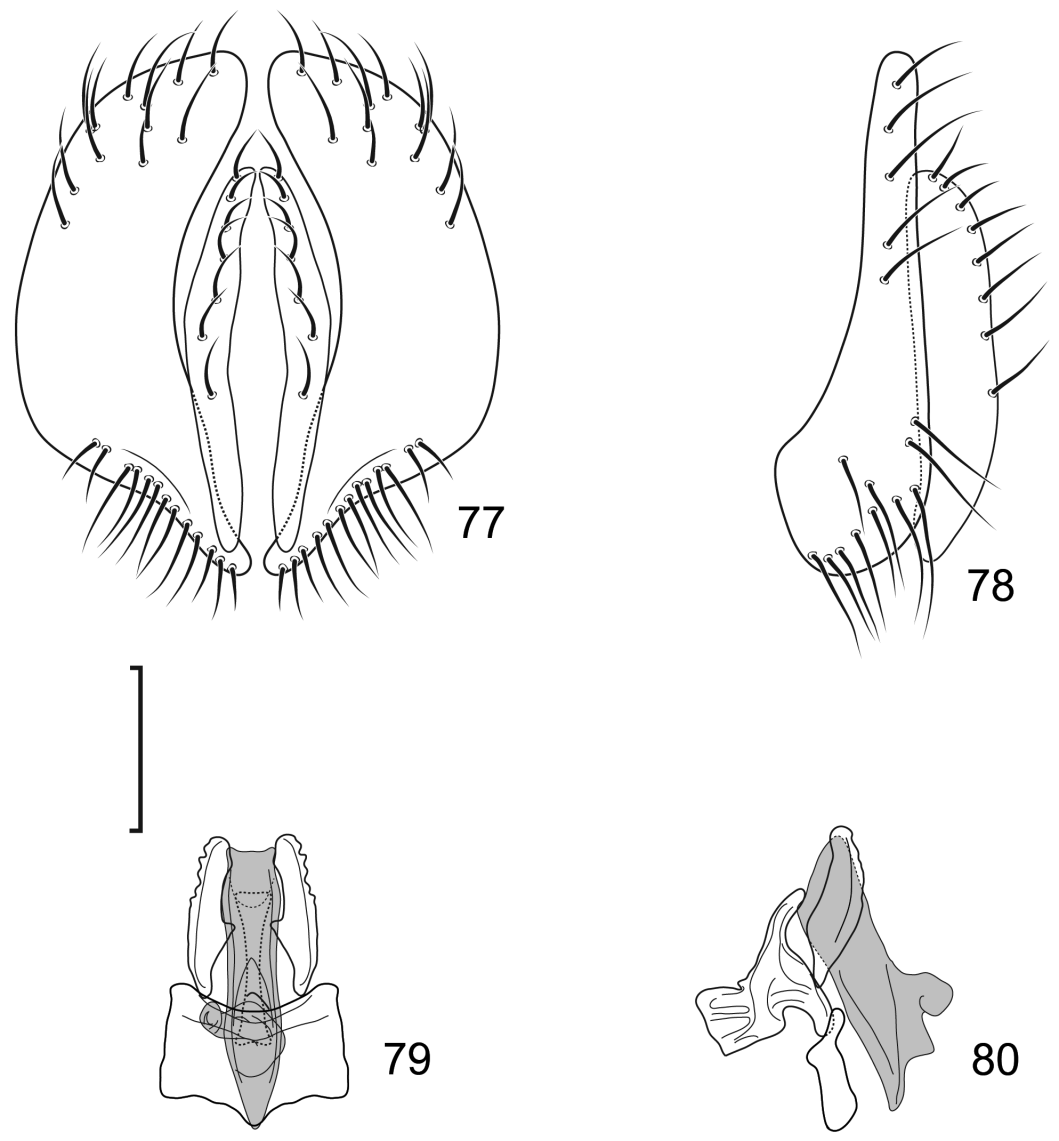
*Lamproclasiopa nana* (Williston). (Brazil. Paraná: Curitiba) **77** epandrium and cerci, posterior view **78** same, lateral view **79** internal structures of male terminalia (aedeagus [shaded], phallapodeme, gonite, hypandrium), ventral view **80** same, lateral view. Scale bar = 0.1 mm.

**Figure 81.**
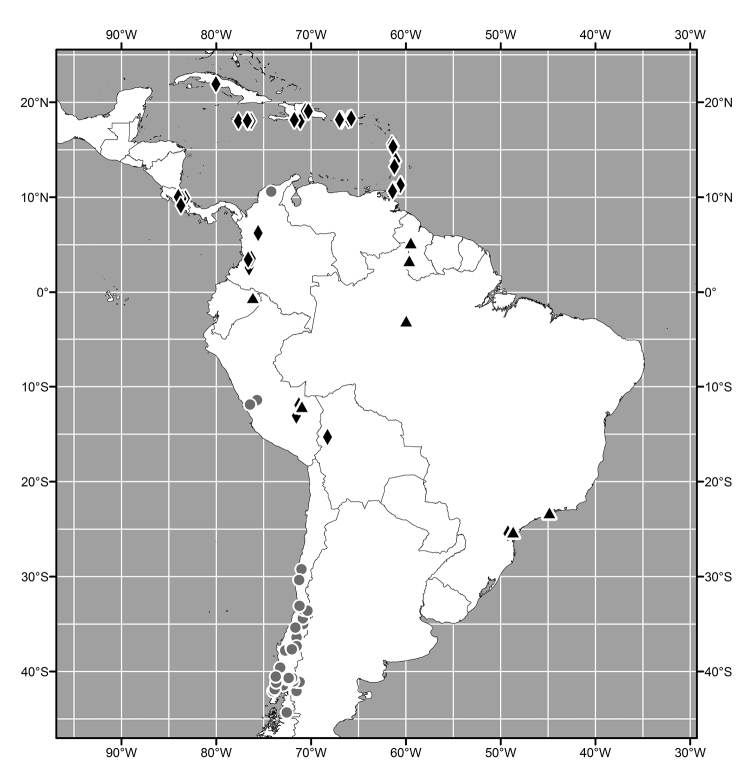
Distribution map of *Lamproclasiopa aracataca* (●); *Lamproclasiopa nana* (♦); *Lamproclasiopa triangularis* sp. n. (▲).

Figs 75–76 , 77–80 , 81

Discocerina nana Williston 1896: 396.
Ditrichophora nana . Cresson 1924: 159 [generic combination].
Discocerina (Basila) nana . Cresson 1946: 149 [review]. Wirth 1968: 7 [Neotropical catalog]. Mathis and Edmiston 1991: 824 [review of Williston’s St. Vincent species]. Mathis and Zatwarnicki 1995: 165 [world catalog].
Lamproclasiopa nana . Zatwarnicki and Mathis 2001: 39 [generic combination]. Perez-Gelabert 2008: 177 [list, Hispaniola].

##### Diagnosis.

This species is distinguished from congeners by the following combination of characters: Small to moderately small shore-flies, body length 1.60-2.40 mm. *Head*: Frons distinctly two toned, fronto-orbits and narrow, medial triangular area densely microtomentose, velvety black; mesofrons other than narrow, medial triangle, seriaceus, bronzish to copperish gray to blue. Antenna yellow, some specimens slightly black anterodorsally. Facial series with 2 setae on each side; face also distinctly two toned, a narrow, bare, shiny, vertical stripe that is bordered laterally by dense, palely golden-white microtomentum; parafacial very narrow, densely silvery white microtomentose. Gena relatively short, gena-to-eye ratio 0.06–0.10. *Thorax*: Mesonotum almost uniformly colored and invested with light dusting of microtomentum, lacking distinct stripes or isolated spots; presutural supra-alar seta well developed. Wing hyaline; costal vein ratio 0.71–0.90; M vein ratio 0.57–0.62. Coxae, femora and tibiae black, tarsi yellow; forefemur with 4–5 stout, peg-like setae on apical third along posteroventral margin. *Abdomen*: Tergites shiny black. Male terminalia (Figs 77–80): Epandrium in posterior view (Fig. 77) disconnected dorsally, each lateral portion vertically elongate and robustly developed, height nearly 3× width, ventral portion on each side tapered laterally to medial, pointed apex, sloping ventral margin more conspicuously setulose, setulae mostly straight, also setulose dorsally, these setulae shallowly curved; cerci in posterior view (Fig. 77) very narrowly developed, elongate, bearing more setulae dorsally, linear; gonite in lateral view (Fig. 80) shallowly and broadly zigzagged, each apex narrowed, in ventral (Fig. 79) view linear, rod-like; aedeagus in lateral view (Fig. 80) generally rectangular, more broadly developed apically, apical margin slightly emarginate, with an elongate, narrow membranous extension, in ventral view an elongate, narrow, rod-like structure, narrowly truncate basally, apex roundly tapered to narrow point; phallapodeme in lateral view (Fig. 80) with distinctive, relatively broad keel, each extended process abruptly narrowed, in ventral view elongate with each end wider and with short, midheight papilla-like extensions laterally; hypandrium in lateral view (Fig. 80) mostly rectangular with posterior, narrow process angled toward aedeagus, in ventral view (Fig. 79) rectangular, wider than long, anterior margin produced to form a short, medial point, posterior margin very shallowly concave.

##### Type material.

The lectotype male of *Discocerina nana* Williston (designated by Mathis and Edmiston 1991: 824) is labeled “Co-type [circular label with a yellow border]/1000 feet/[a black, square label]/St. Vincent, W.I. H. H. Smith./W.Indies. 1907–66./Discocerina nana Will. [handwritten, two red submarginal borders]/LECTOTYPE Discocerina nana Will. ♂ By W.N.Mathis [handwritten except for “LECTOTYPE” and “By”, black sub-border].” the lectotype is double mounted (pin in a rectangular piece of cardboard), is in good condition, and is deposited in the BMNH. There are also eight paralectotypes as follows: BMNH (5♂, 1♀), AMNH (1♂, 1♀). Williston, in the original description, noted that the type series included “Numerous specimens.”

##### Type locality.

West Indies. St. Vincent (13°15'N, 61°11'W).

##### Other specimens examined.

BOLIVIA. **La Paz**: Guanay (22 km SE; 15°17.8'S, 68°15.6'W; 540 m), 17 Mar 2001, W. N. Mathis (1♂; USNM).

BRAZIL. **Paraná**: Colombo (Santa Monica tennis club; 25°23.1'S, 49°08.8'W; 860 m), 18 Mar 2015, Daniel N. R. Costa (1♂; DZUP); Curitiba, Parque Iguaçú (25°33.4'S, 49°13.6'W; 880 m), 20–31 Aug 2012, Daniel. N. R. Costa (5♂; DZUP); Curitiba, Universidade Federal do Paraná, Reserva Biológica (25°26.9'S, 49°14'W; 915 m), 6–13 Feb 2010, D. and W. N. Mathis (2♂, 1♀; DZUP, USNM).

COLOMBIA. **Antioquia**: Medellín (6°14.1'N, 75°34.5'W; coffee finca; 1525 m), Nov 1955, W. B. Heed (2♂; USNM). **Cauca**: Popayán (30 km N; 2°36'N, 76°31.6'W), M. R. Wheeler (1♂; USNM). **Valle de Cauca**: Palmira (3°32.4'N, 76°18.4'W), Mar 1958, M. R. Wheeler (1♂, 1♀; USNM). Cali (10 km W; 3°26.7'N, 76°37.3'W; 1640 m), 20 Mar 1955, E. I. Schlinger, E. S. Ross (1♂; USNM).

COSTA RICA. **Cartago**: Juan Viñas (09°53.6'N, 83°15.3'W), 28 Apr 1910, P. P. Calvert (1♀; ANSP); La Suiza (9°51.5'N, 83°37.5'W), 6 May 1926, P. Schild (3♀; ANSP, USNM). **Puntarenas**: Pedregosa (09°08.4'N, 83°43.5'W), D. L. Rounds (1♀; ANSP). San José: Moravia, Zurquí de Moravia (Tower path; 10°2.8'N, 84°0.6'W, 1600 m), 6 Sep-29 Dec 2012, 2013, Project ZADBI (2♂, 5♀; MNCR-A).

CUBA. **Sancti Spiritus**: Topes de Collantes (21°55.2'N, 80°02'W; 350 m), 10 Dec 1994, W. N. Mathis (1♂; USNM).

DOMINICA. Antrim Valley (15°20.7'N, 61°22.2'W; 305 m), 17 Mar 1956, J. F. G. Clarke (1♂, 3♀; USNM). Clarke Hall (15°24.5'N, 61°23.7'W), 16 Jan-21–31 Mar 1965, W. W. Wirth (1♂, 1♀; USNM). Fond Figues River (15°24'N, 61°18'W; 122 m), 30 Jan-22 Apr 1965, 1966, R. J. Gagné, W. W. Wirth (9♀, 2♀; USNM). G’leau Gommier near Belles (15°25.4'N, 61°20.4'W), 17 Mar 1956, J. F. G. Clarke (2♂, 6♀; USNM). Layou River (15°25.8'N, 61°21.4'W; 5 km E), 23 Mar 1989, W. N. Mathis (1♂; USNM). Pont Cassé (15°22.7'N, 61°20.6'W), 12 Feb-18 Jun 1965, 1991, D. and W. N. Mathis, W. W. Wirth (11♂, 2♀; USNM). South Chiltern Estate (15°14.8'N, 61°21.8'W), 2 Feb 1965, W. W. Wirth (1♀; USNM). Toucari (2 km S; 15°36.6'N, 61°27.8'W), 21 Mar 1989, W. N. Mathis (2♂, 1♀; USNM). Trafalgar Falls (15°19'N, 61°21'W; 365 m), 6 Mar-19 Jun 1965, 1991, R. J. Gagné, D. and W. N. Mathis, W. W. Wirth (4♂, 4♀; USNM).

DOMINICAN REPUBLIC. **Barahona**: San Rafael (18°01.9'N, 71°08.4'W), 22 Mar 1999, W. N. Mathis (3♂, 3♀; USNM). **La Vega**: Jarabacoa (5 km S; 19°05.8'N, 70°36.5'W; 640 m), 8-20 May 1995, W. N. Mathis (9♂, 2♀; USNM); Paso de la Vaca, road from Monseñor Nouel to Constanza (19°04'N, 70°16.9'W; 1500 m), 27 Dec 1955, J. Maldonado Capriles (1♂, 1♀; USNM). **Pedernales**: Rio Mulito (21 km N Pedernales; 18°09.3'N, 71°45.6'W; 270 m), 18-20 Mar 1999, W. N. Mathis (1♀; USNM). **San Jose de Ocoa**: San Jose de Ocoa (30.6 km N; 18°49.5'N, 70°30'W; 914 m), 30 Jul 1991, D. Grimaldi, J. Stark (2♂; AMNH).

JAMAICA. **Portland**: Reach Falls (18°01.8'N, 76°18.7'W), 15 May 1996, D. and W. N. Mathis, H. Williams (1♂; USNM); Reach Falls, Drivers River (18°01.9'N, 76°18.7'W; 70 m), 25 Apr 2000, W. N. Mathis (1♀; USNM); Section (0.5 km E; 18°05.2'N, 76°43.9'W; 1020 m), 28 Apr 2000, W. N. Mathis (9♂, 4♀; USNM). **St. Andrew**: Cinchona (18°04.4'N, 76°39.3'W; 1400 m), 29 Apr 2000, W. N. Mathis (3♀; USNM); Hardwar Gap (18°04.2'N, 76°44'W), 17 May 1996, D. and W. N. Mathis, H. Williams (2♂; USNM); Hardwar Gap (18°04.2'N, 76°44'W; 1170 m), 27 Apr 2000, W. N. Mathis (4♂, 1♀; USNM); Hollywell (18°05.2'N, 76°43.9'W; 1100 m); 28 Apr 2000, W. N. Mathis (7♂; USNM); Mavis Bank (near coffee factory; 18°01.4'N, 76°39.7'W; waterfall), 21-23 Apr 2000, W. N. Mathis (1♂; USNM); Mavis Bank (1.5 km W; 18°01.4'N, 76°39.9'W), 22 Apr 2000, W. N. Mathis (1♂, 2♀; USNM); Mavis Bank (1.7 km E; 18°02.4'N, 77°39.5'W; 575 m), Yallahs River, 21-22 Apr-1 May 2000, W. N. Mathis (1♂; USNM); Newcastle (6 km S; 18°04.3'N, 76°42.6'W; 950 m; waterfall), 30 Apr 2000, W. N. Mathis (14♂; USNM).

PERU. **Cuzco**: Paucartambo, Puente San Pedro (ca 50 km NW Pilcopata; 13°03.3'S, 71°32.8'W; 1600 m), 3 Sep 1988, W. N. Mathis (1♂, 1♀; USNM). **Madre de Dios**: Río Manu, Pakitza (11°56.6'S, 71°16.9'W; 250 m), 9-23 Sep 1988, W. N. Mathis (1♀; USNM).

PUERTO RICO. Adjuntas (near; 18°09.7'N, 66°46.6'W), 22 Sep 1995, D. and W. N. Mathis (25♂, 4♀; USNM). El Verde (18°13.5'N, 66°0-3.2'W; near biological station road), 3 Feb 1989, S.A. Marshall (1♂, 6♀; GUE). El Yungue (18°18.4'N, 65°45.6'W), 20-22 Mar 1954, J. Maldonado Capriles (1♂; USNM). Maricao, Los Viveros (18°10.5'N, 66°59.2'W), 21 Sep 1995, D. and W. N. Mathis (1♂, 1♀; USNM). Maricao (4 km WNW; 18°10.7'N, 66°59.6'W), 21 Sep 1995, D. and W. N. Mathis (1♂; USNM).

ST. LUCIA. Fond St. Jacques (13°50'N, 61°02'W), 13-14 Jun 1991, D. and W. N. Mathis (16♂, 8♀; USNM). Soufrière Botanical Garden (13°51'N, 61°04'W), 12 Jun 1991, D. and W. N. Mathis (1♂, 1♀; USNM).

ST. VINCENT. **St. Patrick**: Hermitage (6 km E Spring Village at Cumberland River; 13°14'N, 61°13.2'W; 550 m), 9 Jul 1989, M. Sorensson, B. Mårtensson (8♂, 2♀; MZLU); Hermitage (13°15'N, 61°12.9'W), 9 Sep 1997, W. N. Mathis (1♂; USNM).

TRINIDAD AND TOBAGO. Tobago. **St. John**: Charlotteville (2 km S; 11°19'N, 60°33'W), 10 Jun 1993, 1994, W. N. Mathis (1♂, 1♀; USNM); Parlatuvier (creek; 11°17.9'N, 60°35'W), 20 Apr 1994, W. N. Mathis (1♂, 1♀; USNM). **St. Paul**: Roxborough (6 km NNW; 11°16'N, 60°35.4'W), 20 Apr 1994, W. N. Mathis (8♂, 6♀; USNM); Roxborough (6.5 km N; 11°17'N, 60°35'W), 14 Jun 1993, W. N. Mathis (1♂; USNM). Trinidad. **St. George**: Marianne River (9 km S; 10°46'N, 61°18'W), 25 Jun 1993, W. N. Mathis (1♀; USNM); Mount St. Benedict (10°39'N, 61°24'W), 18-21 Jun 1993, W. N. Mathis (1♂; USNM); Mount St. Benedict (10°39'N, 61°24'W; creek near base), 19 Jun 1993, W. N. Mathis (1♂; USNM).

##### Distribution

(Fig. 81). Neotropical: Bolivia (La Paz), Brazil (Paraná), Costa Rica (Cartago), Colombia (Antioquia, Cauca, Valle de Cauca), Peru (Cuzco, Madre de Dios), Trinidad and Tobago, West Indies (Cuba, Dominica, Dominican Republic, Grenada, Jamaica, Puerto Rico, St. Lucia, St. Vincent).

##### Remarks.

This species is beautifully and strikingly colored, especially the head, and this color pattern distinguishes it from congeners. These characters, as noted in this species’ diagnosis, are as follows: Frons distinctly two toned; fronto-orbits narrow; medial triangular area densely microtomentose, velvety black; mesofrons, other than narrow, medial triangle, seriaceus, bronzish to copperish gray to blue; antenna yellow, some specimens slightly black anterodorsally. Facial series with two setae on each side; face also distinctly two toned, a narrow, bare, shiny, vertical stripe that is bordered laterally by dense, palely golden-white microtomentum; parafacial very narrow, densely silvery white microtomentose; wing hyaline.

Just as the color pattern of the head is unique among congeners, the structures of the male terminalia likewise represent a departure from the more typical pattern. This is especially evident in the shape of the epandrium, which exhibits a dorsal gap and the lateral arms are greatly thickened. Like many other species of *Lamproclasiopa*, there are loosely clustered setulae along the ventromedial margin. The cerci are slender and comparatively elongated. The internal structures are more typical, although the aedeagus in lateral view is more rectangular than being narrowly triangular and a slender, membranous distiphallus is often exposed.

#### The *furvitibia* group (*Lamproclasiopa furvitibia*, *Lamproclasiopa xanthocera*)

**Diagnosis.** Body with extensive surfaces sparsely to densely microtomentose. *Head*: Frons and face generally unicolorous; antenna yellow; gena relatively short (gena-to-eye ratio less than 0.06-0.09); genal/postgenal margin rounded. *Thorax*: Presutural supra-alar seta lacking; katepisternum and anepisternum thinly microtomentose, generally appearing dull, not shiny. Wing generally hyaline to very faintly infumate; vein R_2+3_ curved gently apically, not angulate subapically nor bearing a subapical stump vein. Forefemur with 4-5 stout, peg-like setae on apical third along posteroventral margin; tibiae variable, mostly black with apex yellow or entirely yellow. *Abdomen*: Male terminalia: Ventral epandrial margin bearing a cluster of setulae; aedeagus in lateral view with margins irregular.

**Remarks.** This species group is partially based on homoplasious characters, and we cannot confirm its monophyly. The two included species are similar to each other and the species group can be diagnosed. Moreover, the ventral epandrial margins bear a cluster of closely set setulae (also expressed in a few other congeners) and the aedeagus in lateral view is irregular, sinuous. These are the bases for recognition of this species group.

##### Lamproclasiopa furvitibia sp. n.

Taxon classificationAnimaliaDipteraEphydridae

http://www.zoobank.org/9B556FB7-2108-4B56-8999-3D9AC58D4D03

Figs 14 , 82–85

###### Diagnosis.

**Figures 82–85.**
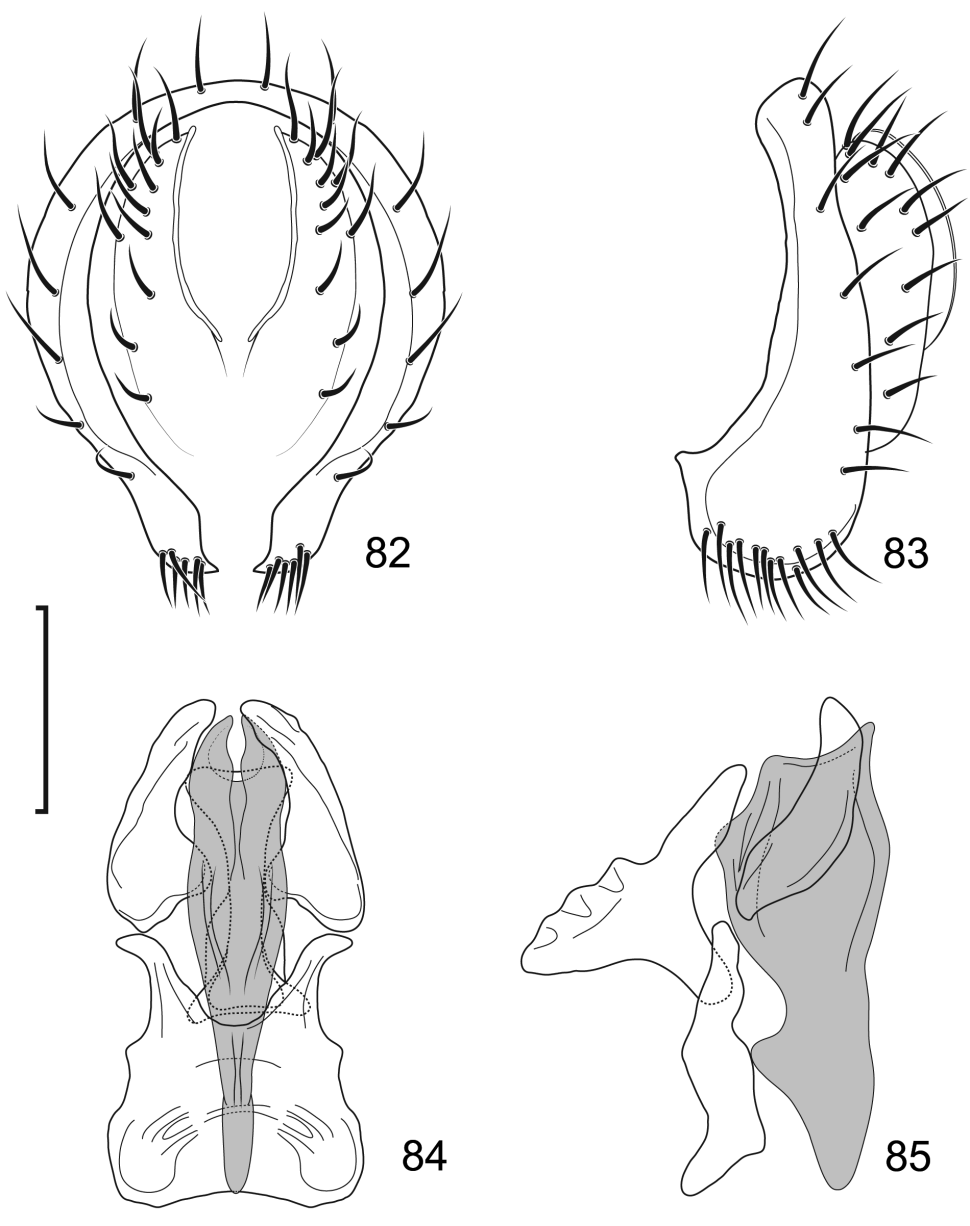
*Lamproclasiopa furvitibia* sp. n. (Costa Rica. San José: Moravia) **82** epandrium and cerci, posterior view **83** same, lateral view **84** internal structures of male terminalia (aedeagus [shaded], phallapodeme, gonite, hypandrium), ventral view **85** same, lateral view. Scale bar = 0.1 mm.

This species can be distinguished from congeners by the following combination of characters: Small to moderately small shore flies, body length 2.00-2.24 mm. *Head*: Frons with yellowish tan to golden tan microtomentum, with slightly silver white microtomentum anteriorly and two small areas shiny black, without microtomentum; mesofrons evident by slight lateral lines. Antenna yellow; basal flagellomere with slightly darker dorsal margin. Face completely and more or less uniformly silvery white microtomentose, lacking vertical stripes; 2 prominent facial setae, dorsal seta at midheight, other seta near epistomal margin; parafacial thin, more densely silvery white microtomentose than face. Gena relatively short, gena-to-eye ratio 0.07–0.09. *Thorax*: Generally black. Mesonotum black with thin, golden brown microtomentum, subshiny, slightly less dense than microtomentum of frons; presutural supra-alar seta lacking; pleural areas more sparsely microtomentose than mesonotum, blackish brown to black, becoming less microtomentose ventrally and posteriorly, subshiny to shiny. Wing completely hyaline, lacking darkened areas; costal vein ratio 0.59–0.60; M vein ratio 0.70–72. Femora grayish to blackish brown, subshiny; forefemur with 4–5 stout, peg-like setae on apical third along posteroventral margin; tibiae blackish brown with distal third yellow; tarsi yellow. *Abdomen*: Tergites shiny black, with little or very sparse microtomentum. Male terminalia (Figs 82–85): Epandrium in posterior view (Fig. 82) nearly as wide as high, as in inverted, bow-shaped U, dorsal arch narrow, becoming wider ventrally, in lateral view (Fig. 83) tear-drop shaped, widest ventrally, ventral margin broadly rounded and bearing distinct row or setulae; cercus in posterior view (Fig. 82) hemispherical, not fused with ventral margin of cercal cavity, ventral portion diffuse, membranous, generally uniformly setulose, in lateral view (Fig. 83) narrowly and irregularly semicircular, slightly wider subdorsally than ventrally; gonite in ventral view (Fig. 84) more or less irregularly triangular, narrowed toward aedeagal base, wider toward hypandrium, in lateral view (Fig. 85) moderately elongate, rod-like, shallowly curved, end toward hypandrium narrower than opposite end; aedeagus in ventral view (Fig. 84) narrowly elongate, 6× longer than wide, basal half wider than narrow apical half, tapered, base narrowly bifurcate, apical half digitiform, apex narrowly rounded; phallapodeme in lateral view (Fig. 85) triangular, angular extensions toward aedeagal base and toward hypandrium digitiform, extension toward aedeagal base longer than extension toward hypandrium, keel robust, tapered, apex narrowly rounded, in ventral view (Fig. 84) elongate, bone-like, with apical and basal cross, both apices truncate; hypandrium in ventral view (Fig. 84) with anterior 2/3 more or less rectangular, lateral margin scalloped, posterior 1/3 slightly flared laterally, thumb-like extensions, posterior margin deeply emarginate, broadly V-shaped, in lateral view (Fig. 85) elongate, shallowly angularly rod-like, an elongate and shallow Z.

###### Type material.

The holotype male of *Lamproclasiopa furvitibia* is labeled “COSTA RICA. Prov. San José. Moravia. Zurquí de Moravia, Tower path. 1600m. 6–13 SEP 2013. Proyeto ZADBI. Malaise trap #1, 0m, ZADBI-1136. -84:00:57 10:02:58 #107741/INB0004403109 INBIOCRI COSTA RICA [plastic bar code label]/HOLOTYPE ♂ *Lamproclasiopa furvitibia* Costa, Mathis & Marinoni MNCR-A [red].” The holotype is double mounted (glued to a paper triangle) and is in very good condition, and is deposited in MNCR-A. Thirty-four paratypes (13♂, 21♀; MNCR-A, USNM) bear the same label data as the holotype. Other paratypes are as follows: COSTA RICA. **Cartago.** Paraíso, Parque Nacional Tapantí (09°43.3'N, 83°46.5'W; 1600 m), 9 Mar – 29 Sep 2013, Proyeto ZADBI (7♂, 14♀; MNCR-A). **Puntaneras.** Coto Brus, Las Alturas Biological Station (08°56.7'N, 82°50'W; 1500–1600 m), 7–13 May 2013, Proyeto ZADBI (1♀; MNCR-A). **Guanacaste.** Macizo Miravalles, Cabro Muco Station (10°43.1'N, 84° 51.3'W; 1100 m), 22 Jun–9 Jul 2003, J. Azoifeifa. (2♂; MNCR-A).

###### Type locality.

Costa Rica. San José. Zurquí de Moravia (10°2.8'N, 84°0.6'W; 1588 m).

###### Distribution

(Fig. 14). Neotropical: Costa Rica (San José, Puntaneras).

###### Etymology.

The species epithet, *furvitibia*, is of Latin derivation, meaning darkened tibia and refers to the partially darkened tibiae, one of the distinguish features of this species.

###### Remarks.

This species is closely related to *Lamproclasiopa xanthocera* but can be distinguished from it by having two small shiny black areas on the anterolateral portion of the frons and by having mostly blackish brown tibiae with the distal third being yellow. The shape of structures of the male terminalia also distinguishes this species from congeners.

##### Lamproclasiopa xanthocera sp. n.

Taxon classificationAnimaliaDipteraEphydridae

http://www.zoobank.org/B35ED0F5-9221-4E9B-A7E5-0E66ED7FADB1

**Figures 86–87.**
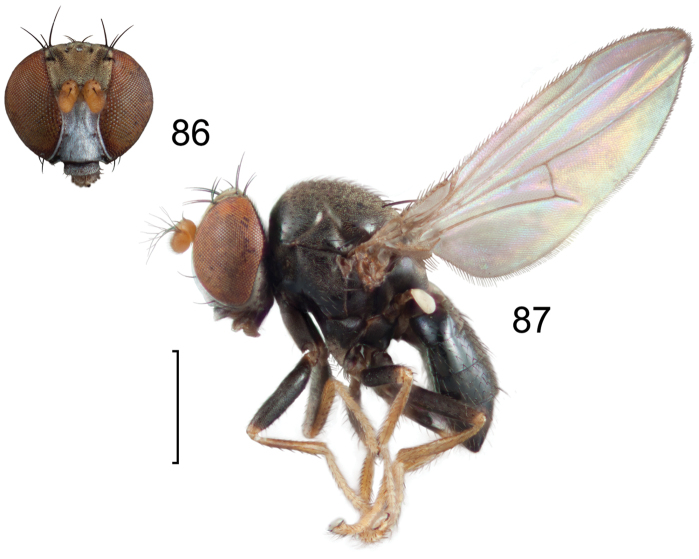
*Lamproclasiopa xanthocera* sp. n., male holotype (Brazil. Paraná: Curitiba) **86** head, anterior view **87** habitus, lateral view. Scale bar = 0.5 mm.

**Figures 88–91.**
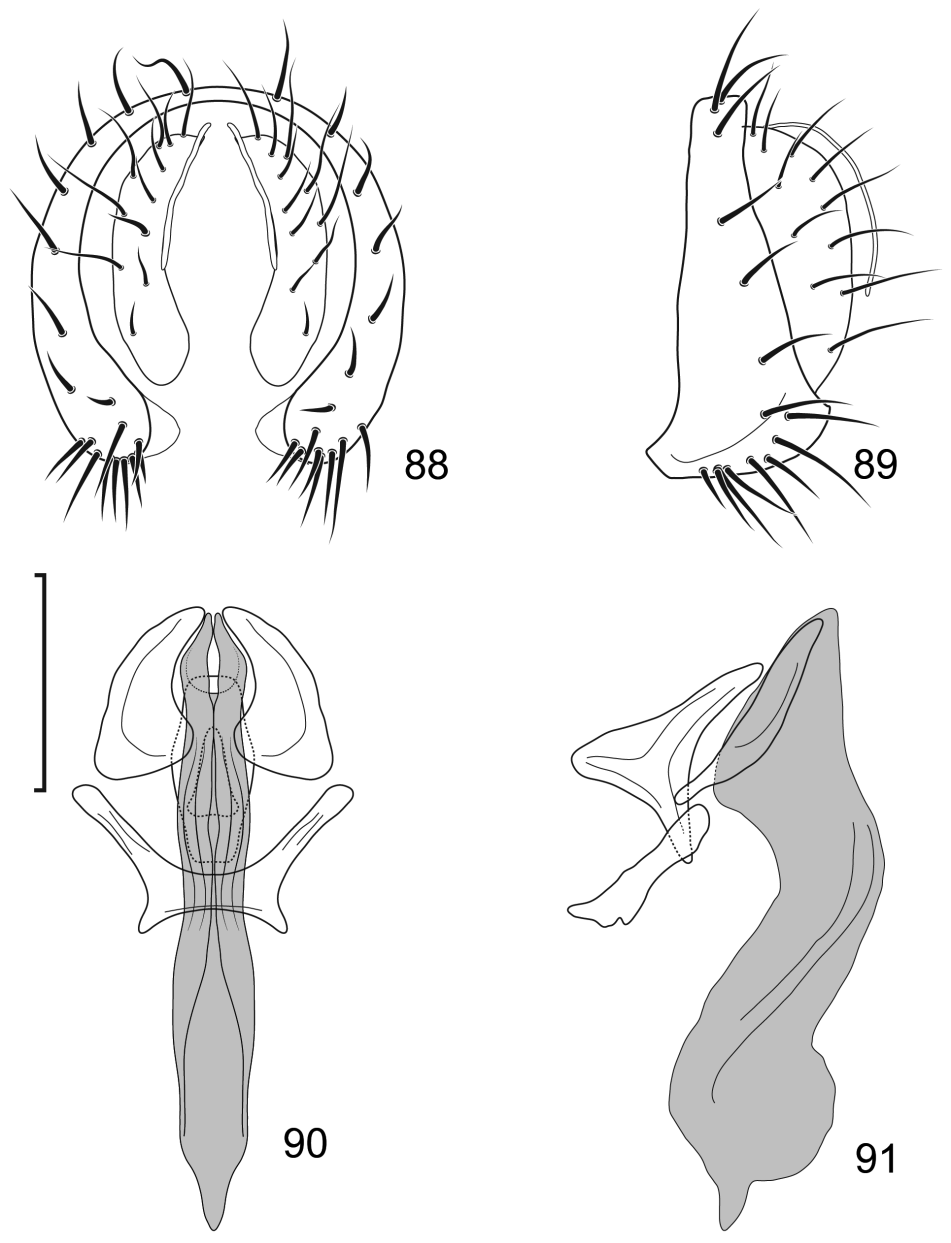
*Lamproclasiopa xanthocera* sp. n., male holotype (Brazil. Paraná: Curitiba) **88** epandrium and cerci, posterior view **89** same, lateral view **90** internal structures of male terminalia (aedeagus [shaded], phallapodeme, gonite, hypandrium), ventral view **91** same, lateral view. Scale bar = 0.1 mm.

Figs 86–87 , 88–91 , 104

###### Diagnosis.

This species can be distinguished from congeners by the following combination of characters: Small to moderately small shore flies, body length 1.73–2.18 mm. *Head*: Frons with yellowish tan to golden tan microtomentum, some areas slightly darker; parafrons with slightly thinner investment of microtomentum; mesofrons evident by slight lateral lines. Antenna yellow; basal flagellomere with slightly darker dorsal margin. Face completely and more or less uniformly silvery white microtomentose, more thinly microtomentose ventrally except for extreme ventral margin, lacking vertical stripes; 2 prominent facial setae, dorsal seta at midheight, other seta near epistomal margin; parafacial thin, more densely silvery white microtomentose than face. Gena relatively short, gena-to-eye ratio 0.06–0.08. *Thorax*: Generally black. Mesonotum black with thin, golden brown microtomentum, subshiny, although less dense than microtomentum of frons; presutural supra-alar seta lacking or indistinguishable from surrounding setae pleural areas more sparsely microtomentose than mesonotum, blackish brown to black, becoming less microtomentose ventrally and posteriorly, subshiny to shiny. Wing completely hyaline, lacking darkened areas; costal vein ratio 0.59–0.60; M vein ratio 0.57–0.65. Femora grayish to blackish brown, subshiny; forefemur with 4–5 stout, peg-like setae on apical third along posteroventral margin; tibiae and tarsi yellow. *Abdomen*: Tergites shiny black, with little or very sparse microtomentum. Male terminalia (Figs 88–91): Epandrium in posterior view (Fig. 88) nearly as wide as long, as in inverted U, dorsal arch narrow, becoming wider ventrally, in lateral view (Fig. 89) narrowly triangular, widest ventrally, ventral margin broadly rounded; cercus in posterior view (Fig. 88) hemispherical, not fused with ventral margin of cercal cavity, uniformly setulose, in lateral view (Fig. 89) narrowly semicircular, slightly wider subdorsally than ventrally; gonite in ventral view (Fig. 90) more or less triangular, narrowed toward aedeagal base, wider toward hypandrium, in lateral view (Fig. 91) elongate, rod-like, end toward hypandrium narrower than opposite end; aedeagus in ventral view (Fig. 90) narrowly elongate, 6× longer than wide, nearly parallel sided, apex pointed, in lateral view (Fig. 91) elongate, L-shaped, short arm basally, wider, thereafter parallel sided, membranous on apical ¼; phallapodeme in lateral view (Fig. 91) triangular, angle toward aedeagal base digitiform, longer than extension toward hypandrium, keel tapered, apex rounded, in ventral view (Fig. 90) rectangular, apical 1/3 to hypandrium slightly tapered, both apices truncate; hypandrium in ventral view (Fig. 90) as a broad, short H with posterior arms flaring posterolaterally, posterior margin broadly emarginate, anterior margin shallowly concave; in lateral view (Fig. 91) elongate, rod-like, anterior apex irregular.

###### Type material.

The holotype male of *Lamproclasiopa xanthocera* is labeled “**BRAZIL.** Paraná: Curitiba, UFPR [Universidade Federal do Paraná, Reserva Biológica] (25°26.9'S, 49°14'W; 915 m),1–2Feb2010[,] D. & W. N. Mathis/USNM ENT 00118308 [plastic bar code label]/HOLOTYPE ♂ *Lamproclasiopa xanthocera* Costa, Mathis & Marinoni DZUP [red].” The holotype is double mounted (minuten pin in a block of plastic), is in excellent condition, and is deposited in DZUP. Paratypes are as follows: BRAZIL. **Paraná.** Morro do Araçatuba (Município de Tijucas do Sul; 25°53.8'S, 49°01.2'W; 910 m), 27 Feb 2015, W. N. Mathis (2♀; DZUP, USNM). **São Paulo.** Estação Biológica de Boracéia, Salesópolis (23°32'S, 45°50.8'W), Aug 1969, N. Papavero (1♀; MZUSP).

###### Type locality.

Brazil. Paraná. Curitiba, Universidade Federal do Paraná, Reserva Biológica (25°26.9'S, 49°14'W; 915 m).

###### Distribution

(Fig. 104). Neotropical: Argentina, Brazil (Paraná, São Paulo).

###### Etymology.

The species epithet, *xanthocera*, is of Latin derivation, meaning yellow horn and refers to the yellow antenna, one of the distinguishing features of this species.

###### Remarks.

Although similar to *Lamproclasiopa bisetulosa*, this species is distinguished from it and other congeners by having a generally microtomentose body, yellow antenna with little or no darkening along dorsal surfaces, a hyaline wing, and a blackish yellow foretarsus. The shape of structures of the male terminalia also distinguishes this species, especially the elongate, thick, and conspicuously sinuous aedeagus with an apical papilla-like apex.

#### The *nadineae* group (*Lamproclasiopa aliceae*, *Lamproclasiopa argentipicta*, *Lamproclasiopa nadineae*)

**Diagnosis.** Body generally shiny black. *Head*: Frons and face generally unicolorous; gena moderately high (gena-to-eye ratio 0.13–0.22); genal/postgenal margin rounded. *Thorax*: Presutural supra-alar seta lacking; katepisternum, especially anterior half, and anteroventral portion of anepisternum shiny black. Wing generally hyaline to faintly infumate; vein R_2+3_ curved gently apically, not angulate subapically nor bearing a subapical stump vein. Forefemur with 4–5 stout, peg-like setae on apical third along posteroventral margin.

**Remarks.** Two of the species in this species group, *Lamproclasiopa aliceae* and *Lamproclasiopa nadineae*, form a monophyletic lineage that is characterized by synapomorphies (presutural supra-alar seta lacking; katepisternum, especially anterior half, and anteroventral portion of anepisternum shiny black; forefemur with 4–5 stout, peg-like setae on apical third along posteroventral margin). These two species are likewise unique in occurring only in the Nearctic Region. The inclusion of *Lamproclasiopa argentipicta* in this group may be artificial, having a homoplasious basis. We have not discovered a synapomorphy that is unique to these three species.

##### Lamproclasiopa aliceae sp. n.

Taxon classificationAnimaliaDipteraEphydridae

http://www.zoobank.org/FE0C666F-5C59-416E-A3DE-E66AB7937A60

**Figures 92–93.**
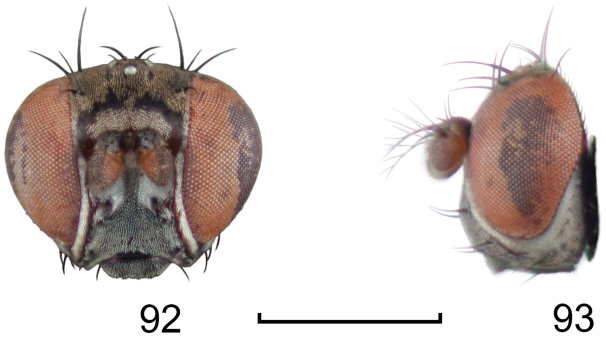
*Lamproclasiopa aliceae* sp. n. (USA. New Mexico: Silver City) **92** head, anterior view **93** same, lateral view. Scale bar = 0.5 mm.

**Figures 94–97.**
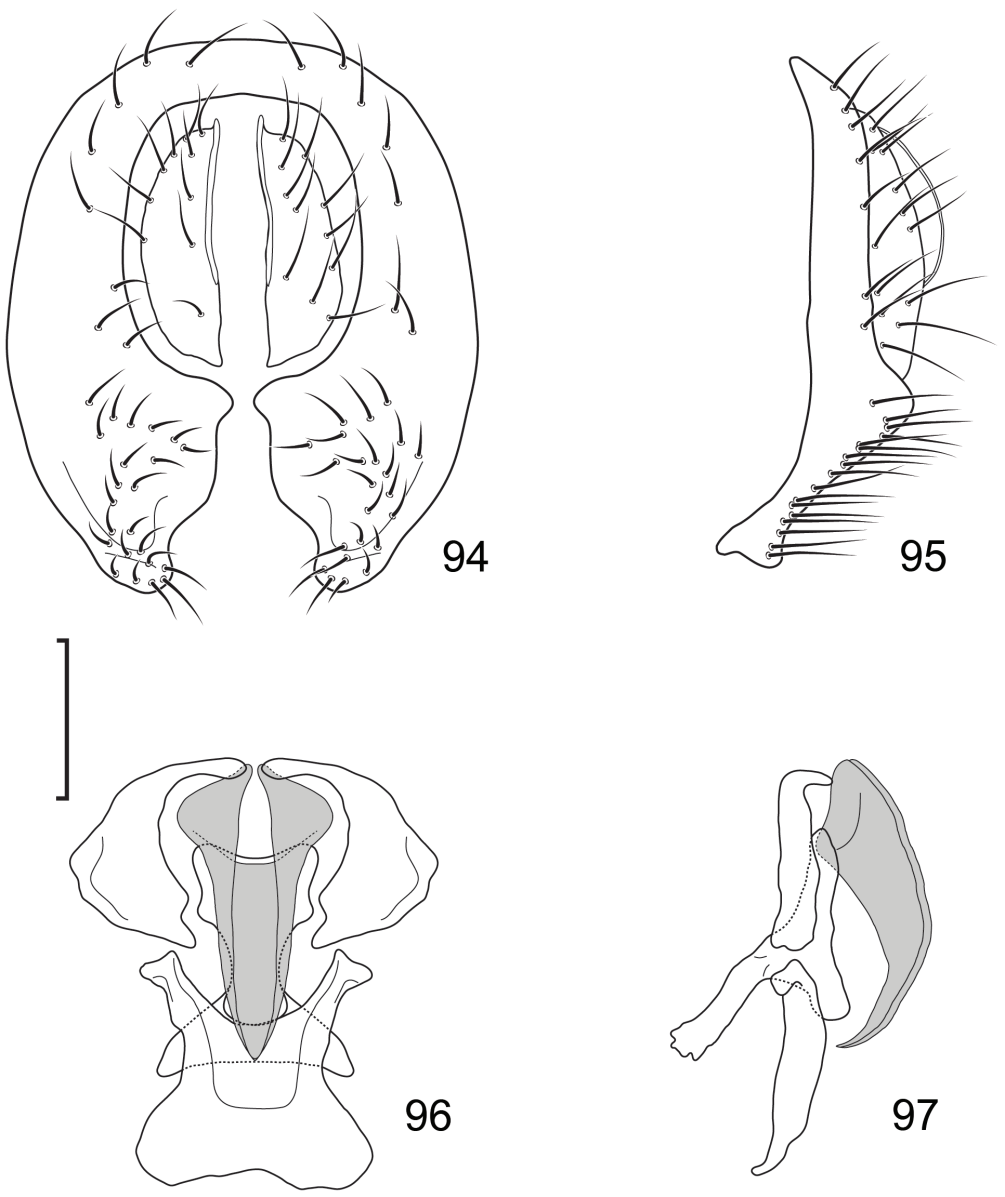
*Lamproclasiopa aliceae* sp. n. (USA. New Mexico: Silver City) **94** epandrium and cerci, posterior view **95** same, lateral view **96** internal structures of male terminalia (aedeagus [shaded], phallapodeme, gonite, hypandrium), ventral view **97** same, lateral view. Scale bar = 0.1 mm.

Figs 92–93 , 94–97 , 111

###### Diagnosis.

This species is easily distinguished from congeners by the following combination of characters: Small to moderately small shore-flies, body length 1.80–2.10 mm. *Head*: Frons with dorsal 2/3 bearing brown, moderately sparse microtomentum, thereafter ventrally with a transverse bear, shiny black band, then a more grayish microtomentose, transverse band just before margin and dorsad of antennal bases. Ventral portion of face mostly unicolorous, moderately grayish brown microtomentose, antennal grooves more densely, whitish gray microtomentose; parafacial grayish white to creamy white. Gena moderately high, height subequal to height of basal flagellomere. Gena-to-eye ratio 0.14–0.17. *Thorax*: Mesonotum moderately sparsely brown microtomentose, mostly appearing subshiny black, lacking elongate spots; presutural supra-alar seta lacking. Katepisternum, especially anterior half, and anteroventral portion of anepisternum shiny black. Wing hyaline and lacking stump veins; costal vein ratio 0.55–0.63; M vein ratio 0.51–0.55. Forefemur with 4–5 stout, peg-like setae on apical third along posteroventral margin; femora black; tibiae with basal 2/3-¾ black, apical ¼-1/3 yellowish; tarsi yellow. *Abdomen*: Tergites very sparsely microtomentose medially to complete bare laterally, mostly shiny black. Male terminalia (Figs 94–97): Epandrium in posterior view (Fig. 94) roundly U-shaped except for incised ventromedial opening or pocket, widest at midheight, each lateral arm distinctly wider basally, ventromedial pocket between epandrial arms bottle-shaped, with dorsal half as a narrowed neck and ventral half wider than high, in lateral view (Fig. 95) narrow, angulate, widest just ventrad of cercal cavity, angulate, epandrial arm expanded at apex; cercus hemispherical, gradually tapered ventrally to broadly rounded apex, evenly setulose over length; gonite in lateral view (Fig. 97) robustly rod-like, essentially straight, narrower and abruptly curved toward aedeagal base, in ventral view (Fig. 96) clavate, wider toward hypandrium, much narrower, digitiform on extension toward aedeagal base; aedeagus in lateral view (Fig. 97) more or less funnel-like, curved subapically, widest basally, thereafter apically curved to acute point, in ventral view (Fig. 96) longer than wide, straight, base expanded, lateral margins of base rounded, thereafter apically evenly tapered to point; phallapodeme in lateral view (Fig. 97) as an inverted Y with an elongate, narrow, extension toward aedeagal base, keel elongate, narrow, parallel sided, apex irregularly rounded, in ventral view (Fig. 96) hourglass-like, sub-rectangular at apex toward aedeagal base, broadly expanded with lateral phalanges toward hypandrium, this apex truncate; hypandrium in lateral view (Fig. 97) elongate, narrow, bar-like, narrowed anteriorly, in ventral view (Fig. 96) robustly X-shaped to quadrate with lateral and poster margins concave.

###### Type material.

The holotype male of *Lamproclasiopa aliceae* is labeled “**U[nited]S[tates of]A[merica]. N[ew]M[exico].** Grant: Silver City (32°46.4'N, 108°16.5'W; 1790 m), 14 Aug 2007,D.&W.N.Mathis/USNM ENT 00118306 [plastic bar code label]/HOLOTYPE ♂ *Lamproclasiopa aliceae* Costa, Mathis & Marinoni USNM [red].” The holotype is double mounted (minuten pin in a block of plastic), is in excellent condition, and is deposited in the USNM. Eight paratypes (7♂, 1♀; DZUP, USNM) bare the same label data as the holotype.

###### Type locality.

United States. New Mexico. Grant: Silver City (Big Ditch; 32°46.4'N, 108°16.5'W; 1790 m). The “Big Ditch” is a large, canal-sized ditch that traverses Silver City, and during dry-weather seasons, the ditch has a small stream running through it. The ditch is frequently scoured out when heavy rains occur, sometimes resulting in flash floods in the “Big Ditch.” The type series was collected when dry weather prevailed.

###### Distribution

(Fig. 111). Nearctic: United States (New Mexico).

###### Etymology.

The species epithet, *aliceae*, is a Latin genitive patronym to honor Alice Joy Brown† (nee Peacock, 1931–2016), a wonderful friend and supporter.

###### Remarks.

Although similar and apparently closely related to *Lamproclasiopa nadineae*, this species is distinguished from that species by the shape of the ventral portion of the epandrium, which has a bottle-shaped gap ventrally between the lateral arms. From other congeners, this species is distinguished by the shiny black katepisternum, especially its anterior half, and the anteroventral portion of the anepisternum. The forefemur also bears 4–5 stout, peg-like setae on the apical half of the posteroventral margin.

##### Lamproclasiopa argentipicta sp. n.

Taxon classificationAnimaliaDipteraEphydridae

http://www.zoobank.org/D26C06A0-302A-4EDE-B2E5-70D6C9DB50E7

**Figures 98–99.**
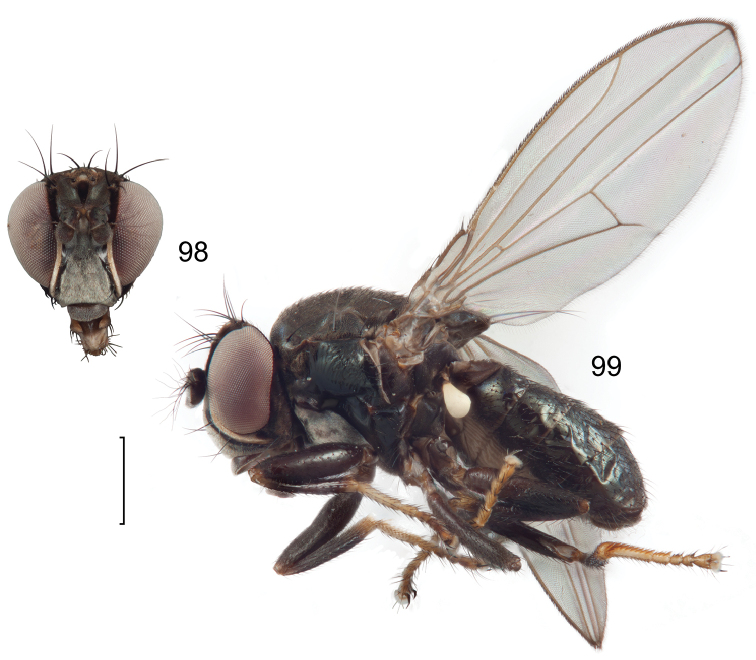
*Lamproclasiopa argentipicta* sp. n., male paratype (Costa Rica. San José: Moravia) **98** head, anterior view. **99** habitus, lateral view. Scale bar = 0.5 mm.

**Figures 100–103.**
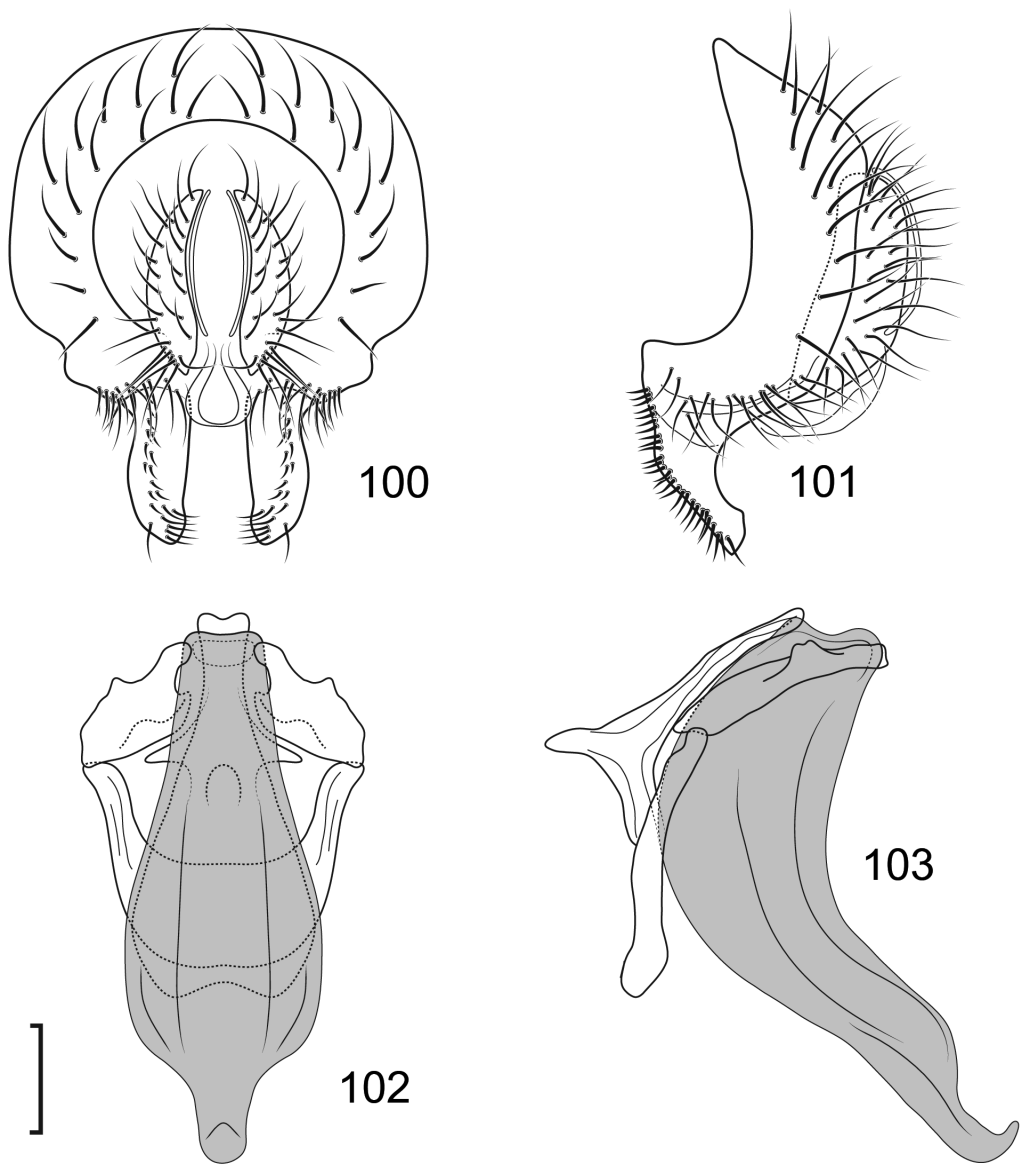
*Lamproclasiopa argentipicta* sp. n., male paratype (Costa Rica. San José: Moravia) **100** epandrium and cerci, posterior view **101** same, lateral view **102** internal structures of male terminalia (aedeagus [shaded], phallapodeme, gonite, hypandrium), ventral view **103** same, lateral view. Scale bar = 0.1 mm.

**Figure 104.**
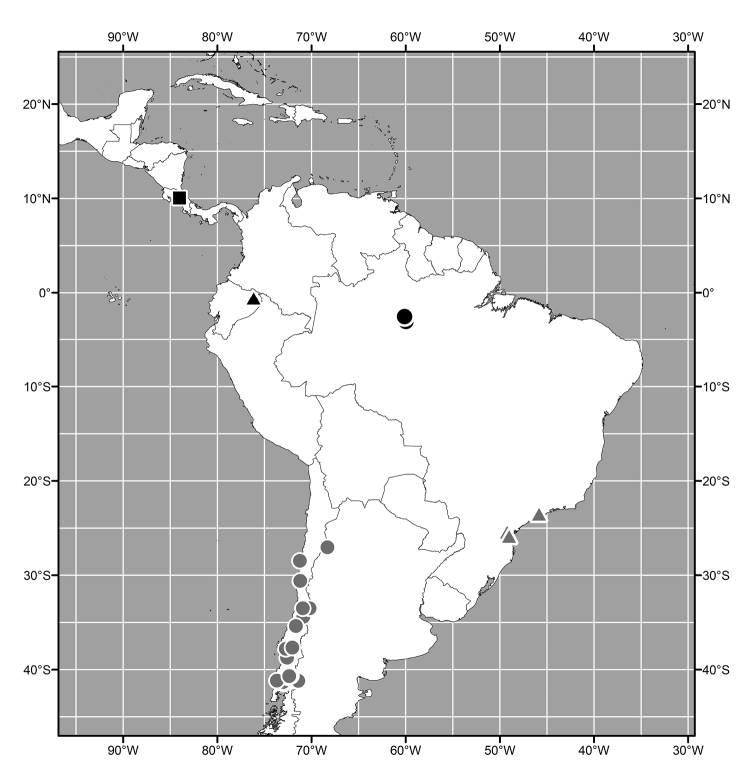
Distribution map of *Lamproclasiopa argentipicta* sp. n. (■); *Lamproclasiopa zerafael* sp. n. (●); *Lamproclasiopa ecuadoriensis* sp. n. (▲); *Lamproclasiopa polita* (●); *Lamproclasiopa xanthocera* sp. n. (▲).

Figs 98–99 , 100–103 , 104

###### Diagnosis.

This species is distinguished from congeners by the following combination of characters: Moderately small to medium-sized shore flies, body length 2.90–3.20 mm. *Head*: Frons with two longitudinal, grayish microtomentose stripes; fronto-orbits and narrow, medial triangular area shiny black. Antenna blackish brown. Face with light silver microtomentum, except for shiny black lateral margins; parafacials white, microtomentose. Gena moderately high, gena-to-eye ratio 0.16–0.22. *Thorax*: Mesonotum shiny black, covered with brownish microtomentum; presutural supra-alar seta lacking or indistinguishable from surrounding setae; pleural region less microtomentose, anepisternum and katepisternum almost bare, concolorous with mesofrons. Wing hyaline, lacking any pattern or markings. Costal vein ratio 0.45–0.50; M vein ratio 0.57. Forecoxae light gray, mid and hind coxae blackish brown; forefemur with 4–5 stout, peg-like setae on apical third along posteroventral margin; femora and tibiae blackish brown, except for distalmost part of tibiae, yellowish; tarsi yellow. *Abdomen*: Generally shiny blackish brown, sparsely microtomentose; tergites 5 larger than previous tergites. Male terminalia (Figs 100–103): Epandrium in posterior view (Fig. 100) with dorsal 2/3 quadrate, as wide as high, corners rounded, ventral third as 2 thumb-like projections, dorsal portion thickly developed, as wide or wider than width of lateral structure, setulae evenly distributed dorsally and laterally, becoming very sparse ventrally, ventral extensions bearing tiny setulae in verticomedial alignment, apically with cluster of small setulae, in lateral view (Fig. 101) as 2 right angles, dorsal portion more robust, thick, then a right anterior angle, then a ventral right angle to form digitiform extension that bears closely set setulae along anterior margin; cerci in posterior view (Fig. 102) elongate, moderately thin, generally shallowly arched, slightly ventrally than dorsally, dorsal angle with vertex narrowly rounded, ventral apex acutely pointed, in lateral view elongate, narrow, ventral portion tapered; aedeagus in lateral view (Fig. 103) as an irregular funnel, tapered from thick base to pointed apex, narrowed more abruptly on apical 1/8 then narrowly pointed at right angle, in ventral view (Fig. 102) generally clavate, gradually becoming wider from truncate base toward apex, widest subapically, thereafter abruptly narrowed to slender, digitiform apex; phallapodeme in lateral view (Fig. 103) narrowly triangular with vertex toward aedeagal base elongate and narrow, keel narrow, subequal to process extended toward hypandrium, in ventral view (Fig. 102) narrow, elongate with a sub-basal cross-piece and narrow, lateral extensions, thereafter almost parallel sided; gonite in lateral view irregularly rod-like, in ventral view (Fig. 102) wider than high and with a mediobasal, short, digitiform projection; hypandrium in lateral view (Fig. 103) thin, elongate, rod-like, shallowly curved, slightly wider anteriorly than posteriorly, in ventral view (Fig. 102) robustly U-shaped with thickened base, broadly rounded anterior margin and deeply U-shaped posterior emargination.

###### Type material.

The holotype male of *Lamproclasiopa argentipicta* is labeled “COSTA RICA. Prov. San José. Moravia. Zurquí de Moravia, Tower path. 1600m. 9–16 AGO 2013. Proyeto ZADBI. Malaise trap #1, 0m, ZADBI-1018. -84:00:57 10:02:58 #107537/INB0004433874 INBIOCRI COSTA RICA [plastic bar code label]/HOLOTYPE ♂ *Lamproclasiopa argentipicta* Costa, Mathis & Marinoni MNCR-A [red].” The holotype is double mounted (glued to a paper triangle) and is in very good condition, and is deposited in MNCR-A. Three paratypes (1♂, 2♀; MNCR-A, USNM) bear the same label data as the holotype.

###### Type locality.

Costa Rica. San José. Zurquí de Moravia (10°2.8'N, 84°0.6'W; 1588 m).

###### Distribution

(Fig. 104), Neotropical: Costa Rica (San José).

###### Etymology.

The species epithet, *argentipicta*, is of Latin derivation and means painted with silver, referring to the silver microtomentose areas of this species, especially its face.

###### Remarks.

This species is apparently closely related to *Lamproclasiopa hendeli*, based on external features, such as the shiny black body and the face that is covered with silver gray microtomentum. The male terminalia, however, are unique within *Lamproclasiopa*, with two setulose projections ventrally and with the ventral projection of the phallapodeme being very thin, appearing to be almost fused with the hypandrium.

##### Lamproclasiopa nadineae

Taxon classificationAnimaliaDipteraEphydridae

(Cresson)

**Figures 105–106.**
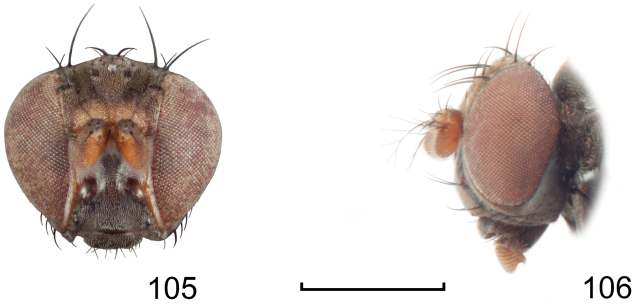
*Lamproclasiopa nadineae* (Cresson). (USA. California. Jasper Ridge Biological Preserve) **105** head, anterior view **106** same, lateral view. Scale bar = 0.5 mm.

**Figures 107–110.**
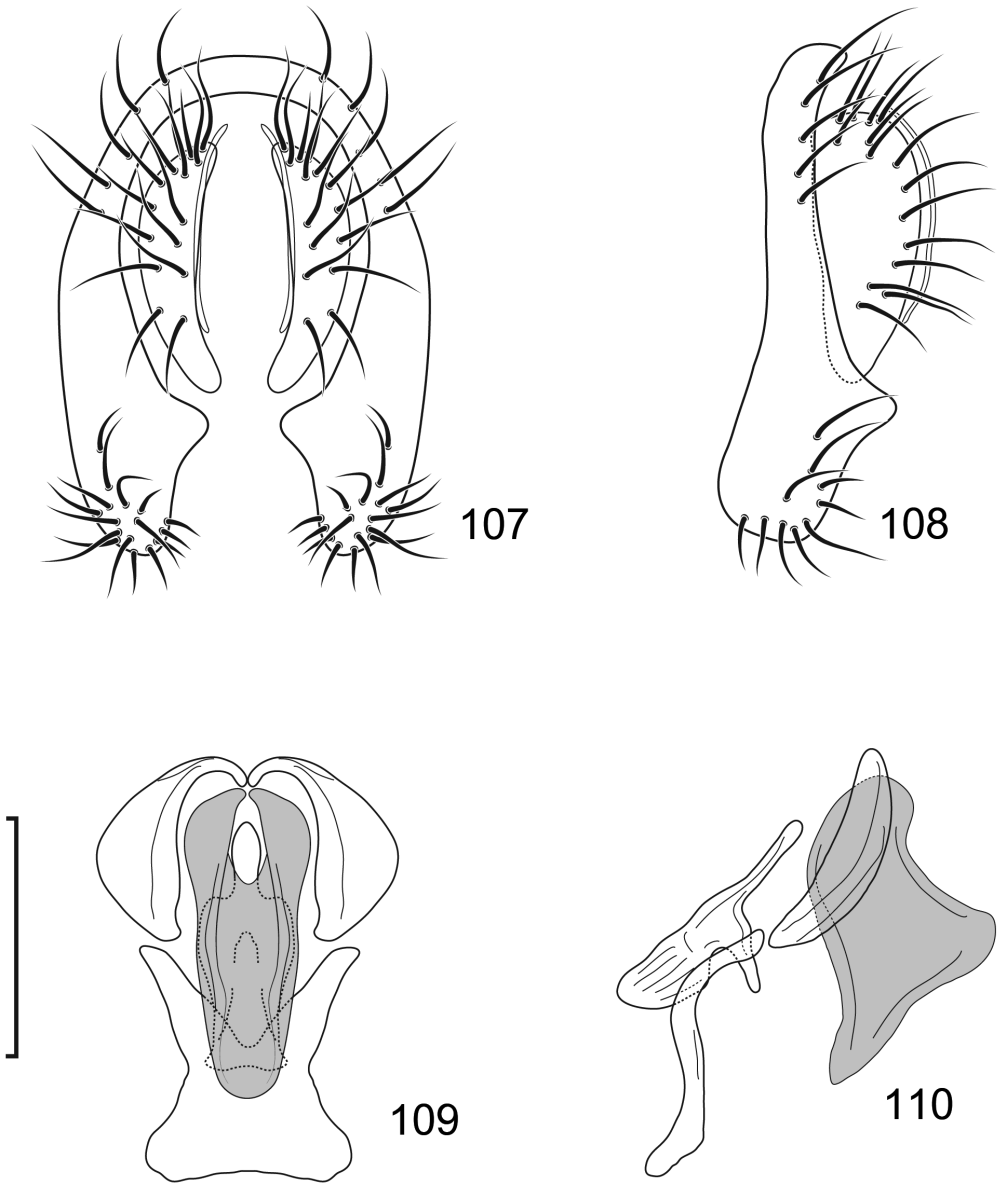
*Lamproclasiopa nadineae* (Cresson). (USA. California. Jasper Ridge Biological Preserve) **107** epandrium and cerci, posterior view **108** same, lateral view **109** internal structures of male terminalia (aedeagus [shaded], phallapodeme, gonite, hypandrium), ventral view **110** same, lateral view. Scale bar = 0.1 mm.

**Figure 111.**
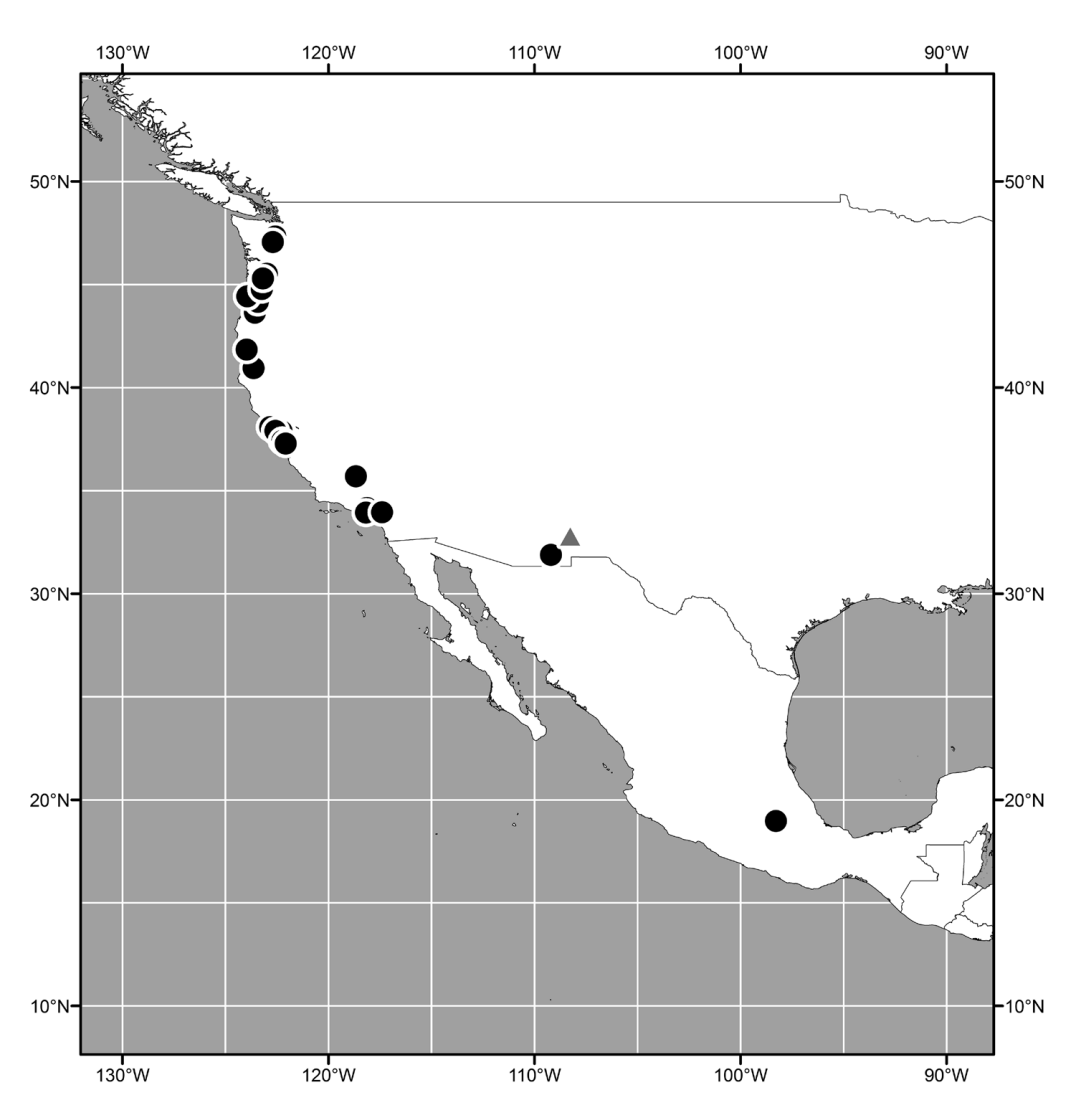
Distribution map of *Lamproclasiopa aliceae* sp. n. (▲); *Lamproclasiopa nadineae* (●).

Figs 105–106 , 107–110 , 111

Ditrichophora nadineae Cresson 1925b: 166.
Discocerina (Basila) nadineae . Cresson 1942: 116 [generic combination]. Wirth 1965: 738 [Nearctic catalog]. Mathis and Zatwarnicki 1995: 165 [world catalog].
Lamproclasiopa nadineae . Zatwarnicki and Mathis 2001: 39 [generic combination].

###### Diagnosis.

This species is distinguished from congeners by the following combination of characters: Small to moderately small shore-flies, body length 1.65–2.50 mm. *Head*: Frons generally microtomentose but unevenly, microtomentum on ocellar triangle and especially along anterior margin just dorsad of antennal bases gray and denser, otherwise sparse and grayish brown to brown, areas toward anterior margin of frons yellowish orange to red; ocellar triangle extended to anterior margin. Antenna dark brown dorsally, extensively yellow to yellowish orange ventrobasally. Face generally microtomentose, becoming bare laterally, most prominent anteriorly in lateral view at ventral margin of antennal grooves; parafacial bare of ventroclinate setulae. Gena moderately high, gena-to-eye ratio 0.13–0.17. *Thorax*: Mesonotum black with uniform, fine, thin investment of gray to brown microtomentum, lacking pattern of spots; presutural supra-alar seta lacking or indistinguishable from surrounding setae. Katepisternum, especially anterior half, and anteroventral portion of anepisternum shiny black. Wing hyaline, lacking any maculation pattern or stump veins; costal vein ratio 0.50–0.52; M vein ratio 0.55–0.61. Forefemur with 4–5 stout, peg-like setae on apical third along posteroventral margin; legs generally black except for yellowish apices, yellowish apices of tibiae more extensive; tarsi yellow. *Abdomen*: Tergites subshiny to shiny black, generally lacking microtomentum or very sparse. Male terminalia (Figs 107–110): Epandrium in posterior view (Fig. 107) roundly U-shaped, except for ventral opening, oval, narrower dorsally, broadly rounded, widest at midheight, each lateral arm distinctly wider, ventromedial margin nearly straight, bearing cluster of setae ventrally; cercus hemispherical, gradually tapered ventrally to broadly rounded apex, evenly setulose over length; gonite in lateral view (Fig. 108) robustly rod-like, slightly curved, narrower toward hypandrium, in ventral view clavate, wider toward hypandrium, much narrower, digitiform on extension toward aedeagal base; aedeagus in lateral view (Fig. 110) more or less rectangular, widest apically and anteroventral corner more narrowly produced than posterodorsal corner, base rounded with slight, dorsal extension, in ventral view (Fig. 109) as a relatively short, moderately narrow structure, base rounded and with an medioapical papilla, apex moderately broadly rounded; phallapodeme in lateral view (Fig. 110) as a spike-heeled shoe with an elongate, narrow, extension toward aedeagal base, keel moderately long, narrow, irregularly rounded, in ventral view (Fig. 109) with thumb-like narrow process toward aedeagal base, thereafter toward hypandrium with right angle laterally, hypandrial 2/3 widest sub-basally thereafter concave to truncate apex; hypandrium in lateral view (Fig. 110) conspicuously sinuous, narrow, elongate, in ventral view robustly X-shaped with truncate anterior margin, lateral margins concave, posterior margin deeply V-shaped.

###### Type material.

The holotype male of *Ditrichophora nadineae* Cresson is labeled “Berkeley Hills Alameda Co. IV.20.’08. Cal./♂/TYPE Ditrichophora NADINEAE E. T. Cresson. Jr. [maroon red; “Ditrichophora NAEINEAE” handwritten.” The holotype is double mounted (minuten pin in a thin, rectangular piece of card), is in excellent condition, and is deposited in the ANSP (6348). Four paratypes (1♂, 3♀; ANSP) bear the same locality label as the holotype.

###### Type locality.

United States. California. Alameda: Berkeley Hills (37°53.5N, 122°16.1'W).

###### Other specimens examined.

UNITED STATES. Arizona. **Cochise**: Portal, Southwestern Research Station (31°53'N, 109°12.4'W), 5–9 Jun 1972, W. W. Wirth (1♂; USNM). California. **Alameda**: Berkeley (37°52.3'N, 122°16.4'W), 23 Mar 1919, B. Brookman, T. Aitken (1♀; USNM). Berkeley Hills (37°53.5'N, 122°16.1'W), 8 Mar-11 May 1908 (2♂, 3♀; ANSP). **Humboldt**: Willow Creek (40°56.4'N, 123°37.9'W), 26 Jul 1951, A. H. Sturtevant (1♂; USNM). **Kern**: Kern Canyon (35°41.8'N, 118°40.1'W), 1 Apr 1950, A. H. Sturtevant (2♂, 1♀; USNM). **Los Angeles**: Arcadia, Arboretum (34°8.6'N, 118°3.2'W), 14 Set 1949, A. H. Sturtevant (2♂, 5♀; USNM); Pasadena (34°9.4'N, 118°7.9'W), 15, 17 Feb 1950, A. H. Sturtevant (3♂, 3♀; USNM); Rio Hondo (33°55.9'N, 118°10.5'W), 15 Feb 1950, A. H. Sturtevant (2♂, 2♀; USNM). **Marin**: Lagunitas Creek (38°4.8'N, 122°49.6'W), 19 Oct 1947, W. W. Wirth (1♂; USNM); Muir Woods (37°53.6'N, 122°34.4'W), 7 Aug 1915, A. L. Melander (1♀; ANSP, USNM). **Riverside**: Riverside (33°57.2'N, 117°23.8'W), 3 Feb-5 May 1935, A. L. Melander (2♂, 1♀; ANSP, 5♂, 3♀; USNM). **San Mateo**: Corte Madera Creek (37°24.1'N, 122°14.3'W), 28 Oct 1953, P. H. Arnaud (1♂, 1♀; USNM); Stanford University, Jasper Ridge Biological Preserve (37°24'N, 122°14.5'W; 110 m), 14-17 Feb 2006, 2007, P. H. Arnaud, Jr. & M. M. Arnaud (29♂, 20♀; USNM); Searsville Lake (37°24.3'N, 122°14.3'W), 5 May 1953, P. H. Arnaud (1♂, 3♀; USNM). **Santa Clara**: Stevens Creek County Park (37°17.1'N, 122°4.6'W), 13 Mar 1976, L. Bezark (1♂; USNM). **Siskiyou**: Gasquet (41°50.7'N, 123°58.2'W), 18 Sep 1934, A. L. Melander (1♀; ANSP). Oregon. **Benton**: Cary’s Grove (44°22.6'N, 123°36.1'W), 2 Sep 1974, W. N. Mathis (2♂, 3♀; USNM); Corvallis (44°33.9'N, 123°15.7'W), 1 Aug 1935, K. Gray, (1♂; USNM); Philomath (1.6 km SW; 44°31.8'N, 123°22.9'W), 29 May 1972, W. N. Mathis (1♀; USNM); Rock Creek (6.4 km SW Philomath; 44°30.1'N, 123°26.2'W), 29 May 1972, W. N. Mathis (2♀; USNM). **Douglas**: Elkton (43°38.25'N, 123°34.1'W), 28 Jul 1951, A. H. Sturtevant (1♀; USNM). **Lane**: Burp Hollow at Long Tom River (44°09.5'N, 123°25.4'W), 30 Jun 1988, R. Danielsson (3♂; MZLU). **Lincoln**: Waldport (10.4 km E; 44°25.6'N, 123°56.25'W), 27 May 1972, W. N. Mathis (4♂, 5♀; USNM). **Polk**: Helmick State Park (44°46.9'N, 123°14.2'W), 20 March 1972, W. N. Mathis (1♂; USNM). **Washington**: Hillsboro (45°31.3'N, 122°59.3'W), 9 Apr 1936, K. Gray, J. Shuh (1♀; USNM). **Yamhill**: Carlton (45°17.6'N, 123°10.65'W), 31 Jul 1951, A. H. Sturtevant (1♂, 2♀; USNM).

Washington. **Pierce**: Dupont (5 km WSW; 47°03.8'N, 122°41.7'W), 13 Apr 1971, W. N. Mathis (3♀; USNM); Gig Harbor (47°19.8'N, 122°34.8'W), 17 May-9 Jun 1971, W. N. Mathis (18♂, 3♀; USNM).

MEXICO. **Puebla**: Puebla (9.6 km SW; 18°58.4'N, 98°16.9'W), 2 Jul 1953, University of Kansas Mexican Expedition (1♂; USNM).

###### Distribution

(Fig. 111). Nearctic: United States (California, New Mexico, Oregon, Washington). Neotropical: Mexico (Puebla).

###### Remarks.

Although similar and apparently closely related to *Lamproclasiopa aliceae*, this species is distinguished from that species and other congeners by the absence of a presutural supra-alar seta; the shiny black katepisternum, especially the anterior half; the shiny black anteroventral portion of the anepisternum; and the presence of four to five stout, peg-like setae on the apical third of the forefemur along the posteroventral margin. The shape of structures of the male terminalia also distinguish this species from *Lamproclasiopa aliceae*. So far as we know, however, the distribution of this species and of *Lamproclasiopa aliceae* do not overlap, with this species only known from the west coast of North America and Puebla in Mexico. The non-overlapping distributions of these two species may be a function of sample error, however.

#### The *puella* group (*Lamproclasiopa aracataca*, *Lamproclasiopa bisetulosa*, *Lamproclasiopa caligosa*, *Lamproclasiopa curva*, *Lamproclasiopa fumipennis*, *Lamproclasiopa puella*)

**Diagnosis.** Head, thorax, and abdomen variable, either generally shiny black or with extensive surfaces sparsely to densely microtomentose. *Head*: Frons and face generally unicolorous; gena moderately high (gena-to-eye ratio less than 0.11–0.19); genal/postgenal margin rounded. *Thorax*: Presutural supra-alar seta well developed; katepisternum either thinly microtomentose, generally appearing dull, not shiny, or especially anterior half, and anteroventral portion of anepisternum shiny black. Wing generally hyaline to very faintly infumate; vein R_2+3_ curved gently apically, not angulate subapically nor bearing a subapical stump vein. Forefemur with posteroventral setae slender, not stout and peg-like.

**Remarks.** This is the largest species group with six included species and its recognition is based on homoplasious characters. Thus, the group may be artificial. The included species are quite similar, however, and are the bases for our recognition and diagnosis of the group.

##### Lamproclasiopa aracataca

Taxon classificationAnimaliaDipteraEphydridae

(Cresson)

**Figures 112–113.**
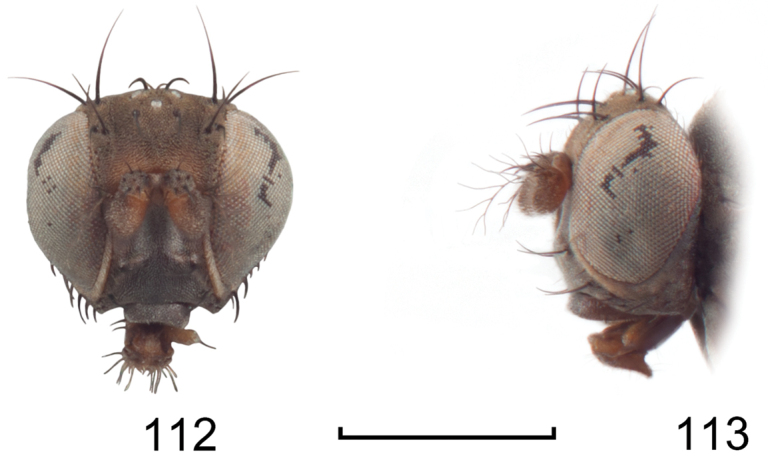
*Lamproclasiopa aracataca* (Cresson). (Chile. Bío Bío: Santa Barbara) **112** head, anterior view **113** same, lateral view. Scale bar = 0.5 mm.

**Figures 114–117.**
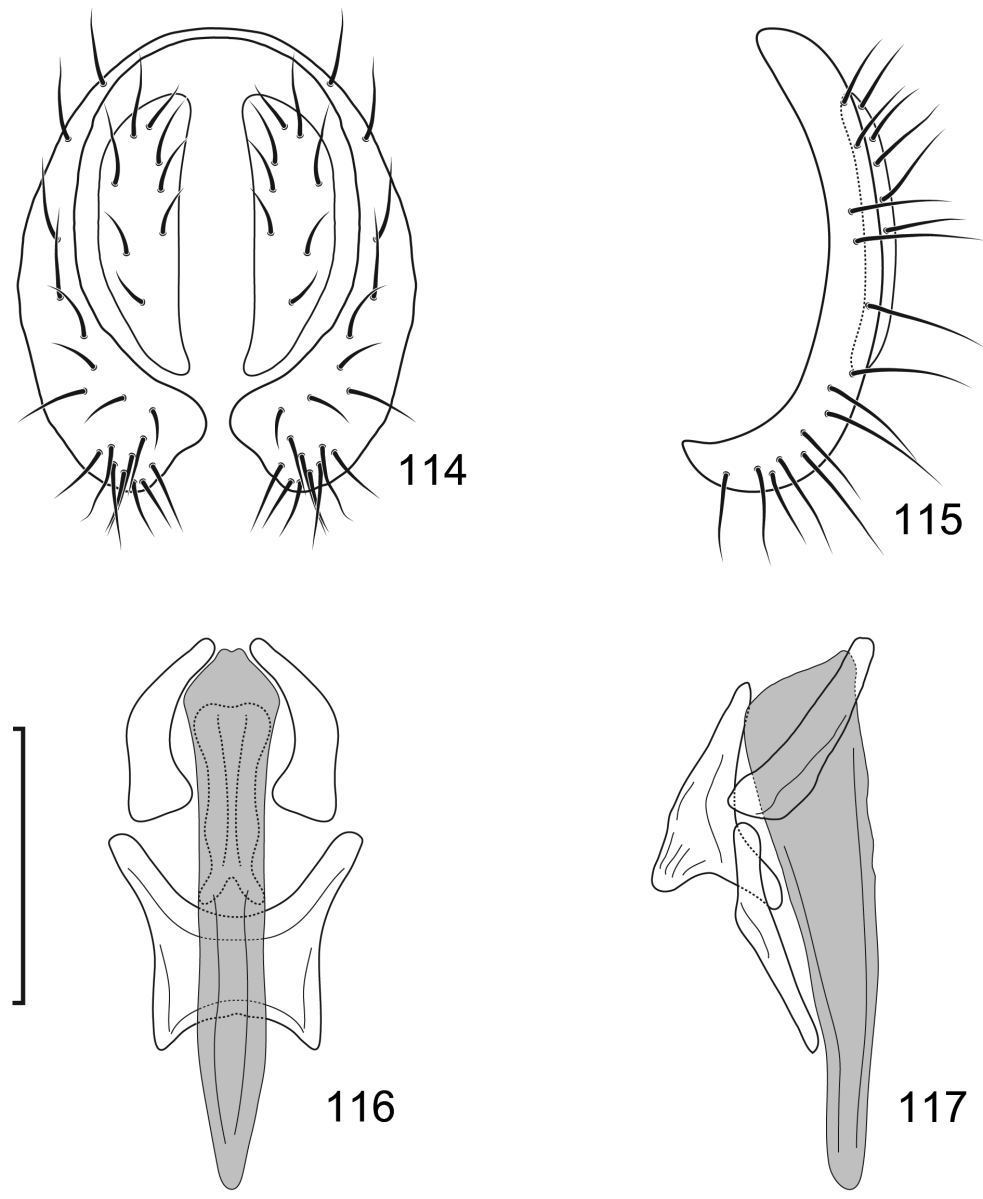
*Lamproclasiopa aracataca* (Cresson). (Chile. Bío Bío: Santa Barbara) **114** epandrium and cerci, posterior view **115** same, lateral view **116** internal structures of male terminalia (aedeagus [shaded], phallapodeme, gonite, hypandrium), ventral view **117** same, lateral view. Scale bar = 0.1 mm.

Figs 81 , 112–113 , 114–117

Discocerina aracataca Cresson 1940: 5.
Discocerina (Lamproclasiopa) aracataca . Cresson 1946: 149 [review]. Wirth 1968: 7 [Neotropical catalog]. Mathis and Zatwarnicki 1995: 168 [world catalog].
Lamproclasiopa aracataca . Zatwarnicki and Mathis 2001: 36 [generic combination].

###### Diagnosis.

This species is distinguished from congeners by the following combination of characters: Small to moderately small shore flies, body length 1.80-2.35 mm. *Head*: Frons dull, anterior margin yellowish orange in some degree, posterior portion grayish black, concolorous with mesonotum, some specimens with frons entirely grayish black, without distinctly marked iridescent microtomentose stripes. Antenna mostly grayish black to black, only ventral margin of segments yellowish orange. Face nearly unicolorous, blackish gray, not distinctively marked; parafacial bare of ventroclinate setulae, generally dull, creamy white, contrasted with face. Gena moderately high, gena-to-eye ratio 0.17. *Thorax*: Mesonotum uniformly faintly grayish black, finely microtomentose, lacking stripes; presutural supra-alar seta well developed. Scutellum dorsally covered with fine, sparse setulae, sometimes almost bare. Wing completely hyaline, lacking pattern of spots; vein R_2+3_ with apical portion a continued extension of angle at merger with costa; costal vein ratio 0.50-0.53; M vein ratio 0.57-0.63. Forefemur with posteroventral setae slender, not stout and peg-like; femora and tibiae grayish black to black, apices of tibiae yellowish; tarsi entirely yellowish or with apical 1-2 tarsomeres darkened. *Abdomen*: Tergites more sparsely microtomentose than mesonotum, shinier black or brown, especially laterally and mostly of tergites 4 and 5. Male terminalia (Figs 114–117): Epandrium in posterior view (Fig. 114) roundly U-shaped, except for ventral gap, oval, widest a midheight, dorsal arch very narrow, gap at ventral margin widely and shallowly U-shaped with lateral margins becoming wider ventrally, each lateral arm widest ventrally with short, medial extension, almost touching opposite medial extension, ventral extension bearing numerous setulae loosely organized as a group; cercus hemispherical, tapered ventrally to narrowly rounded apex, more setulose dorsally; gonite in lateral view (Fig. 117) robustly rod-like, almost straight, wider toward hypandrium, in ventral view (Fig. 116) shallowly curved with extension toward aedeagal base tapered to a narrow apex, apex toward hypandrium widest, with a medial, blunt, short extension; aedeagus in lateral view (Fig. 117) elongate, narrowly triangular, tapered evenly to narrowly rounded apex, in ventral view (Fig. 116) as an elongate, very narrow, rod-like structure, widest sub-basally, thereafter tapered to apex, apex with a short nipple; phallapodeme in lateral view (Fig. 117) more or less triangular, with moderately long, narrow extensions toward aedeagal base and hypandrium, keel distinct, relatively narrow, somewhat blunt apically; hypandrium in lateral view (Fig. 117) generally narrow, rod-like, basal third obtusely angulate, narrowed, digitiform, apical 2/3 narrowly rectangular, in ventral view (Fig. 116) as a very broad, robust H with short arms, emarginate anteriorly and posteriorly, anterior emargination shallow, posterior emargination more deeply excavate, broadly and rounded U to V-shaped.

###### Type material.

The holotype female of *Discocerina aracataca* Cresson is labeled “Colombia Ujhelyi/Aracataca 1912. II./825/Holo-TYPE Discocerina ARACATACA E. T. Cresson Jr [red; “Discocerina ARACATACA” handwritten].” The holotype is double mounted (minuten pin in a thin rectangular piece of foam), is in excellent condition, and is deposited in the ANSP (6541).

###### Type locality.

Colombia. Magdalena: Aracataca (10°35.6'N, 74°12'W).

###### Other specimens examined.

ARGENTINA. **Chubut**: El Hoyo (42°3.8'S, 71°31.6'W), 21 Jan 1965, A. Kovacks (1♀; AMNH). **Río Negro**: Correntoso (41°5.94'S, 71°26.65'W), Nov 1926, R. C. & E. Shannon (1♂, 1♀; USNM); Lago Nahuel Huapi (east end; 41°06.5'S, 71°12.4'W) (1♂; BMNH); Puerto Blest (41°2.6'S, 71°49.6'W), 2 Dec 1926, R. C. & E. Shannon (1♂, 1♀; USNM); San Carlos de Bariloche (41°06.5'S, 71°12.4'W) (1♂; BMNH); Nov 1926, R. C. & E. Shannon (4♂, 2♀; USNM).

CHILE. **Araucaína**: Angol (37°48'S, 72°43'W), 15 Set 1931, D. S. Bullock (1♂; USNM). **Aysen**: Puerto Puyuguapi (44°19.5'S, 72°33.5'W), Feb, 13 Out 1939, G. H. Schwable (1♀; USNM). **Bío Bío**: El Abanico (37°20'S, 71°31W), 31 Dec 1950, A. E. Michelbacher, E. S. Ross (1♀; USNM); Nuble (40 km E San Carlos; 36°24.5'S, 71°31.5'W), 23 Dec 1950, A. E. Michelbacher, E. S. Ross (4♂, 2♀; USNM); Santa Barbara (25 km E; 37°29.3'S, 72°4.1'W; 350 m), 24 Jan 1978, W. N. Mathis (12♂, 1♀; USNM). **Coquimbo**: Incahuasi (29°13.6'S, 71°0.7'W), 30 Sep 1952, P. G. Kuschel (3♂; USNM); La Serena (50 km S; 30°21.25'S, 71°15.1'W), 1 Dec 1950, A. E. Michelbacher, E. S. Ross (1♀; USNM). **Lanquihue**: Casa Pangue (41°03'S, 71° 52'W), Dec 1926, R. C. & E. Shannon (1♂, 3♀; USNM); Castro (41°27.7'S, 72°56.1'W) (ex.; BMNH); Ensenada (41°12.6'S, 72°32.3'W) (ex.; BMNH); Los Riscos (41°13.7'S, 72°44.7'W), 14 Sep 1954, P. G. Kuschel (2♂, 1♀; USNM); Peulla (41°28'S, 72°57.7'W) (1♂; BMNH); Puerto Montt (41°28'S, 72°56'W), Dec 1926, R. C. & E. Shannon (2♂, 1♀; USNM); Puerto Varas (41°18.6'S, 72°59.6'W) (2♂; BMNH); Dec 1926, R. C. & E. Shannon (2♂, 1♀; USNM). **Los Lagos**: Chiloé Island, Chepu (on seashore; 42°5'S, 73°59.65'W), Oct 1958, G. Kuschel (11♂, 3♀; USNM); Chiloé Island, Ancud (41°52'S, 73°50'W), 20–28 Jan, 1952, G. Kuschel. (1♀; AMNH). **Los Rios**: Valdivia (25 km. N; 39°35.56'S, 73°14.55'W), 26 Jan 1978, W. N. Mathis (2♀; USNM). **Maule**: Cajon de Rio Claro, (S. E. Los Quenes; 34°59.9'S, 70°49'W; 1000–1200m), 9 Oct 1966, E. I. Schlinger (1♂; USNM). **O’Higgins**: Río Claro (5 km N Rengo; 34°24'S, 70°52'W; 300 m), 23 Jan 1978, W. N. Mathis (16♂, 2♀; USNM). **Osorno**: Anticura (1 km W; 40°39'S, 72°10'W; 430 m), 1–6 Feb 1978, W. N. Mathis (26♂, 23♀; USNM); Anticura (4 km W; 37°40'S, 72°01'W; 400 m), 3 Feb 1978, W. N. Mathis (1♀; USNM); Anticura (6 km W; 37°40'S, 72°01'W; 400 m), 3 Feb 1978, W. N. Mathis (1♂, 1♀; USNM); Río El Gringo (40°41'S, 71°59'W; 1015 m), 13 Feb 1978, W. N. Mathis (1♂; USNM); Lago Puyehue (SE shore; 40°45'S, 72°25.2'W), 6–10 Feb 1978, W. N. Mathis (9♂, 6♀; USNM); Lago Rupanco, El Encanto (40°49'S, 72°28'W), 6 Feb 1978, W. N. Mathis (1♀; USNM); Laguna El Espejo (40°44.5'S, 72°19.67'W), 7 Feb 1978, W. N. Mathis (1♀; USNM); Laguna El Pato (40°40.6'S, 72°0.2'W; 1100 m), 13 Feb 1978, W. N. Mathis (3♀; USNM); Laguna El Toro (40°45.2'S, 72°18.5'W; 780 m), 8 Feb 1978, W. N. Mathis (3♂, 3♀; USNM); Pucatrihue (40°32.6'S, 73°43.1'W), 27–30 Jan 1978, W. N. Mathis (7♂, 9♀; USNM); Termas de Aguas Calientes (1 km SE; 40°41'S, 72°21'W; 530 m), 7–8 Feb 1978, W. N. Mathis (5♂, 5♀; USNM). **Santiago**: El Alfalfal (33°30.1'S, 70°11.7'W; 1320 m), 22 Jan 1978, W. N. Mathis (13♂, 4♀; USNM); Quebrada de la Plata, Rinconada, Maipu (33°31'S, 70°47'W; 510 m), 16 Aug 1966, M. E. Irwin (2♀; USNM); Río Maipo (7 km E San José de Maipo; 33°35.8'S, 70°22.1'W; 1065 m), 22 Jan 1978, W. N. Mathis (1♂; USNM). **Talca**: Río Lircay (11 km N Talca; 35°23'S, 71°39'W; 85 m), 23 Jan 1978, W. N. Mathis (1♀; USNM). **Valparaíso**: Marga Marga (road to Colliguay; 33° 5.6'S, 71°12.8'W), 14–15 Mar 1964, L. E. Peña (2♂, 2♀; USNM).

PERU. **Junin**: Tarma (11°25'S, 75°41.2'W; 3000 m). 11 Jul 1965, P. & B. Wygodzinsky (2♀; AMNH). **Lima**: San Jeronimo de Surco (11°53.1'S, 76°26.4'W; 1900–2100 m), 17 Aug 1965, P. & B. Wygodzinsky (2♂, 3♀; AMNH).

###### Distribution

(Fig. 81). Neotropical: Argentina (Chubut, Río Negro), Chile (Aracunaína; Aysen, Bío Bío, Coquimbo, Lanquihue, Los Lagos, Los Rios, Maule, O’Higgins, Osorno, Santiago, Talca, Valparaíso), Colombia (Magdalena), Peru (Junin, Lima).

###### Remarks.

This species is very similar and apparently closely related to *Lamproclasiopa puella* and is difficult to distinguish from that species using external characters. The diagnostic characters presented in original descriptions (anterior margin of frons yellowish orange, antenna mostly yellowish than grayish black, in opposition to *Lamproclasiopa puella*) are inconsistent, and specimens of *Lamproclasiopa puella* could be identified as *Lamproclasiopa aracataca* and vice versa using them. We propose a more reliable character: Scutellar disc covered with fine, sparse setulae, sometimes appearing almost bare. The more definitive diagnostic characters are the shapes of structures of the male terminalia, especially the wide ventral apices of the epandrium, the phallapodeme that has an extended keel, and the gonite in ventral view that is nearly truncate basally. The shape of the hypandrium in ventral view is very similar to that of *Lamproclasiopa puella* with posterior arms that are less flared.

##### Lamproclasiopa bisetulosa

Taxon classificationAnimaliaDipteraEphydridae

(Cresson)

**Figures 118–119.**
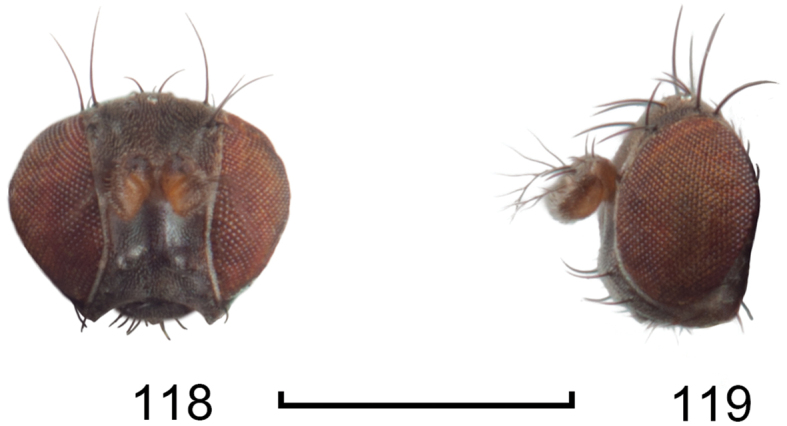
*Lamproclasiopa bisetulosa* (Cresson). (Argentina. Buenos Aires: José C. Paz) **118** head, anterior view **119** same, lateral view. Scale bar = 0.5 mm.

**Figures 120–123.**
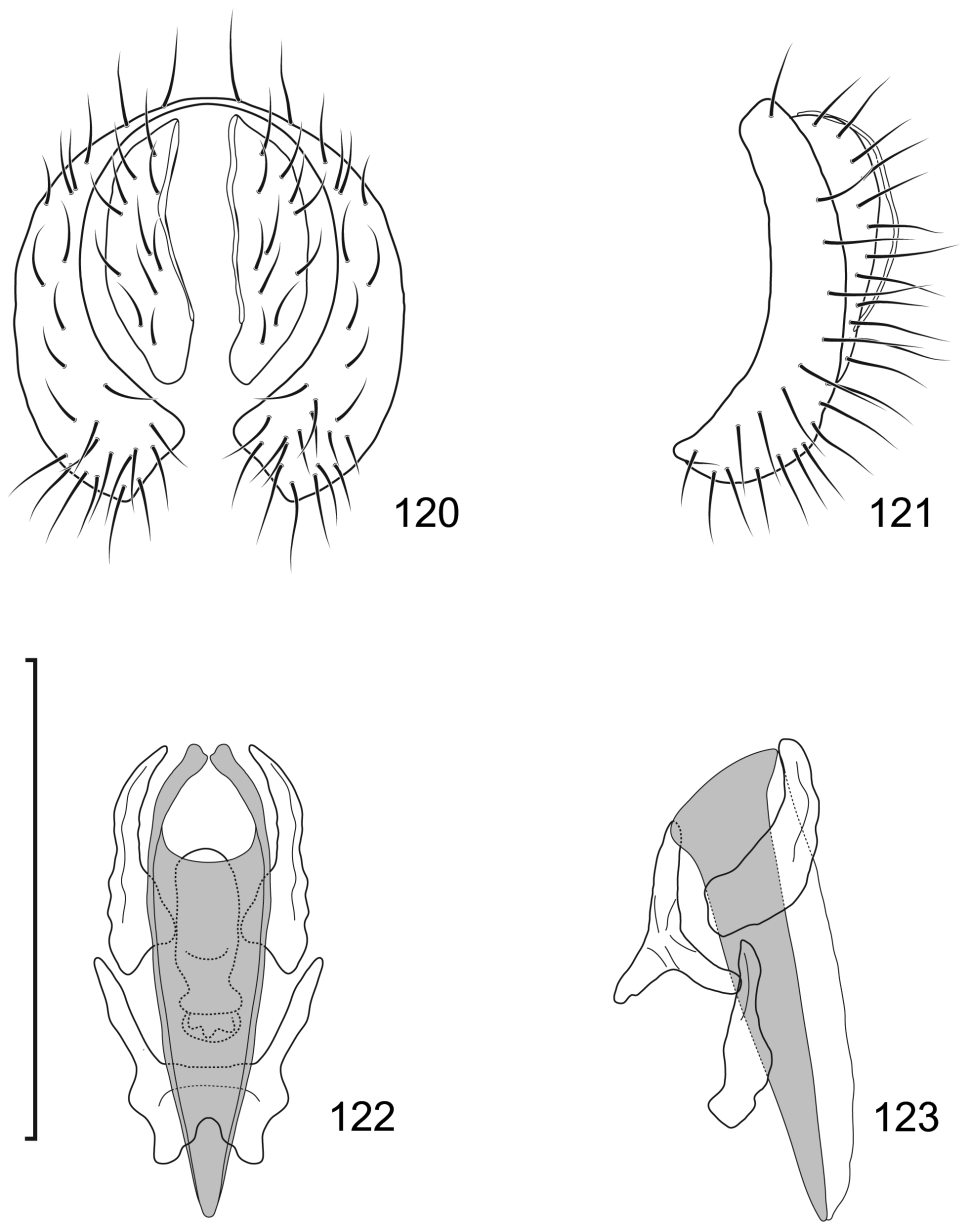
*Lamproclasiopa bisetulosa* (Cresson). (Argentina. Buenos Aires: José C. Paz) **120** epandrium and cerci, posterior view **121** same, lateral view **122** internal structures of male terminalia (aedeagus [shaded], phallapodeme, gonite, hypandrium), ventral view **123** same, lateral view. Scale bar = 0.1 mm.

Figs 118–119 , 120–123 , 139

Ditrichophora bisetulosa Cresson 1939: 7.
Discocerina (Basila) bisetulosa . Cresson 1946: 148 [generic combination]. Wirth 1968: 7 [Neotropical catalog]. Lizarralde de Grosso 1989: 24 [list, Argentina]. -Lizarralde de Grosso et al. 2011: 13 [Argentina catalog]. Mathis and Zatwarnicki 1995: 165 [world catalog].
Lamproclasiopa bisetulosa . Zatwarnicki and Mathis 2001: 39 [generic combination].

###### Diagnosis.

This species can be distinguished from congeners by the following combination of characters: Small to moderately small shore flies, body length 1.45–2.10 mm. *Head*: Frons with golden tan to slightly darker microtomentum, parafrons with slightly thinner investment of microtomentum; mesofrons evident by slight lateral lines. Antenna yellow; basal flagellomere with darker dorsal margin. Face completely and more or less uniformly silvery white microtomentose, more thinly microtomentose ventrally except for extreme ventral margin, vertical lacking stripes; 2 prominent facial setae, dorsal seta at midheight, other seta near epistomal margin; parafacial thin, more densely silvery white microtomentose than face. Gena moderately high, gena-to-eye ratio 0.12. *Thorax*: Mesonotum with golden brown microtomentum, subshiny, although less dense than microtomentum of frons; presutural supra-alar seta well developed; pleural areas more sparsely microtomentose than mesonotum, blackish brown to black, becoming less microtomentose ventrally and posteriorly, subshiny to shiny. Wing completely hyaline, lacking darkened areas; costal vein ratio 0.47–0.60; M vein ratio 0.55–0.75. Forefemur with posteroventral setae slender, not stout and peg-like; femora and tibiae grayish black to black, apical 1/4 of tibiae yellowish; tarsi yellowish, apical 1–2 tarsomeres darkened. *Abdomen*: Tergites more sparsely microtomentose than mesonotum, shinier black, especially laterally and mostly of tergites 4 and 5. Male terminalia (Figs 120–123): Epandrium in posterior view (Fig. 120) roundly U-shaped, except for ventral gap, oval, widest a midheight, dorsal arch very narrow, gap at ventral margin widely and shallowly U-shaped with lateral margins becoming wider ventrally, each lateral arm widest ventrally with short, medial extension, almost touching opposite medial extension, ventral extension bearing numerous setulae loosely organized as a group; cercus hemispherical, tapered ventrally to narrowly rounded apex, more setulose dorsally; gonite in lateral view (Fig. 123) robustly rod-like, almost straight, wider toward hypandrium, in ventral view (Fig. 122) shallowly curved with extension toward aedeagal base tapered to a narrow apex, apex toward hypandrium widest, with a medial, blunt, short extension; aedeagus in lateral view (Fig. 123) elongate, narrowly triangular, tapered evenly to narrowly rounded apex, in ventral view (Fig. 122) as an elongate, very narrow, rod-like structure, widest sub-basally, thereafter tapered to apex, apex with a short nipple; phallapodeme in lateral view (Fig. 123) as an inverted Y, each arm digitiform, process toward aedeagal base longer than other 2, in ventral view (Fig. 122) narrowly rectangular, robustly rod-like with shallow indentations toward hypandrium, keel digitiform; hypandrium in lateral view (Fig. 123) generally narrow, rod-like, essentially straight, basal third more thinly developed than anterior half, not obtusely angulate, in ventral view (Fig. 122) as a very broad, robust H with long posterior arms, lateral margins conspicuously sinuous, anterior emargination V-shaped, posterior emargination deep, broadly U-shaped.

###### Type material.

The holotype male of *Ditrichophora bisetulosa* Cresson is labeled “Paraguay Friebrig/S[an].Bernardino 1907. XI-/TYPE Ditrichophora BISETULOSA E. T. Cresson, Jr. [red; “Ditrichophora BISETULOSA” handwritten].” The holotype is double mounted (minuten pin in a thin rectangular piece of fine foam), is in good condition (some setulae missing or displaced), and is deposited in the ANSP (6574)].

###### Type locality.

Paraguay. Cordillera: San Bernardino (25°18.8'S, 57°18'W).

###### Other specimens examined.

ARGENTINA. **Buenos Aires**: Buenos Aires (34°36'S, 58°22.9'W), 21 Oct 1926, F. & M. Edwards (1♂; USNM); San Isidro (34°29.6'S, 58°32.6'W), 2 Sep 1927, R. C. Shannon (1♀; ANSP. 1♂, 1♀; USNM); José C. Paz (34°31'N, 58° 46'W), 24 Aug 1939, A. Ogloblin (2♂; USNM). **Misiones**: Santa Ana (27°22.1'S, 55°34.9'W), 9 Dec 1949, H. Aesel (1♂; USNM).

URUGUAY. **Montevideo**: Montevideo (34°53.3'S, 56°11'W), 15 Jan 1965, E. F. Legnef (2♂; USNM).

###### Distribution

(Fig. 139). Neotropical: Argentina (Buenos Aires, Misiones), Paraguay (Cordillera), Uruguay (Montevideo).

###### Remarks.

This species is very similar to *Lamproclasiopa aracataca* externally and in the shape of structures of the male terminalia. These similarities indicate that these two species are closely related. The differences, although seemingly slight, are consistent, and are the basis for our continued recognition of this species. This species is distinguished from *Lamproclasiopa aracataca* by being slightly shinier externally and by the shape of structures of the male terminalia: the hypandrium has a less well-developed base, and the phallapodeme has a narrow keel.

##### Lamproclasiopa caligosa sp. n.

Taxon classificationAnimaliaDipteraEphydridae

http://www.zoobank.org/EB6EA466-B799-4FC2-A080-25CFD27494EF

Figs 124–127 , 139

Lamproclasiopa puella of authors, not Cresson (misidentification). Zatwarnicki and Mathis 2001: 41 [illustration of male terminalia].

###### Diagnosis.

**Figures 124–127.**
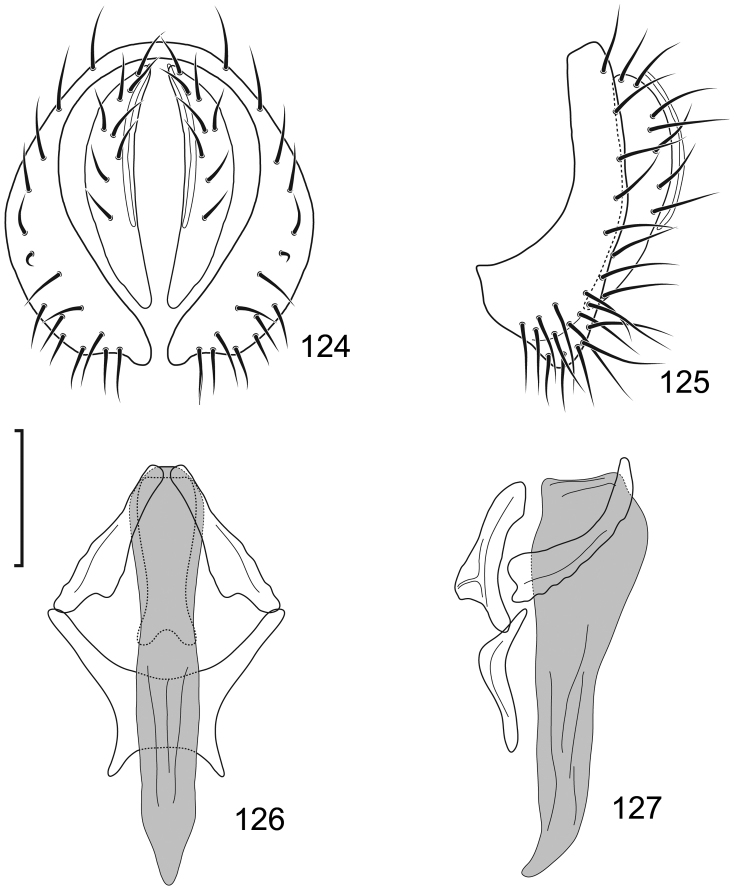
*Lamproclasiopa caligosa* sp. n. (Chile. Osorno: Anticura) **124** epandrium and cerci, posterior view **125** same, lateral view **126** internal structures of male terminalia (aedeagus [shaded], phallapodeme, gonite, hypandrium), ventral view **127** same, lateral view. Scale bar = 0.1 mm.

This species is distinguished from congeners by the following combination of characters: Small to moderately small shore flies, body length 1.80-2.80 mm. *Head*: Frons dull, uniformly grayish black concolorous with mesonotum, some specimens with anterior margin yellowish orange, except for ocellar triangle and fronto-orbital stripe slightly grayer, without distinctly marked iridescent microtomentose stripes, some specimens with anterior margin faintly reddish orange. Antenna mostly grayish black to black, only ventral margin of segments yellowish orange. Face nearly unicolorous, grayish black, not distinctively marked; parafacial bare of ventroclinate setulae, generally dull, creamy white anteriorly, grayish black ventrally, similar to facial color. Gena moderately high, gena-to-eye ratio 0.15-0.18. *Thorax*: Mesonotum uniformly faintly grayish to brownish black, finely microtomentose, faintly subshiny, lacking stripes; presutural supra-alar seta well developed. Scutellum dorsally covered with strong setulae. Wing completely hyaline to faintly infuscate, lacking pattern of spots; vein R_2+3_ with apical portion a continued extension of angle at merger with costa; costal vein ratio 0.45-0.55; M vein ratio 0.54-0.59. Forefemur with posteroventral setae slender, not stout and peg-like; femora and tibiae grayish black to black, apices of tibiae yellowish; tarsi entirely yellowish or with apical 1-2 tarsomeres darkened. *Abdomen*: Tergites more sparsely microtomentose than mesonotum, shinier black or brown, especially laterally and mostly of tergites 4 and 5. Male terminalia (Figs 124–127): Epandrium in posterior view (Fig. 124) generally oval, as high as wide, dorsal portion thin, each lateral arm gradually becoming wider ventrally, widest subapically, apex tapered, shallowly recurved, rounded pointed, ventral half with slightly increased number of setulae; cerci in posterior view (Fig. 124) elongate, thin, generally shallowly arched, ventral half generally tapered to acute point, slightly curved, setulose on dorsal 2/3; gonite in lateral view (Fig. 127) rod-like, shallowly arched, apical half toward aedeagal base digitiform, almost parallel sided, basal half wider than apical half, apex truncate with tiny, narrow emargination, basolateral margin irregularly serrate, in ventral view (Fig. 126) irregularly clavate apical half narrow, parallel sided, basal half becoming wider with subapical notch, thereafter thumb-like; aedeagus in lateral view (Fig. 127) narrowly funnel-like, wider basally, apical 2/3 tapered to acutely pointed and curved apex, in ventral view (Fig. 126) elongate, thin, tapered very gradually from base to subapex, apical portion more abruptly tapered to pointed apex; phallapodeme in lateral view (Fig. 127) shallowly L shaped, each arm tapered to narrowed apex, short arm toward hypandrium 1/3 length of longer and wider than dorsal arm toward aedeagal base, shallow keel at vertex, slightly extended, in ventral view (Fig. 126) as an inverted bottle with basal 2/3 very shallowly arched, nearly parallel sided, neck robust, slightly flared apically, apex with medial, short, narrow emargination; hypandrium in lateral view (Fig. 127) irregularly rod-like, sinuous, both apices tapered, posterior apex narrowly digitiform, anterior apex tapered to acute point, in ventral view (Fig. 126) with anterior half robustly developed, more or less quadrate, anterolateral corners slightly extended anteriorly, anterior margin shallowly emarginate, posterior extensions elongate, slightly tapered, directed posterolaterally, posterior margin deeply emarginate, widely U-shaped.

###### Type material.

The holotype male of *Lamproclasiopa caligosa* is labeled “CHILE. Osorno: Anticura (1 km W; 40°39'S, 72°10'W; 430 m), 5 Feb 1978[,], W. N. Mathis/HOLOTYPE ♂ *Lamproclasiopa caligosa* Costa, Mathis & Marinoni USNM [red].” The holotype is double mounted (minuten pin in a block of plastic), is in excellent condition, and is deposited in USNM. Five paratypes (5♂, DZUP, USNM) bear the same label data as the holotype. A male paratype (USNM) is as follows: CHILE. Bío Bío: Santa Bárbara (25 km E; 37°44.4'S, 71°47.9'W; 350 m), 24 Jan 1978, W. N. Mathis.

###### Other specimens examined.

CHILE. **Bío Bío**: Santa Barbara (25 km E; 37°29.3'S, 72°4.1'W; 350 m), 24 Jan 1978, W. N. Mathis (1♀; USNM). **Osorno**: Anticura (1 km W; 40°39'S, 72°10'W; 430 m), 1-6 Feb 1978, W. N. Mathis (19♂, 26♀; USNM); Laguna El Toro (40°45.2'S, 72°18.5'W; 780 m), 8 Feb 1978, W. N. Mathis (2♂; USNM); Termas de Aguas Calientes (1 km SE; 40°41'S, 72°21'W; 530 m), 7-8 Feb 1978, W. N. Mathis (1♂; USNM).

###### Type locality.

Chile. Osorno: Anticura (1 km W; 40°39'S, 72°10'W; 430 m).

###### Distribution

(Fig. 139). Neotropical: Chile (Bío Bío, Osorno).

###### Etymology.

The species epithet, *caligosa*, is of Latin derivation and means misty, obscure or uncertain, referring to the difficulty in distinguishing this species from congeners, especially *Lamproclasiopa puella*.

###### Remarks.

Externally, this species is very similar to *Lamproclasiopa puella*, leading to the confusion and misidentification of this species with *Lamproclasiopa puella* (Zatwarnicki and Mathis 2001). The structures that Zatwarnicki and Mathis (2001) illustrated of the so-called *Lamproclasiopa puella* are actually of this species. This species is distinguished from *Lamproclasiopa puella* by the posterior hypandrial arms being more widely separated and more flared laterally, and the aedeagal base in lateral view is wider, almost bulbous, and has a more abrupt taper after the basal one-third, and the apex is curved anteriorly and acutely pointed.

##### Lamproclasiopa curva sp. n.

Taxon classificationAnimaliaDipteraEphydridae

http://www.zoobank.org/AA7B9380-0C01-4C56-92D0-7B7EBB231538

Figs 128–131 , 139

###### Diagnosis.

**Figures 128–131.**
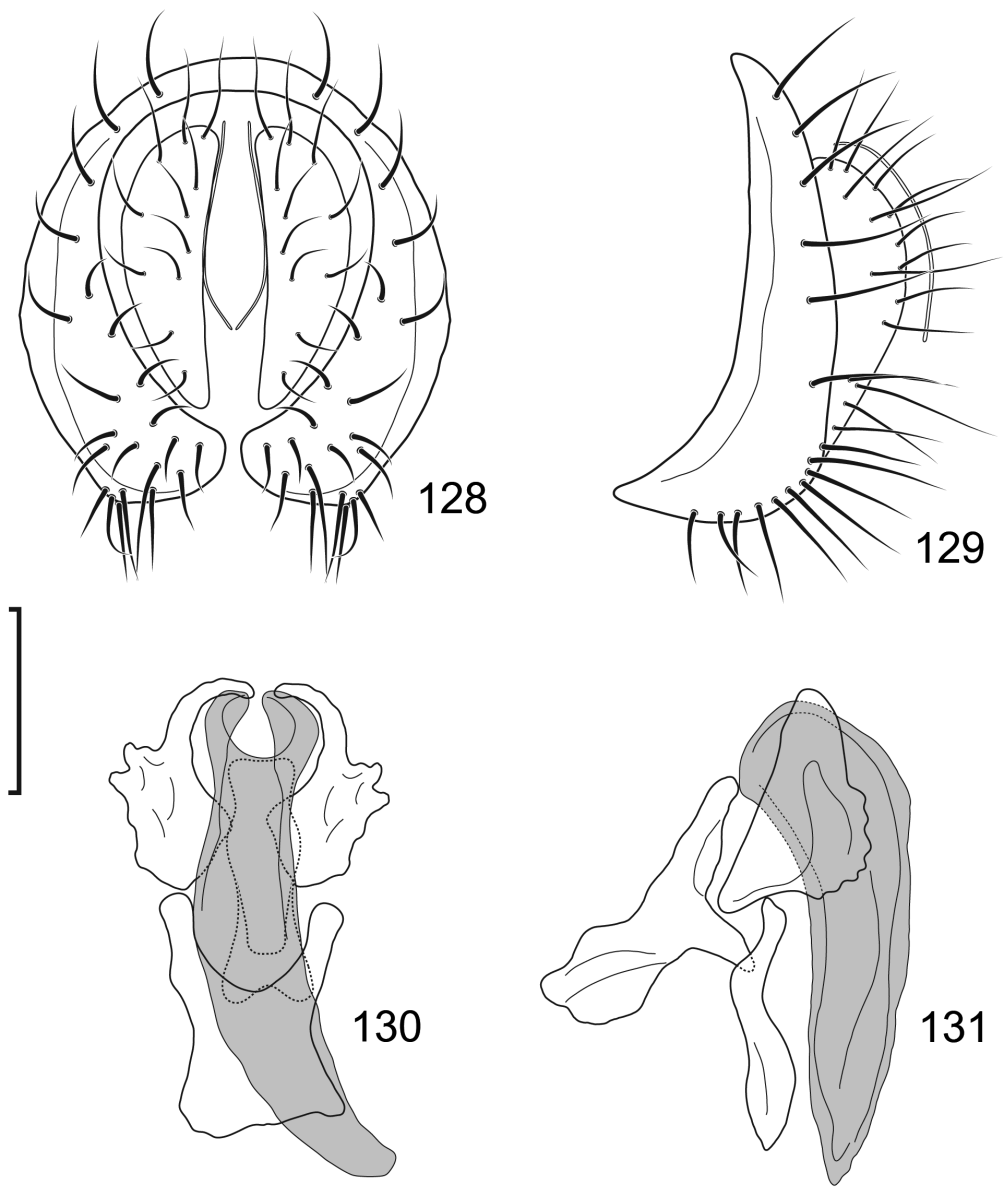
*Lamproclasiopa curva* sp. n. (Chile. Lanquihue: Casa Pangue) **128** epandrium and cerci, posterior view **129** same, lateral view **130** internal structures of male terminalia (aedeagus [shaded], phallapodeme, gonite, hypandrium), ventral view **131** same, lateral view. Scale bar = 0.1 mm.

This species is distinguished from congeners by the following combination of characters: Small to moderately small shore flies, body length 1.80–2.35 mm. *Head*: Frons dull, anterior margin yellowish orange in some degree, posterior portion grayish black, concolorous with mesonotum, some specimens with frons entirely grayish black, without distinctly marked iridescent microtomentose stripes. Antenna mostly grayish black to black, only ventral margin of segments yellowish orange. Face nearly unicolorous, blackish gray, not distinctively marked; parafacial bare of ventroclinate setulae, generally dull, creamy white, contrasted with face. Gena moderately high, gena-to-eye ratio 0.11–0.18. *Thorax*: Mesonotum uniformly faintly grayish black, finely microtomentose, lacking stripes; presutural supra-alar seta well developed. Scutellum dorsally covered with fine, sparse setulae, sometimes almost bare. Wing completely hyaline, lacking pattern of spots; vein R_2+3_ with apical portion a continued extension of angle at merger with costa; costal vein ratio 0.43–0.45; M vein ratio 0.56–0.58. Forefemur with posteroventral setae slender, not stout and peg-like; femora and tibiae grayish black to black, apices of tibiae yellowish; tarsi entirely yellowish or with apical 1–2 tarsomeres darkened. *Abdomen*: Tergites more sparsely microtomentose than mesonotum, shinier black or brown, especially laterally and mostly of tergites 4 and 5. Male terminalia (Figs 128–131): Epandrium in posterior view (Fig. 128) generally oval, almost as wide as high, dorsal portion thinner, each lateral arm gradually becoming wider ventrally, ventral half with sides almost parallel sided, apex almost truncate, shallow arched, ventral 1/4 with slightly increased number of setulae, in lateral view (Fig. 129) higher than wide, dorsal half thinner and tapered to acute apex, ventral half becoming expanded, ventral margin shallowly rounded with short, anterior extension, extension tapered to anterior point; cerci in posterior view (Fig. 128) elongate, moderately thin, curvature very slight, almost straight, ventral half generally tapered to acute point, more setulose on dorsal half, in lateral view (Fig. 129) rounded posteriorly, tapered from dorsum to ventral point; gonite in lateral view (Fig. 131) robust developed, higher than wide, with posterior margin extended, keel-like, margin serrate, anterior margin straight, in ventral view (Fig. 130) with irregularly rectangular base, dorsal extension narrow, parallel sided, curved medially; aedeagus in lateral view (Fig. 131) narrowly funnel-like, wider basally, apical 3/4 tapered to acutely pointed and straight apex, in ventral view (Fig. 130) elongate, thin, uniquely and asymmetrically curved laterally, forming banana-like structure, apical half more curved, tapered to bluntly rounded apex, basal margin bilobed with deep and rather narrow medial incision; phallapodeme in lateral view (Fig. 131) shallowly L shaped, each arm tapered to narrowed, short arm toward hypandrium 1/3 length of longer, digitiform process, keel at vertex of L, robustly developed, elongate, slightly longer than dorsal arm, in ventral view (Fig. 130) as an robustly rod-like with shallowly sinuous sides, ventral apex slightly flared and shallowly bilobed, dorsal apex truncate; hypandrium in lateral view (Fig. 131) irregularly rod-like, conspicuously sinuous, both apices tapered, in ventral view (Fig. 130) asymmetrical with one lateral half shorted than opposite lateral half, anterior margin truncate, posterior margin deeply emarginate, U-shaped with elongate posterolateral extensions.

###### Type material.

The holotype male of *Lamproclasiopa curva* is labeled “Casa Pangue (41°03'S, 71° 52'W), Llanquihue, Chile Dec1926, R&EShannon//HOLOTYPE ♂ *Lamproclasiopa curva* Costa, Mathis & Marinoni, USNM [red].” The holotype is double mounted (glued to a paper triangle) and is in good condition (abdomen removed, dissected, and in an attached microvial) and deposited in the USNM. One paratype (1♂; USNM) bears the same label data as the holotype. Other paratypes are as follows: CHILE: **Los Lagos**: Chiloé Island, Chepu (on seashore; 42°5'S, 73°59.65'W), Oct 1958, G. Kuschel (4♂; USNM).

**Other specimen examined.** CHILE. **Malleco**: Angol (37°48'S, 72°43'W), 18 Oct 1931, D. S. Bullock (1♂; USNM).

###### Type locality.

Chile. Lanquihue: Casa Pangue (41°03'S, 71° 52'W).

###### Distribution

(Fig. 139). Neotropical: Chile (Lanquihue, Los Lagos, Malleco).

###### Etymology.

The species epithet, *curva*, is of Latin derivation and means curved, bent, or arched, referring to the curved aedeagus of this species.

###### Remarks.

Externally, this species is very similar to *Lamproclasiopa aracataca* and *Lamproclasiopa puella*, and we primarily rely on structures of the male terminalia to distinguish between these three species. The most obvious distinguishing characters are the asymmetry of the aedeagus and hypandrium in ventral view. The curved aedeagus is the basis for this species name. Other distinguishing characters of this species are the extended, narrowly rectangular keel of the phallapodeme and the gonal width with serrations along some of its posterior margin.

##### Lamproclasiopa fumipennis

Taxon classificationAnimaliaDipteraEphydridae

(Wirth)

Figs 132 , 139

Discocerina (Basila) fumipennis Wirth 1955: 53; 1968: 7 [Neotropical catalog]. Mathis and Zatwarnicki 1995: 165 [world catalog].
Lamproclasiopa fumipennis . Zatwarnicki and Mathis 2001: 39 [generic combination].

###### Diagnosis

**Figure 132.**
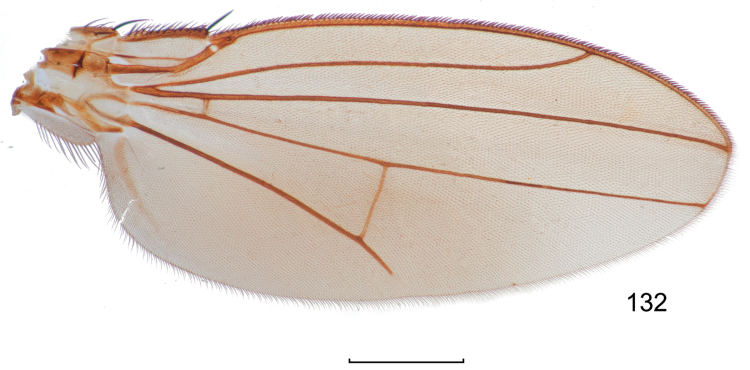
*Lamproclasiopa fumipennis* (Wirth). (Chile. Valparaíso: Juan Fernández Islands) Wing. Scale bar = 0.5 mm.

(based on Wirth’s original description). This species is distinguished from other congeners by the following combination of characters: Moderately small shore flies, body length about 3.00 mm; generally subshiny, blackish; sides of body with more or less dull brown microtomentum. *Head*: 1.2× broader than high. Frons 1.4× as broad as long; frons and occiput densely brown microtomentose; 1 pair of proclinate and 1 pair of reclinate fronto-orbitals, ocellar setae more widely separated than posterior ocelli, situated at a level about midway between bases of fronto-orbitals and anterior ocellus; pseudopostocellar setae 1/2 length of ocellar setae; medial and lateral vertical seta well developed. Basal flagellomere and palpus yellowish brown; arista with 5 dorsal rays. Face, parafacials, and gena gray, microtomentose; 2 pairs of strong facial setae; a row of very fine setulae at each parafacial suture; parafacial narrow, bare. *Thorax*: Mesonotal and discal setulae numerous and unordered; notopleuron and anepisternum with sparse setulae. A pair of strong humeral setae; notopleural setae strong, both pairs located near notopleural suture, anterior pair slightly closer to posterior pair than to humeral setae; presutural setae, supra-alar setae and prescutellar setae well developed; a somewhat weaker pair of postalar setae; lateral and apical pairs of scutellar setae each about as long as prescutellar setae. Wing densely brown infuscate (Fig. 132), veins blackish; costal section II 2.2× as long as section III; apex of vein R_3+4_ not noticeably curved into costa. Halter with yellow knob. Knees narrowly pale brownish; basal 2 tarsomeres yellowish, apical 3 brown; setae and setulae of legs and abdomen rather strong; no flexor armature on femora. *Abdomen*: Tergites more sparsely microtomentose than mesonotum, shinier black or brown, especially laterally and mostly of tergites 4 and 5. Male terminalia: Epandrium in posterior view generally oval, higher than wide, dorsal portion thin, gradually becoming wider ventrally, widest subapically, apex tapered, rounded pointed, apex and dorsal half bearing more setulae, in lateral view with dorsal half almost parallel sided, ventral portion expanded, with rounded ventral margin and shallow, anterior point subapically; cerci in posterior view elongate, thin, ventral half tapered to acute point, slightly curved, setulose on dorsal half, in lateral view elongate, thin, dorsal half wider than ventral portion, tapered toward ventral apex; in about as wide as long, narrower dorsally and ventrally, widest at midheight, each lateral arm widest ventrally, ventral margin mostly evenly rounded, with a shallow medioventral extension, more or less evenly setulose along length; cercus narrowly hemispherical, slightly curved, gradually tapered toward ventral apex; gonite in lateral view rod-like, shallowly arched, apices tapered, with broad, short process beyond midlength, in ventral view irregularly V-shaped, arm toward aedeagal base much longer, tapered, more basal arm short, digitiform; aedeagus in lateral view narrowly funnel-like, wider basally, apical half tapered to narrow, parallel-sided extension, in ventral view elongate, thin, tapered very gradually from base to apex, narrow apex rounded, base arched; phallapodeme in lateral view L shaped, each arm tapered to narrowed apex, keel at angle, slightly extended, in ventral view narrowly spindle shaped, apices expanded, end toward hypandrium bifurcate; hypandrium in lateral view rod-like, shallowly arched, in ventral view with anterior half robustly developed, more or less quadrate, anterolateral corners rounded, anterior margin shallowly emarginated, posterior extensions elongate, tapered, posterior margin deeply emarginate, V-shaped.

###### Type material.

The holotype female of Discocerina (Basila) fumipennis Wirth is labeled “Chile. Juan Fernández Islands, Masatierra, Plazoleta del Yunque (33°38.8'S, 78°50.1'W); HT ♀, UMCE].” Holotype female, allotype male, Masatierra, Plazoleta del Yunque, 200 meters, 9 January 1952, (in dense forest).

###### Other specimens examined.

CHILE. **Valparaíso**: Juan Fernández Islands, Robinson Crusoe Island (Plazoleta, trail sweep; 33°38.8'S, 78°50.3'W), 1–8 Jan 1993, S. A. Marshall (17♂, 2♀; DEBU, USNM); (quebrada S side Mirador, fern forest, 20 pans; 33°38.7'S, 78°51.1'W), 1–10 Jan 1993, S. A. Marshall (1♂; DEBU); (El Yunque Trail; 33°39'S, 78°50.6'W), 9 Jan 1993, S. A. Marshall (4♂; USNM); (English Bay; 33°37.2'S 78°50.9'W), 5 Jan 1993, S. A. Marshall (1♂, 1♀; DEBU).

###### Type locality.

Chile. Valparaíso: Juan Fernández Islands, Masatierra, Plazoleta del Yunque (33°38.8'S, 78°50.1'W; 200 m; dense forest).

###### Distribution

(Fig. 139). Neotropical: Chile (Valparaíso: Juan Fernández Islands.)

###### Remarks.

Our diagnosis of this species is partially based on Wirth’s original description (1955), as we have not been given access to the holotype, which is a female, or to the male paratype, which Wirth designated as the allotype.

When Wirth (1955) described this species, he wrote the following as a comparative diagnosis. This species is (p. 54) “Most closely related to Discocerina (Basila) puella (Cresson) from Chile, but that species has the wings hyaline, the body much duller, microtomentose above and the tarsi entirely yellow. Discocerina (Basila) polita (Edwards) from Chile is a polished, metallic black species with hyaline wings.”

We concur with Wirth that this species is indeed closely related to *Lamproclasiopa puella* and suggest, further, that these two “species” may be conspecific. Externally there are some differences, as Wirth noted and as we have confirmed herein (see key and respective diagnoses). Moreover, we have observed that these external differences, although slight, are consistent. The populations are separable. The shapes of structures of the male terminalia, however, are essentially the same for *Lamproclasiopa puella* and the darkened specimens from the Juan Fernández Islands. Thus, while we have observed that a level of genetic diversification has occurred in the island populations, the question of whether it is sufficient to represent speciation remains an open question—the dilemma of diversified, allopatric populations. For the present, we are continuing to recognize the populations from the islands as a separate species, especially as we have not been able to study the type series.

Our records indicate that this is the only congener known to occur on the Juan Fernández Islands.

##### Lamproclasiopa puella

Taxon classificationAnimaliaDipteraEphydridae

(Cresson)

**Figures 133–134.**
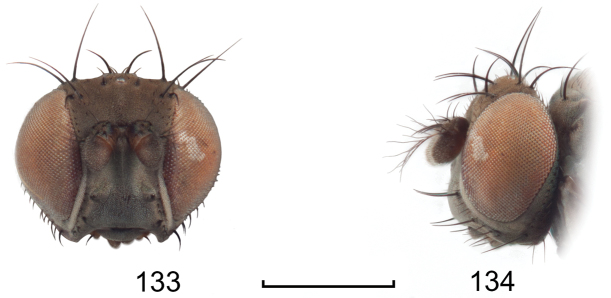
*Lamproclasiopa puella* (Cresson). (Chile. Osorno: Anticura) **133** head, anterior view **134** same, lateral view. Scale bar = 0.5 mm.

**Figures 135–138.**
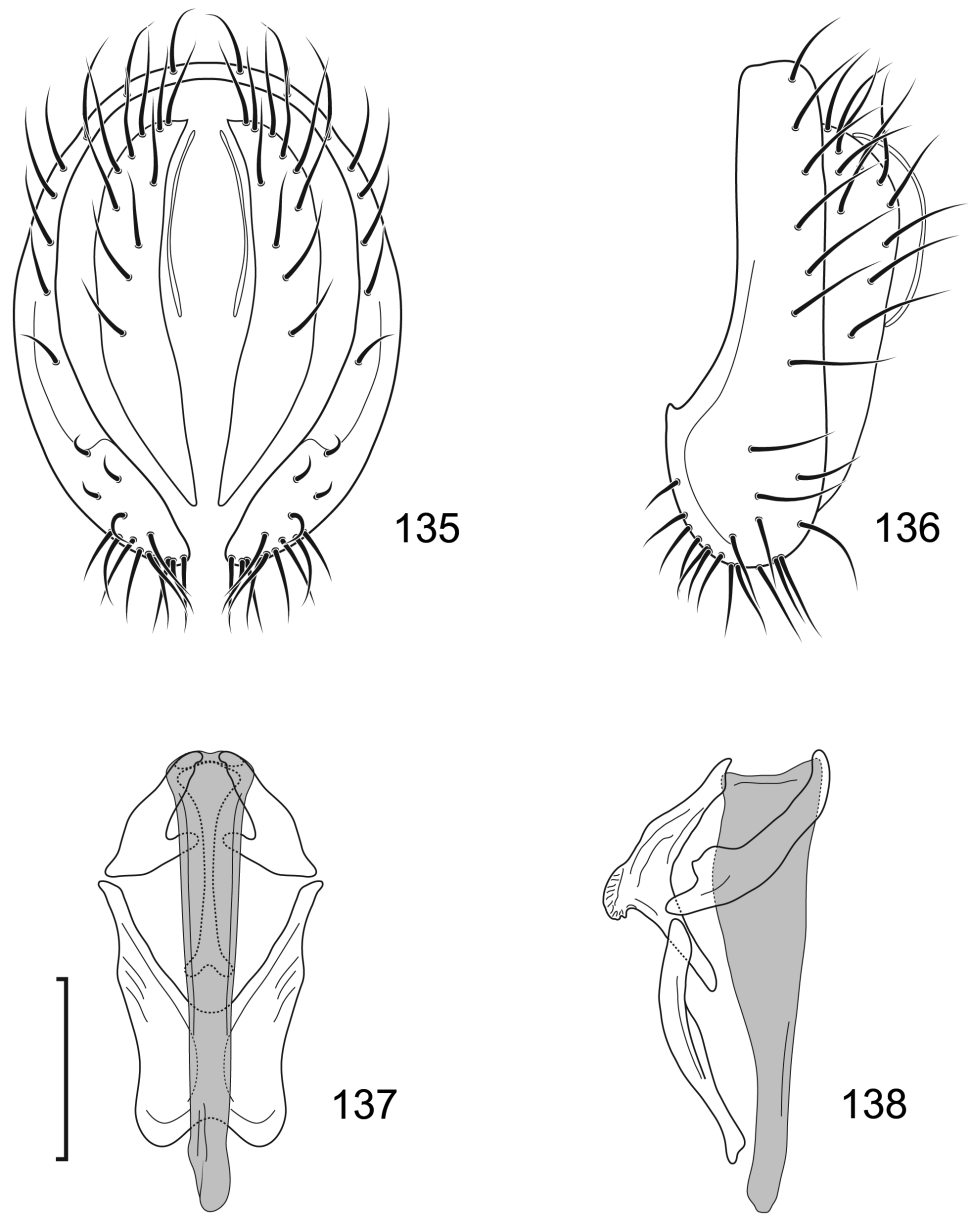
*Lamproclasiopa puella* (Cresson). (Chile. Osorno: Anticura) **135** epandrium and cerci, posterior view **136** same, lateral view **137** internal structures of male terminalia (aedeagus [shaded], phallapodeme, gonite, hypandrium), ventral view **138** same, lateral view. Scale bar = 0.1 mm.

**Figure 139.**
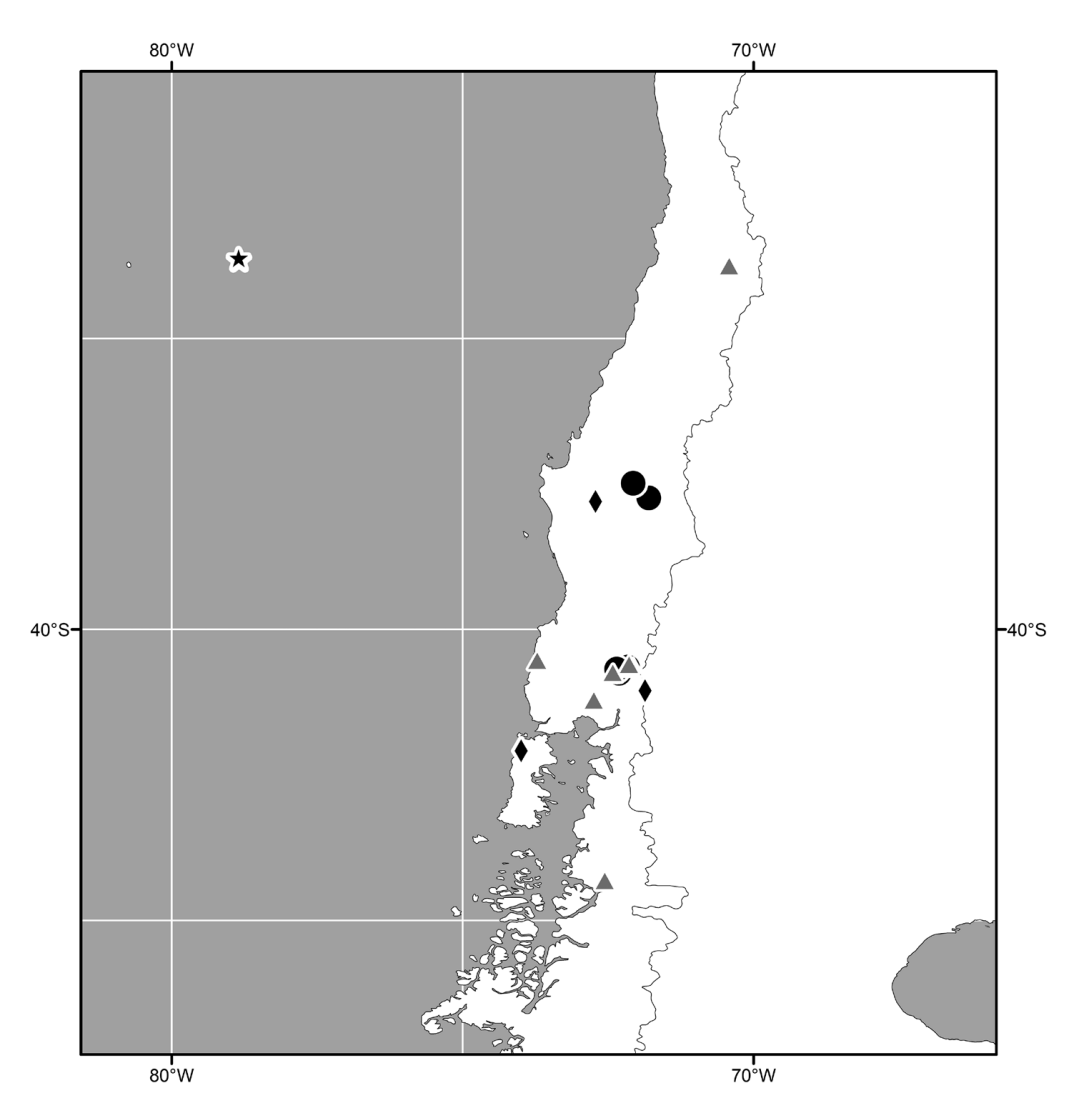
Distribution map of *Lamproclasiopa caligosa* sp. n. (●); *Lamproclasiopa curva* sp. n. (♦); *Lamproclasiopa fumipennis* (★); *Lamproclasiopa puella* (▲).

Figs 133–134 , 135–138 , 139

Ditrichophora puella Cresson 1931: 91.
Discocerina (Basila) puella . Cresson 1946: 148 [generic combination]. Wirth 1968: 7 [Neotropical catalog]. Lizarralde de Grosso 1989: 24 [list, Argentina]. –Lizarralde de Grosso et al. 2011: 13 [Argentina catalog]. Mathis and Zatwarnicki 1995: 165 [world catalog].
Lamproclasiopa puella . Zatwarnicki and Mathis 2001: 39 [generic combination].

###### Diagnosis.

This species is distinguished from congeners by the following combination of characters: Small to moderately small shore flies, body length 1.80–2.80 mm. *Head*: Frons dull, uniformly grayish black concolorous with mesonotum, some specimens with anterior margin yellowish orange, except for ocellar triangle and fronto-orbital stripe slightly grayer, without distinctly marked iridescent microtomentose stripes, some specimens with anterior margin faintly reddish orange. Antenna mostly grayish black to black, only ventral margin of segments yellowish orange. Face nearly unicolorous, grayish black, not distinctively marked; parafacial bare of ventroclinate setulae, generally dull, creamy white anteriorly, grayish black ventrally, similar to facial color. Gena moderately high, gena-to-eye ratio 0.16–0.19. *Thorax*: Mesonotum uniformly faintly grayish to brownish black, finely microtomentose, faintly subshiny, lacking stripes; presutural supra-alar seta well developed. Scutellum dorsally covered with strong setulae. Wing completely hyaline to faintly infuscate, lacking pattern of spots; vein R_2+3_ with apical portion extended at same angle to costa; costal vein ratio 0.42–0.46; M vein ratio 0.55–0.60. Forefemur with posteroventral setae slender, not stout and peg-like; femora and tibiae grayish black to black, apices of tibiae yellowish; tarsi entirely yellowish or with apical 1–2 tarsomeres darkened. *Abdomen*: Tergites more sparsely microtomentose than mesonotum, shinier black or brown, especially laterally and mostly of tergites 4 and 5. Male terminalia (Figs 135–138): Epandrium in posterior view (Fig. 135) generally oval, higher than wide, dorsal portion thin, gradually becoming wider ventrally, widest subapically, apex tapered, rounded pointed, apex and dorsal half bearing more setulae, in lateral view (Fig. 136) with dorsal half almost parallel sided, ventral portion expanded, with rounded ventral margin and shallow, anterior point subapically; cerci in posterior view (Fig. 135) elongate, thin, ventral half tapered to acute point, slightly curved, setulose on dorsal half, in lateral view (Fig. 136) elongate, thin, dorsal half wider than ventral portion, tapered toward ventral apex; in about as wide as long, narrower dorsally and ventrally, widest at midheight, each lateral arm widest ventrally, ventral margin mostly evenly rounded, with a shallow medioventral extension, more or less evenly setulose along length; cercus narrowly hemispherical, slightly curved, gradually tapered toward ventral apex; gonite in lateral view (Fig. 138) rod-like, shallowly arched, apices tapered, with broad, short process beyond midlength, in ventral view (Fig. 137) irregularly V-shaped, arm toward aedeagal base much longer, tapered, more basal arm short, digitiform; aedeagus in lateral view (Fig. 138) narrowly funnel-like, wider basally, apical half tapered to narrow, parallel-sided extension, in ventral view (Fig. 137) elongate, thin, tapered very gradually from base to apex, narrow apex rounded, base arched; phallapodeme in lateral view (Fig. 138) L shaped, each arm tapered to narrowed apex, keel at angle, slightly extended, in ventral view (Fig. 137) narrowly spindle shaped, apices expanded, end toward hypandrium bifurcate; hypandrium in lateral view (Fig. 138) rod-like, shallowly arched, in ventral view (Fig. 138) with anterior half robustly developed, more or less quadrate, anterolateral corners rounded, anterior margin shallowly emarginated, posterior extensions elongate, tapered, posterior margin deeply emarginate, V-shaped.

###### Type material.

The holotype male of *Ditrichophora puella* Cresson is labeled “HOLOTYPE/Casa Pangue 4–10.xii.1926./S.Chile: Llanquihue Prov F.&M. Edwards. B.M.1927–63./Holo-TYPE *Ditrichophora puella* E. T. Cresson Jr./NHMUK010240992”. The holotype is double mounted (glued to a plastic triangle) and is in good condition (head missing), and is deposited in BMNH.

###### Type locality.

Chile. Lanquihue: Casa Pangue (41°03'S, 71°52'W; 779 m).

###### Other specimens examined.

CHILE. **Aysen**: Puerto Puyuguapi (44°19.5'S, 72°33.5'W), Feb, 13 Out 1939, G. H. Schwable (1♀; USNM). **Lanquihue**: Los Riscos (41°13.7'S, 72°44.7'W), 14 Sep 1954, P. G. Kuschel (1♂; USNM); **Osorno**: Anticura (1 km W; 40°39'S, 72°10'W; 430 m), 1–6 Feb 1978, W. N. Mathis (11♂, 4♀; USNM); Lago Puyehue (SE shore; 40°45'S, 72°25.2'W), 6–10 Feb 1978, W. N. Mathis (3♂; USNM); Pucatrihue (40°32.6'S, 73°43.1'W), 27–30 Jan 1978, W. N. Mathis (4♂; USNM); Puyehue (20 km E.; 40°38.8'S, 72°5.1'W), 25 Jan 1951, A. E. Michelbacher, E. S. Ross (1♂; USNM); Termas de Aguas Calientes (1 km SE; 40°41'S, 72°21'W; 530 m), 7–8 Feb 1978, W. N. Mathis (13♂, 8♀; USNM); Volcan Puyehue (40°36.7'S, 72°8.4'W; 1400 m), 4 Feb 1978, W. N. Mathis (1♂; USNM).

###### Distribution

(Fig. 139). Neotropical: Chile (Aysen, Lanquihue, Osorno).

###### Remarks.

This species is challengingly similar to *Lamproclasiopa aracataca* and distinguishing between them is difficult. The diagnostic characters presented in the original descriptions (frons entirely grayish black, antenna mostly grayish black than orange, in opposition to *Lamproclasiopa aracataca*) are inconsistent, and specimens of *Lamproclasiopa puella* could easily be identified as *Lamproclasiopa aracataca* and vice versa. We dissected the male holotype to confirm the identity of *Lamproclasiopa puella*, and based on these characters we propose the more reliable, external character: scutellum covered with strong setulae. The shape of structures of the male terminalia also distinguish this species, especially the narrow aedeagus that is straight in ventral view and the less flared posterior hypandrial arms. We have studied specimens from Juan Fernández Islands and these specimens have wings slightly darker than specimens from the continent (Fig. 132). This corresponds to Wirth’s description of *Lamproclasiopa fumipennis*, but terminalia structures clearly correspond to *Lamproclasiopa puella*. As we have not been given access to the holotype of *Lamproclasiopa fumipennis*, we decided not to propose this synonymy as yet.

## Supplementary Material

XML Treatment for Discocerinini

XML Treatment for Lamproclasiopa

XML Treatment for Lamproclasiopa laevior

XML Treatment for Lamproclasiopa brunnea

XML Treatment for Lamproclasiopa hendeli

XML Treatment for Lamproclasiopa triangularis

XML Treatment for Lamproclasiopa auritunica

XML Treatment for Lamproclasiopa lapaz

XML Treatment for Lamproclasiopa polita

XML Treatment for Lamproclasiopa ecuadoriensis

XML Treatment for Lamproclasiopa zerafael

XML Treatment for Lamproclasiopa balsamae

XML Treatment for Lamproclasiopa mancha

XML Treatment for Lamproclasiopa painteri

XML Treatment for Lamproclasiopa nana

XML Treatment for Lamproclasiopa furvitibia

XML Treatment for Lamproclasiopa xanthocera

XML Treatment for Lamproclasiopa aliceae

XML Treatment for Lamproclasiopa argentipicta

XML Treatment for Lamproclasiopa nadineae

XML Treatment for Lamproclasiopa aracataca

XML Treatment for Lamproclasiopa bisetulosa

XML Treatment for Lamproclasiopa caligosa

XML Treatment for Lamproclasiopa curva

XML Treatment for Lamproclasiopa fumipennis

XML Treatment for Lamproclasiopa puella

## Checklist of species of *Lamproclasiopa* (type locality is included parenthetically for new species)

01. *Lamproclasiopa aliceae* sp. n. (United States. New Mexico. Grant: Silver City (Big Ditch; 32°46.4'N, 108°16.5'W; 1790 m)),

02. *Lamproclasiopa aracataca* (Cresson)

03. *Lamproclasiopa argentipicta* sp. n. (Costa Rica. San José. Zurquí de Moravia (10°2.8'N, 84°0.6'W))

04. *Lamproclasiopa auritunica* sp. n. (Bolívia. Oruro: Paznã (S. of the town; 18°36.2'S, 66°54.7'W, 3750 m).)

05. *Lamproclasiopa balsamae* (Cresson)

06. *Lamproclasiopa bisetulosa* (Cresson)

07. *Lamproclasiopa brunnea* sp. n. (Costa Rica. San José. Zurquí de Moravia (10°2.8'N, 84°0.6'W))

08. *Lamproclasiopa caligosa* sp. n. (Chile. Osorno: Anticura (1 km W; 40°39'S, 72°10'W; 430 m))

09. *Lamproclasiopa curva* (Chile. Los Lagos: Chiloé Island, Chepu (on seashore; 42°5'S, 73°59.65'W))

10. *Lamproclasiopa ecuadoriensis* sp. n. (Ecuador. Orellana: Río Tiputini Biodiversity Station (0°38.2'S, 76°8.9'W))

11. *Lamproclasiopa fumipennis* (Wirth)

12. *Lamproclasiopa furvitibia* sp. n. (Costa Rica. San José. Zurquí de Moravia (10°2.8'N, 84°0.6'W))

13. *Lamproclasiopa hendeli* (Wirth)

14. *Lamproclasiopa laevior* (Cresson)

15. *Lamproclasiopa lapaz* sp. n. (Bolívia. La Paz: La Paz (6 km NE; 16°25.7'S, 68°04.3'W; 4130m))

16. *Lamproclasiopa mancha* sp. n. (Brazil. Paraná: Curitiba, Universidade Federal do Paraná, Reserva Biológica (25°26.9'S, 49°14'W; 915 m))

17. *Lamproclasiopa nadineae* (Cresson)

18. *Lamproclasiopa nana* (Williston)

19. *Lamproclasiopa painteri* (Cresson)

20. *Lamproclasiopa polita* (Edwards)

21. *Lamproclasiopa puella* (Cresson)

22. *Lamproclasiopa triangularis* sp. n. (Peru. Madre de Dios: Río Manu, Pakitza (11°56.6'S, 71°16.9'W; 250 m))

23. *Lamproclasiopa xanthocera* sp. n. (Brazil. Paraná. Curitiba, Universidade Federal do Paraná, Reserva Biológica (25°26.9'S, 49°14'W; 915 m))

24. *Lamproclasiopa zerafael* sp. n. (Brazil. Amazonas: Reserva Ducke (02°55.8'S, 59°58.5'W; 40 m))
